# Efficient and accurate mixed model association tool for single-cell eQTL analysis

**DOI:** 10.1038/s41588-026-02798-2

**Published:** 2026-11-06

**Authors:** Wei Zhou, Anna S. E. Cuomo, Linfeng Hu, Angli Xue, Masahiro Kanai, Yijia Christiana Liu, Taeyeop Kang, Youngdae Kim, Alex Rodriguez, Ravi K. Madduri, Bram L. Gorissen, Grant Chau, Anne Senabouth, M. Grace Gordon, Richard Perez, Pooja Kathail, Min Cheol Kim, Chun Jimmie Ye, Chirag Krishna, Ramnik J. Xavier, Daniel G. MacArthur, Joseph E. Powell, Mark J. Daly, Benjamin M. Neale

**Affiliations:** 1 Psychiatric and Neurodevelopmental Genetics Unit, Center for Genomic Medicine, Massachusetts General Hospital, Harvard Medical School, Boston, MA USA; 2 Stanley Center for Psychiatric Research, Broad Institute of MIT and Harvard, Cambridge, MA USA; 3 Program in Medical and Population Genetics, Broad Institute of MIT and Harvard, Cambridge, MA USA; 4 Institute for Molecular Medicine Finland, University of Helsinki, Helsinki, Finland; 5 Novo Nordisk Foundation Center for Genomic Mechanisms of Disease, Broad Institute of MIT and Harvard, Cambridge, MA USA; 6 Translational Genomics Program, Garvan Institute of Medical Research, Sydney, New South Wales Australia; 7 Centre for Population Genomics, Garvan Institute of Medical Research and UNSW Sydney, Sydney, New South Wales Australia; 8 Centre for Population Genomics, Murdoch Children’s Research Institute, Melbourne, Victoria Australia; 9 School of Biomedical Sciences, University of New South Wales, Sydney, New South Wales Australia; 10 Infectious Disease and Microbiome Program, Broad Institute of MIT and Harvard, Cambridge, MA USA; 11 Department of Molecular Biology, Massachusetts General Hospital, Boston, MA USA; 12 Center for Computational and Integrative Biology, Massachusetts General Hospital, Harvard Medical School, Boston, MA USA; 13 Analytic and Translational Genetics Unit, Department of Medicine, Massachusetts General Hospital, Harvard Medical School, Boston, MA USA; 14 Department of Industrial Engineering, Ulsan National Institute of Science and Technology, Ulsan, South Korea; 15 Graduate School of Artificial Intelligence, Ulsan National Institute of Science and Technology, Ulsan, South Korea; 16 Data Science and Learning, Argonne National Laboratory, Lemont, IL USA; 17 Institute for Human Genetics, University of California, San Francisco, San Francisco, CA USA; 18 Division of Rheumatology, Department of Medicine, University of California, San Francisco, CA USA; 19 Gladstone-UCSF Institute of Genomic Immunology, San Francisco, CA USA; 20 Parker Institute for Cancer Immunotherapy, San Francisco, CA USA; 21 Arc Institute, Palo Alto, CA USA; 22 Bakar Computational Health Sciences Institute, University of California, San Francisco, San Francisco, CA USA; 23 Bakar Aging Research Institute (BARI), University of California, San Francisco, San Francisco, CA USA; 24 Department of Bioengineering and Therapeutic Sciences, University of California, San Francisco, San Francisco, CA USA; 25 Department of Epidemiology and Biostatistics, University of California, San Francisco, San Francisco, CA USA; 26 UNSW Cellular Genomics Futures Institute, University of New South Wales, Sydney, New South Wales Australia

**Keywords:** Gene expression, Software

## Abstract

Genetic effects on gene expression often vary across cell types, motivating expression quantitative trait loci (eQTL) mapping in single-cell RNA sequencing data. Existing approaches generally aggregate expression across cells, use Gaussian models poorly suited to read counts or do not scale. Here we show that SAIGE-QTL, a Poisson mixed model that directly models single-cell read counts, enables calibrated and computationally scalable eQTL mapping while accounting for donor-level and cell-level covariates and unequal numbers of cells per donor. In simulations and three single-cell datasets, SAIGE-QTL improved statistical efficiency and discovery of genes with significant eQTLs (eGenes) relative to pseudobulk methods and produced results consistent with existing single-cell models at substantially lower computational cost. In 1.2 million immune cells, SAIGE-QTL identified 48.8% more eGenes than pseudobulk analysis. SAIGE-QTL further enabled genome-wide *trans*-eQTL mapping and rare variant set-based testing, identifying rare variant associations independent of common eQTL signals. SAIGE-QTL therefore expands single-cell eQTL discovery across common, rare and *trans*-acting genetic variation.

## Abstract (web summary)

SAIGE-QTL is a framework for eQTL mapping that directly models single-cell read counts using a Poisson mixed model, achieving improved statistical efficiency and power and enabling rare variant set-based testing in the single-cell eQTL setting.

## Main

Transcriptomes have been extensively measured by RNA sequencing (RNA-seq), enabling the mapping of eQTL across multiple cell types and tissues to identify genetic variants associated with gene expression^1–3^. eQTL analyses provide evidence on functional genetic variants and facilitate understanding the molecular basis for human diseases when integrated with genome-wide association studies (GWASs)^4^. More recently, single-cell RNA-seq (scRNA-seq) methods have enabled a more fine-grained view of cellular diversity, and single-cell eQTL mapping can help uncover gene regulation at a deeper resolution of cell types^5^. Indeed, when studying the effects of genetic variation on expression using bulk transcriptomes across multiple cell types, it is virtually impossible to differentiate true transcriptomic effects from effects on cell type proportions^6^. Furthermore, bulk modeling fails to consider intra-individual cell-to-cell variability.

The first generation of single-cell eQTLs studied aggregated (typically, mean) gene expression from multiple cells per individual, using a so-called pseudobulk approach. This approach has provided important insights into the genetic basis of cell-type-specific gene expression across several tissues^7–15^ but has limitations. In particular, the pseudobulk approach does not appropriately model the intra-individual cell-to-cell variability. Therefore, it cannot leverage information shared across cells with similar profiles and loses power.

More recently, new studies have proposed approaches that model the expression levels of single cells directly^16–18^ and effectively account for key characteristics of these data: first, they can handle the presence of multiple cells from the same individual using a random effect term, and second, they appropriately model the non-normally distributed single-cell counts by adopting discrete count distributions (for example, Poisson). Yet they still have several limitations. Most notably, these methods are computationally inefficient. They are typically used only for downstream interpretation of cell state or cell transition-specific effects, following faster pseudobulk methods that are still used for the main analysis^16,17^. Current methods are not scalable genome-wide and therefore cannot be used to identify *trans*-eQTLs, even though previous studies suggest that *trans*-eQTLs explain a large proportion of gene expression variability^19,20^.

Notably, bulk and single-cell eQTL studies have historically been limited to common genetic variants (population minor allele frequency (MAF) > 5%), largely owing to data availability, with most studies relying on genotype arrays and imputation, which is limited to selected tagged (common) loci on the genome. Generally, small sample sizes also limit the exploration of rare variants. From a methodological perspective, eQTL methods typically perform single-variant tests (in which only one variant is tested at a time), which are underpowered for low-frequency variants. As the number of studies using whole-genome sequencing grows with increased sample sizes, opportunities arise to explore the role of rare genetic variations on gene expression. However, this also highlights the necessity for developing more efficient methodologies.

Here we propose SAIGE-QTL, an efficient and scalable approach for single-cell QTL mapping that addresses the aforementioned limitations. SAIGE-QTL builds on SAIGE^21^, SAIGE-GENE^22^ and SAIGE-GENE+^23^, a previously proposed suite of scalable and efficient generalized linear mixed model tools already addressing similar challenges in the context of GWAS analyses. First, SAIGE-QTL implements a mixed-effects model, thus modeling the repeated sample structure resulting from multiple cells derived from the same individual, as well as the relatedness of the individuals considered, with the inclusion of a sparse genetic relatedness matrix in the model. Second, it uses a Poisson mixed model, effectively modeling the single-cell expression profiles as discrete counts. Third, SAIGE-QTL is fast and scalable for large datasets and can efficiently test thousands of genes, tens to hundreds of cell types and millions of cells and genetic variants. Importantly, this allows for *trans*-eQTL mapping, in which we test for the effects of variants that are distant or even on a different chromosome than the target gene, as well as *cis*-eQTL mapping, in which the variants tested are in close proximity to the target gene. Finally, SAIGE-QTL can test for the effects of rare and less frequent genetic variants (MAF ≤ 5%) on gene expression through the implementation of efficient set-based tests, and it uses the saddle-point approximation (SPA)^24^ for better-controlled type I error rates.

## Results

### Overview of methods

**Extended Data Fig. 1:**
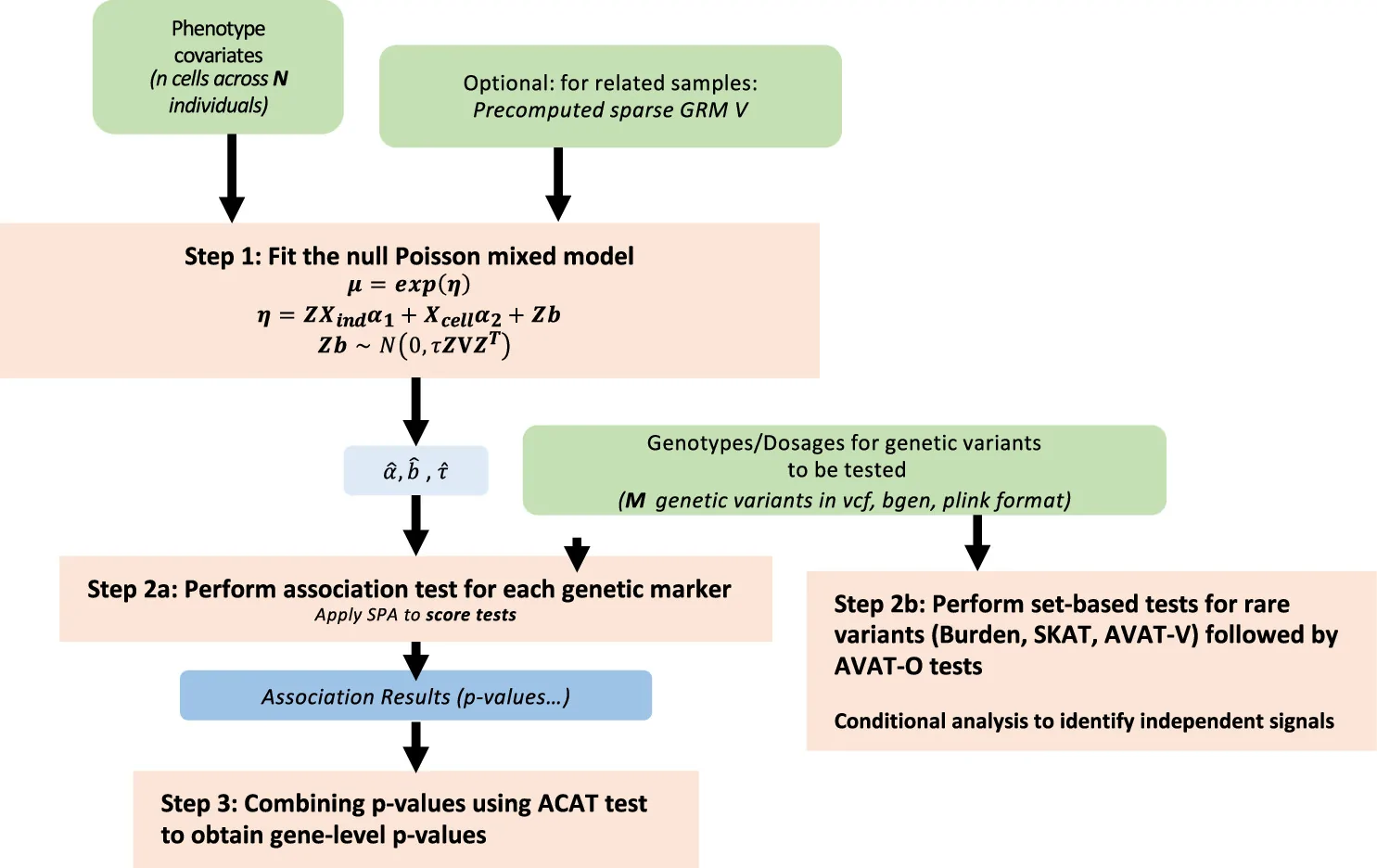
Method overview of SAIGE-QTL. Key steps, input arguments and parameters estimated are specified for our SAIGE-QTL method. Details can be found in the Methods section and Supplementary Note.

The SAIGE-QTL method contains three main steps for each gene (Extended Data Fig. 1 and Supplementary Note): (1) fitting the null Poisson mixed model, which includes individual-level covariates, such as sex, age and ancestry principal components for donors, and cell-specific covariates, such as expression-based PEER^25^ factors, as fixed effects and accounts for intra-individual cell-to-cell variability using a random effect; (2a) testing for the association between each common genetic variant (for example, MAF > 5%) and gene expression using score tests; (2b) conducting set-based tests for less frequent or rare variants with, for example, MAF ≤ 5%, including Burden tests, SKAT^26^ and ACAT-V^27^, each having superior power in various scenarios of effect directions and sparsity levels followed by an ACAT-O test^27^ to combine *P* values; (3) calculating a gene-level *P* value for each *cis*-eQTL region using the ACAT-V test^27^, which combines variant-level *P* values (from step 2a) using a Cauchy combination that is robust to the correlation among individual *P* values and the sparsity of effects. Full derivations of the null-model fitting algorithm, including the Sherman-Morrison-Woodbury (SMW) formula^28,29^ and preconditioned conjugate gradient (PCG^30^) approaches used to make step 1 efficient and scalable, the two-stage variance ratio and SPA^24^ used in step 2, are provided in Methods and Supplementary Note.

For less frequent or rare variants with MAF ≤ 5%, after fitting the null model in step 1, set-based association tests, including Burden, SKAT^26^ and ACAT-V^27^ tests, are performed in step 2b (Extended Data Fig. 1). SAIGE-QTL improves power^22,23^ by allowing for the incorporation of multiple MAF cutoffs, functional annotations and weights; for example, based on the distance to the transcription start site (dTSS), similar to approaches suggested by others^22,23,31^. Results from different tests are combined using the minimum *P* value or Cauchy combination method^27^ as used in SAIGE-GENE+^22,23^.

**Extended Data Fig. 2:**
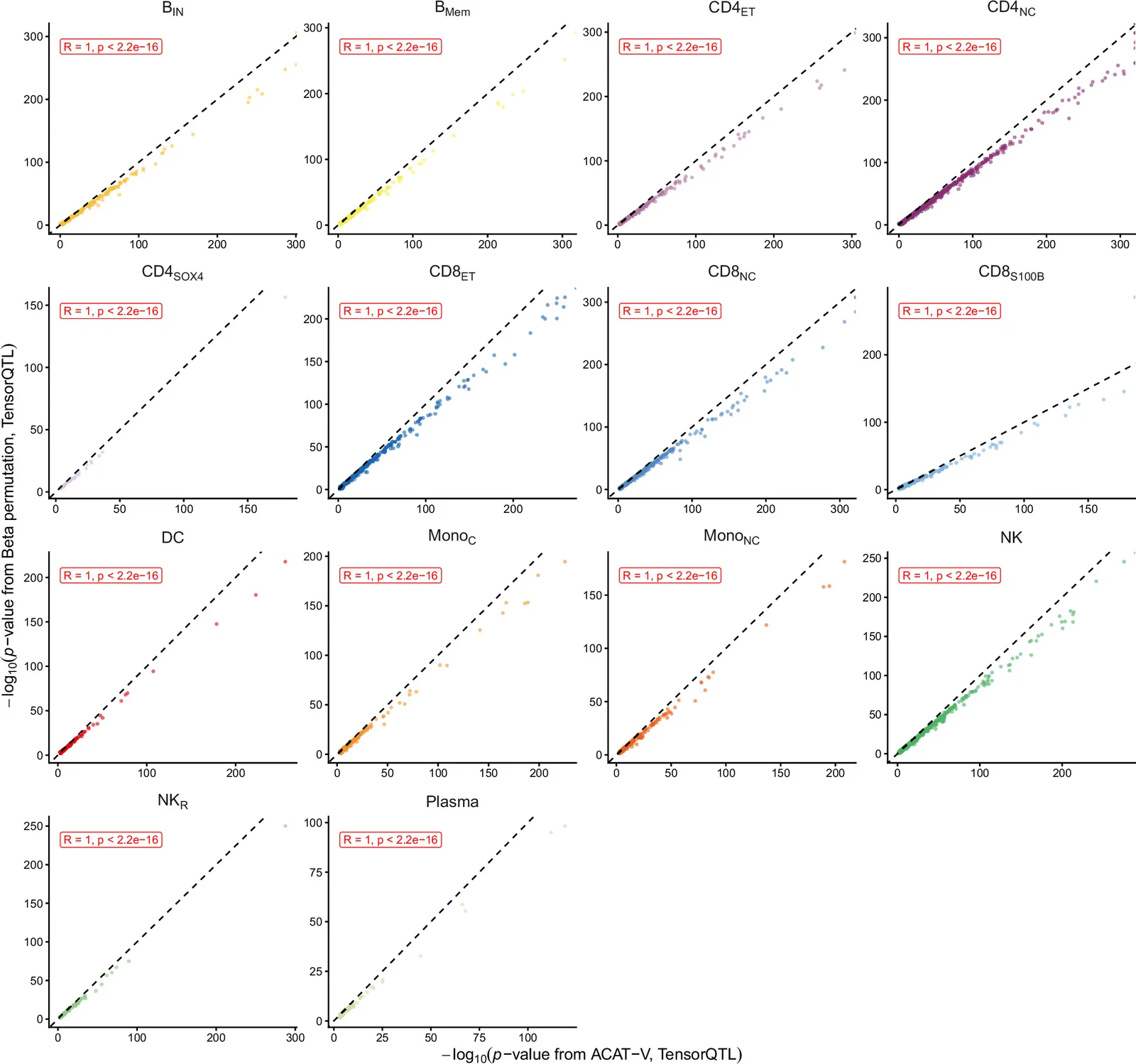
Concordance of gene-level *P*-values calculated using the Beta permutation and the Cauchy combination methods. Scatterplots showing the concordance of −log_10_ (gene-level *P*-values) calculated using different methods, for genes called an eGene by either method (FDR < 5%). Both axes show results using the nominal *P*-values from TensorQTL, but gene-level summary *P*-values are calculated using ACAT-V (Cauchy method, as we propose here, *x*-axis) vs. using the Beta approximation method typically used in TensorQTL (*y*-axis).

In step 3, a gene-level *P* value is estimated based on the Cauchy combination of variant-level *P* values. This approach was first suggested by the APEX^31^ authors and provides highly concordant gene-level *P* values compared to those obtained using the beta approximation method described in the FastQTL paper^32^ and implemented in TensorQTL^33^ (Pearson’s *R*^2^ = 1 when tested on *P* values from TensorQTL applied to OneK1K; see below and Extended Data Fig. 2).

#### Computation and memory cost

We evaluated the computation cost of SAIGE-QTL when mapping eQTLs from the largest-to-date cohort-scale scRNA-seq dataset, OneK1K^7^. In brief, the OneK1K dataset consists of scRNA-seq from over 1.2 million immune cells of 982 unique individuals, categorized into 14 distinct (sub)cell types and matched genotypes (Methods). In total, 16,250 genes are expressed in at least 10% of the donors in at least one cell type (range: 6,326–15,400 across different cell types) (Supplementary Table 1) and 12,108,282 single-base variants are called or imputed across the frequency spectrum (5,328,917 with MAF ≥ 5%; 6,779,365 with MAF < 5%) for all autosomal chromosomes (1–22).

**Extended Data Fig. 3:**
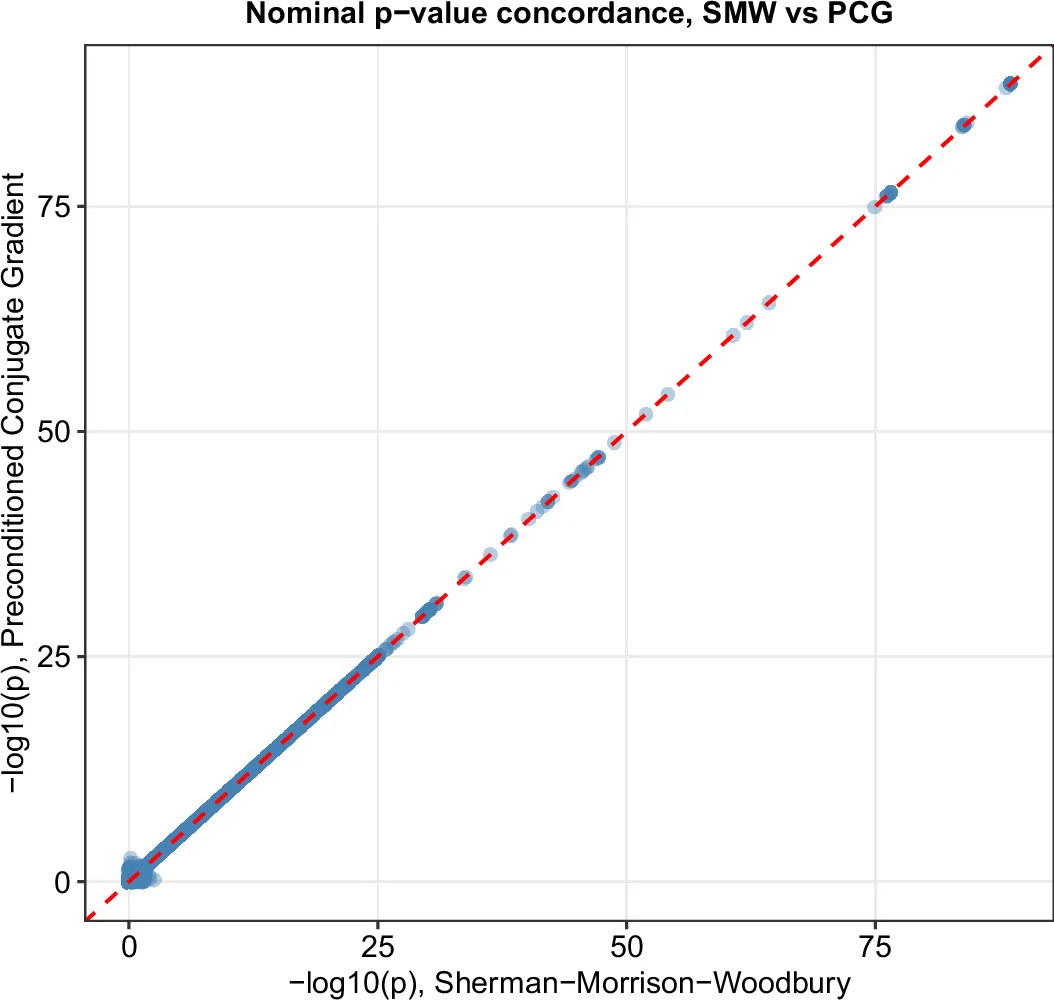
Nominal *P*-value evaluation comparing output produced from SAIGE-QTL step 1 implemented through Preconditioned Conjugate Gradient (PCG) approach and Sherman-Morrison-Woodbury (SMW) based approach. PCG-based and SMW-based approaches to solve the linear system in step 1 of SAIGE-QTL produce highly consistent results based on 100 randomly selected genes (*n* = 100) of Naive B (B_IN_) cell type from the OneK1K dataset across different sparsity levels. This demonstrates that differences in step 1 implementations of solving linear systems provide concordant numerical outcomes of SAIGE-QTL.

Using the default SMW implementation, fitting null models required 32–1,239 CPU hours and 0.3–1.6 GB of memory across cell types. SMW was 10–20 times faster than PCG with numerically consistent results (Extended Data Fig. 3 and Supplementary Table 2). Testing common *cis* variants for 20,000 genes required 28–40 CPU hours per cell type, whereas genome-wide testing of 5.3 million variants required only 630–1,013 CPU hours owing to batched genotype reading and parallel gene testing (Supplementary Table 3). Full computational benchmarks are provided in Methods and Supplementary Note.

#### Type I error rates and false-positive calibration

**Extended Data Fig. 4:**
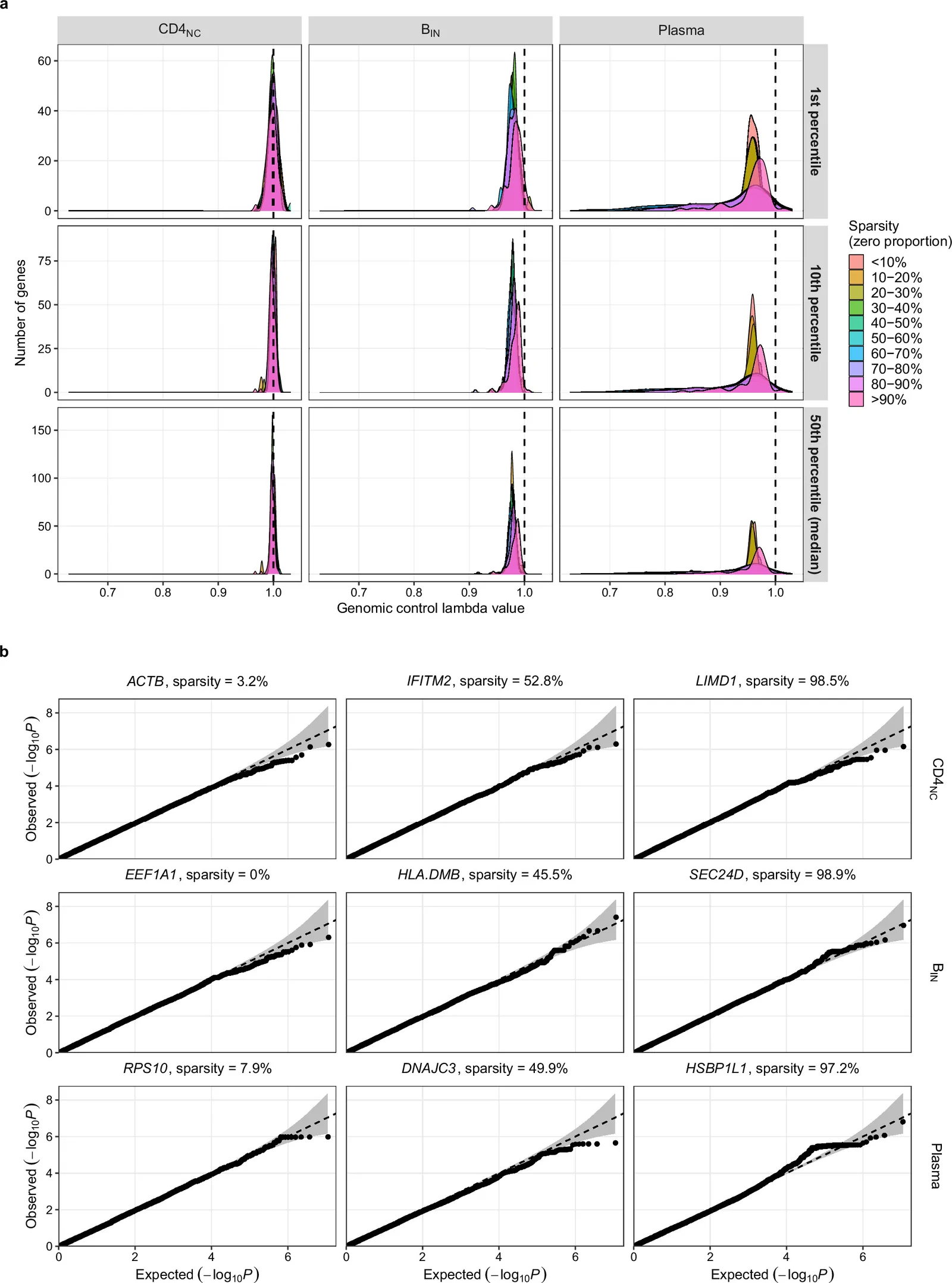
Evaluations of *P*-value calibration of SAIGE-QTL on common variants (MAF > 5%) from simulation studies under the null hypothesis. **a**, Genomic control lambda values of single-variant association tests (two-sided score tests) for 100 randomly selected genes per sparsity decile per cell type (*n* = 641, 680 and 934 genes for B_IN_, CD4_NC_ and Plasma, respectively). **b**, Quantile-quantile plots with 95% confidence intervals of 3 randomly selected genes from each sparsity group and cell type (results from chromosome 1 with 10 permutations plotted). The shaded band shows the 95% pointwise confidence interval for the order statistics of *P* values expected under the null.

**Extended Data Fig. 5:**
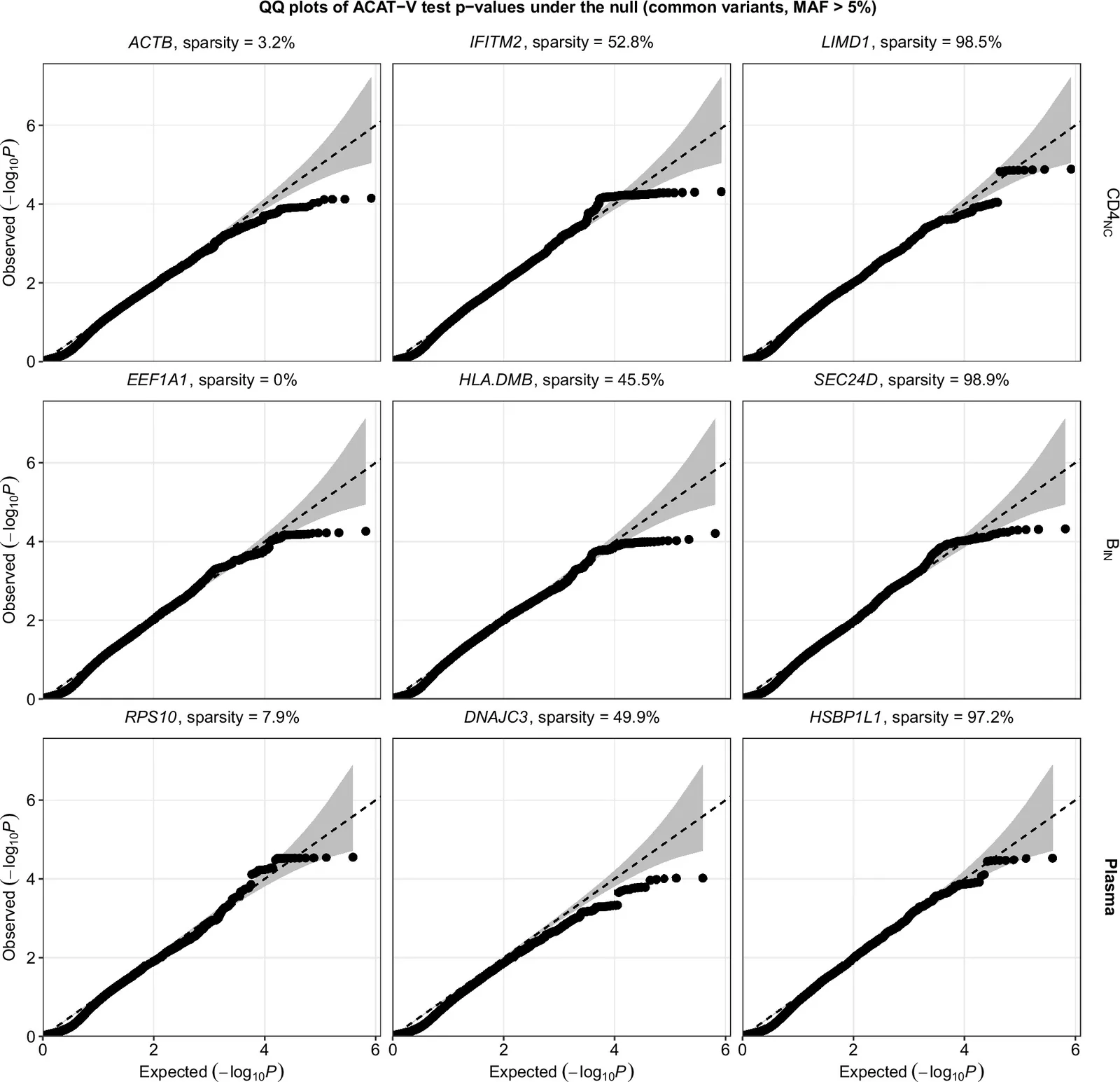
Quantile-quantile (QQ) plots with 95% confidence intervals of ACAT-V tests of common variants (MAF > 5%) on each gene region on chromosome 1 in simulation studies under the null hypothesis. Genes were randomly selected from each sparsity group (low, intermediate, high) per cell type and single-variant association tests across chromosome 1 with 250 permutations were conducted for the single-cell expression read counts of each gene (Extended Data Fig. 4b). For a *cis* window upstream and downstream of each gene body (+/-1 Mb) on chromosome 1, the ACAT-V (Cauchy combination of two-sided *P* values) test is performed to obtain a gene-level *P*-value. Three representative cell types are shown (Plasma, B_IN_, CD4_NC_).

**Extended Data Fig. 6:**
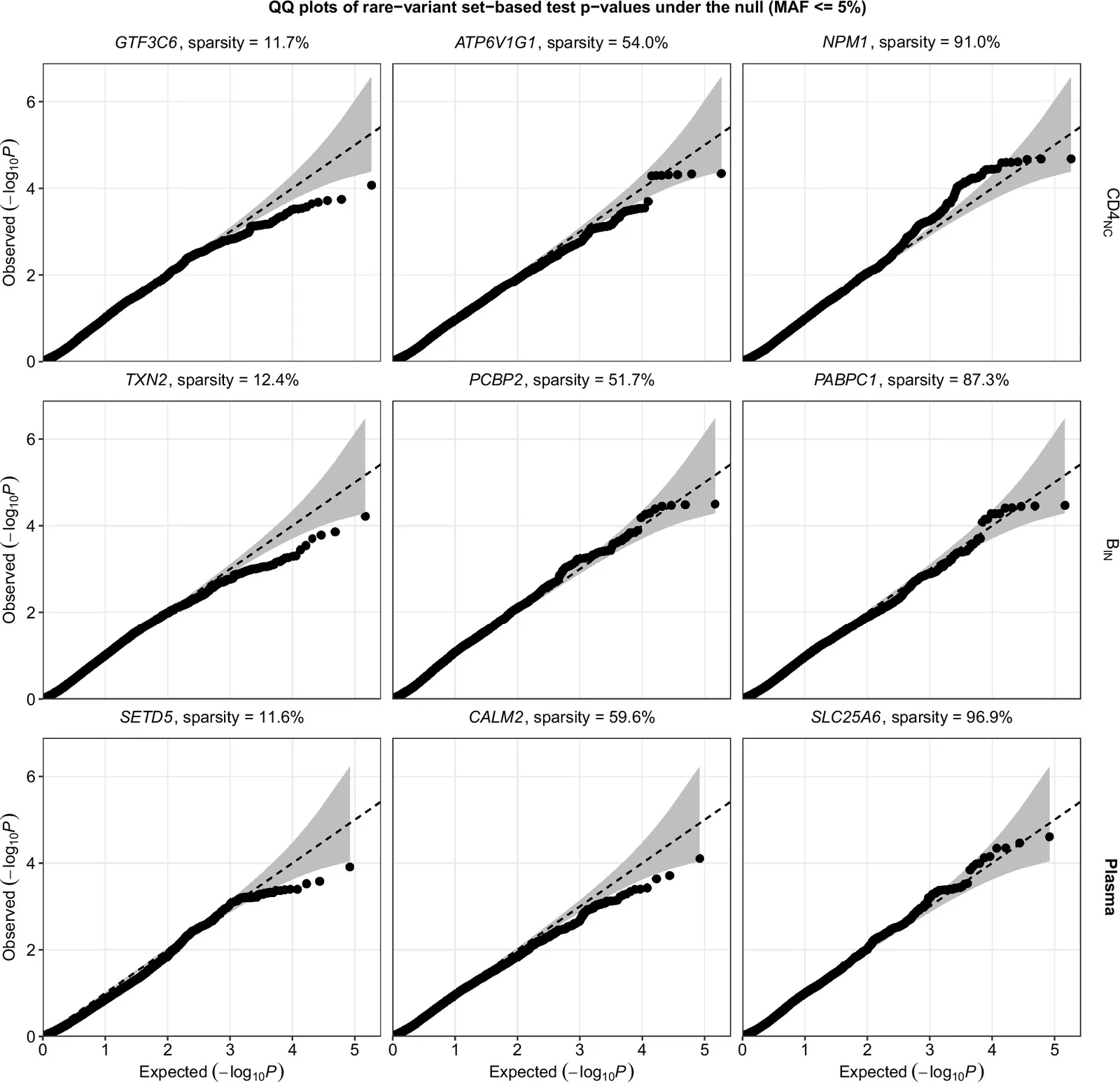
QQ plots of set-based tests of rare variants (MAF ≤ 5%) on each gene region on chromosome 1 in simulation studies under the null hypothesis. For three representative cell types (rows: CD4_NC_, B_IN_, Plasma) and three genes of increasing sparsity per cell type (columns), gene-level *P* values from the rare-variant set-based test (Cauchy/ACAT-O combination, as in Fig. 4a), under the null hypothesis of no eQTL effect. *n* per cell type (region × permutation-seed combinations): CD4_NC_, *n* = 91,357; B_IN_, *n* = 72,637; Plasma, *n* = 41,323.

We evaluated false-positive calibration using real OneK1K read counts with permuted donor genotypes across three representative cell types (CD4^+^ naive and central memory T cells (CD4_NC_), innate and naive B cells (B_IN_) and plasma) spanning a range of abundance and expression sparsity (Supplementary Table 4, Methods and Supplementary Note). Single-variant score tests were well calibrated, with slight deflation for genes of medium-to-high sparsity and in less abundant cell types (Supplementary Table 5 and Extended Data Fig. 4). Gene-level ACAT-V tests combining common variant *P* values (step 3) showed similarly slight deflation (Supplementary Table 6 and Extended Data Fig. 5). Set-based tests for rare variants (MAF ≤ 5%) were also well calibrated across sparsity groups and cell types, including at significance thresholds down to 5 × 10^−4^ (Extended Data Fig. 6).

### Association analysis of OneK1K scRNA-seq and imputed genotypes

**Fig. 1:**
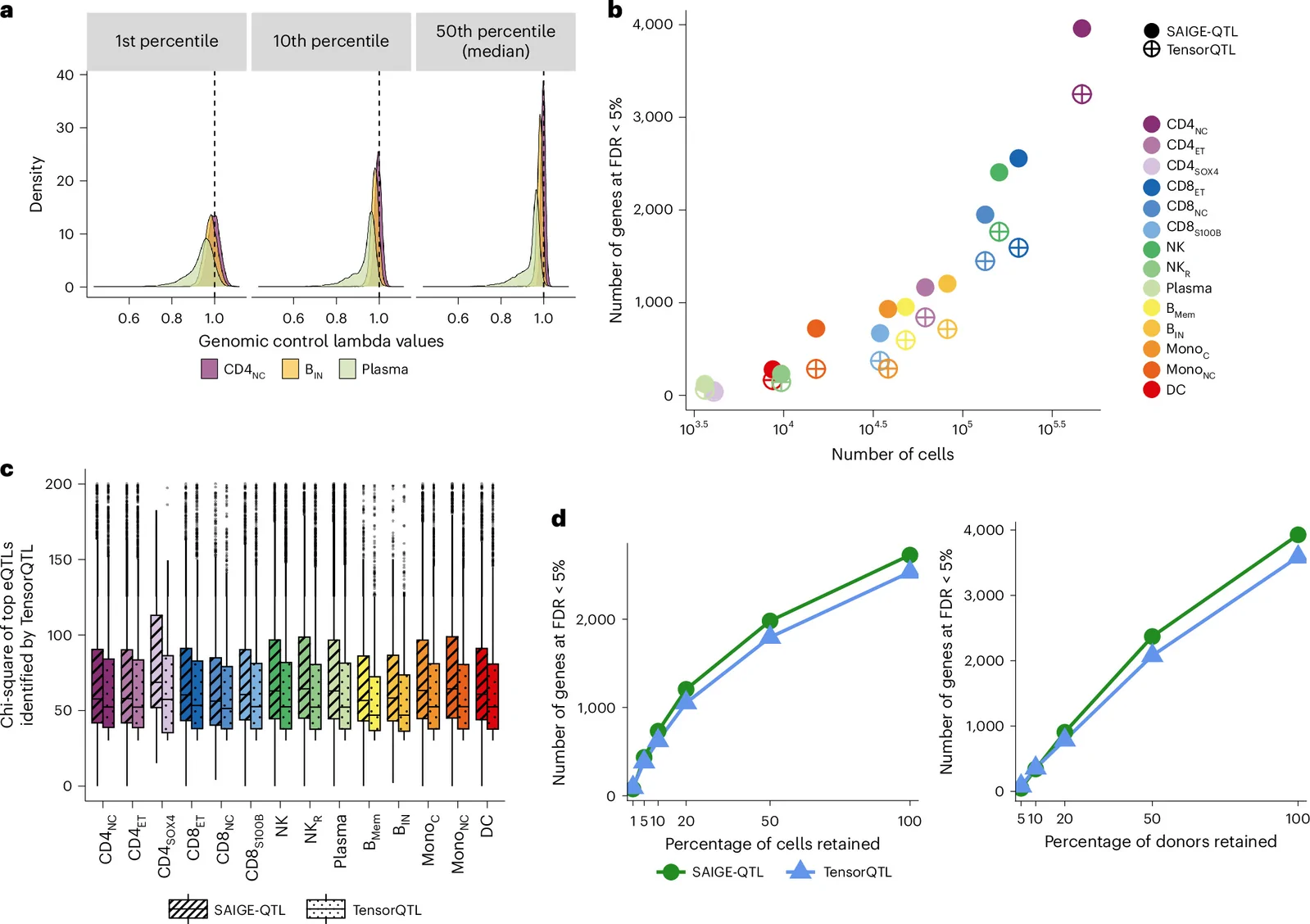
Performance evaluation of SAIGE-QTL. **a**, Calibration (genomic control lambda values) across three representative cell types (CD4_NC_, B_IN_, plasma; *n* = 13,090, 11,076 and 6,938 genes, respectively). **b**, Plot of power (number of eGenes identified at FDR < 5%, ACAT-V gene-level test) and number of cells between SAIGE-QTL (solid dots) and TensorQTL (crosses) across 14 cell types. **c**, For each of the 14 cell types, boxplots of chi-squared statistics (from two-sided single-variant score tests) for the top eQTL in SAIGE-QTL (diagonal lines) and TensorQTL (dots), restricted to genes with TensorQTL top variant *P* < 5 × 10^−8^ (exact number per cell type: CD4_NC_, *n* = 3,059; CD4_ET_ *n* = 3,626; CD4_SOX4_, *n* = 34; CD8_ET_, *n* = 2,043; CD8_NC_, *n* = 999; CD8_S100B_, *n* = 2,312; NK, *n* = 4,158; NK_R_, *n* = 3,014; plasma, *n* = 4,208; B_Mem_, *n* = 930; B_IN_, *n* = 521; Mono_C_, *n* = 2,673; Mono_NC_, *n* = 2,894; DC, *n* = 2,434). When the top variant was different between methods, the TensorQTL top variant was selected. The *y* axis is truncated at a maximum value of 200. Boxplots indicate median (center line), 25th and 75th percentiles (bounds of box) and whiskers extending to 1.5× the interquartile range; values beyond the whiskers are shown as individual points. **d**, Plots of power (number of eGenes identified at FDR < 5%, *y* axis) and percentage of cells (left) and donors (right) subsampled for the most abundant cell type (CD4_NC_; Methods). CD4_ET_, CD4^+^ T cells with an effector memory or central memory phenotype; CD4_SOX4_, CD4^+^ T cells expressing SOX4; CD8_ET_, CD8^+^ T cells with an effector memory phenotype; CD8_NC_, CD8^+^ naive and central memory T cells; CD8_S100B_, CD8^+^ T cells with expression of S100B; NK, natural killer cells; NK_R_, natural killer recruiting cells; plasma, plasma cells; B_Mem_, memory B cells; Mono_C_, classical monocytes; Mono_NC_, nonclassical monocytes; DC, dendritic cells.

**Extended Data Fig. 7:**
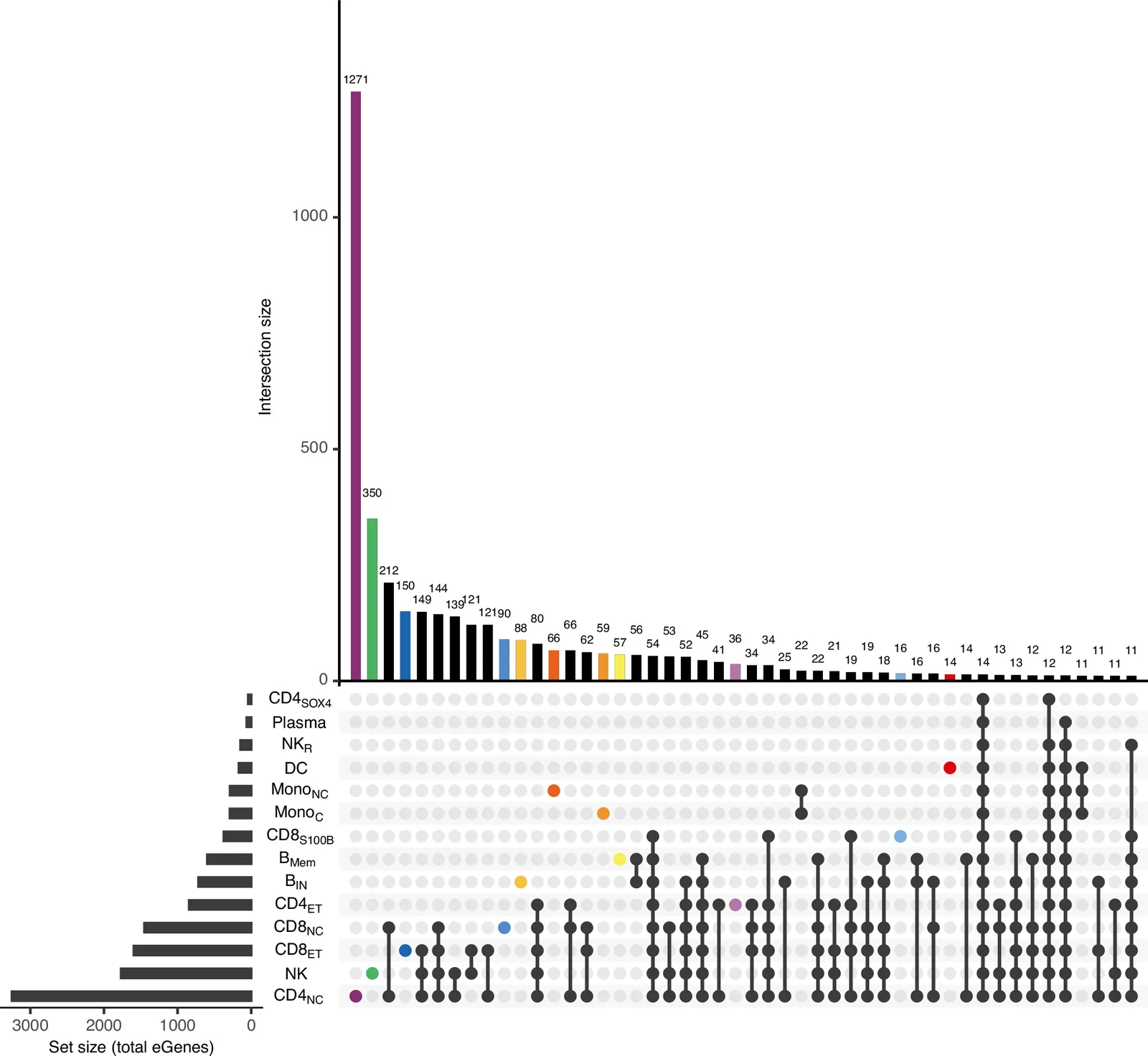
Cell type-specific eQTLs. UpSet plot showing the cell type specificity of significant eGenes (FDR < 5%) identified by SAIGE-QTL in the OneK1K dataset (*n* = 5,894 unique eGenes). Colored bars represent the number of eGenes only found in a specific cell type, while black bars represent shared signals across two or more cell types. Only intersections > 10 are shown. The UpSetR package in R was used for this figure.

To demonstrate the application of SAIGE-QTL to real-world scRNA-seq cohorts, we applied it to data from the OneK1K project^7^. We mapped *cis*-eQTLs (considering all single-nucleotide polymorphisms (SNPs) of MAF > 5% and within a ±1 Mb window around the gene body) for all genes expressed in at least 10% of the donors (in the relevant cell type) (Supplementary Table 1). We used the Poisson mixed model from SAIGE-QTL, modeling single-cell counts directly as opposed to aggregating counts at the donor level using a pseudobulk approach, as conducted in the original paper^7^ (using Matrix eQTL^34^; Methods). We used the same covariates as in the original model, correcting for sex, age, six genotype principal components and two cell-type-specific expression PEER^25^ factors from the original publication. We observed well-calibrated *P* values in SAIGE-QTL, with genomic control lambda values around one in the three representative cell types (Fig. 1a). Across all 14 cell types, SAIGE-QTL identified 17,218 eGenes (that is, genes with at least one eQTL) across all cell types (false discovery rate (FDR) < 5%, as described below and in Methods), representing 5,894 unique genes, of which 2,447 (42%) were specific to one cell type only (Extended Data Fig. 7).

#### SAIGE-QTL identified more eGenes than the pseudobulk approach

**Extended Data Fig. 8:**
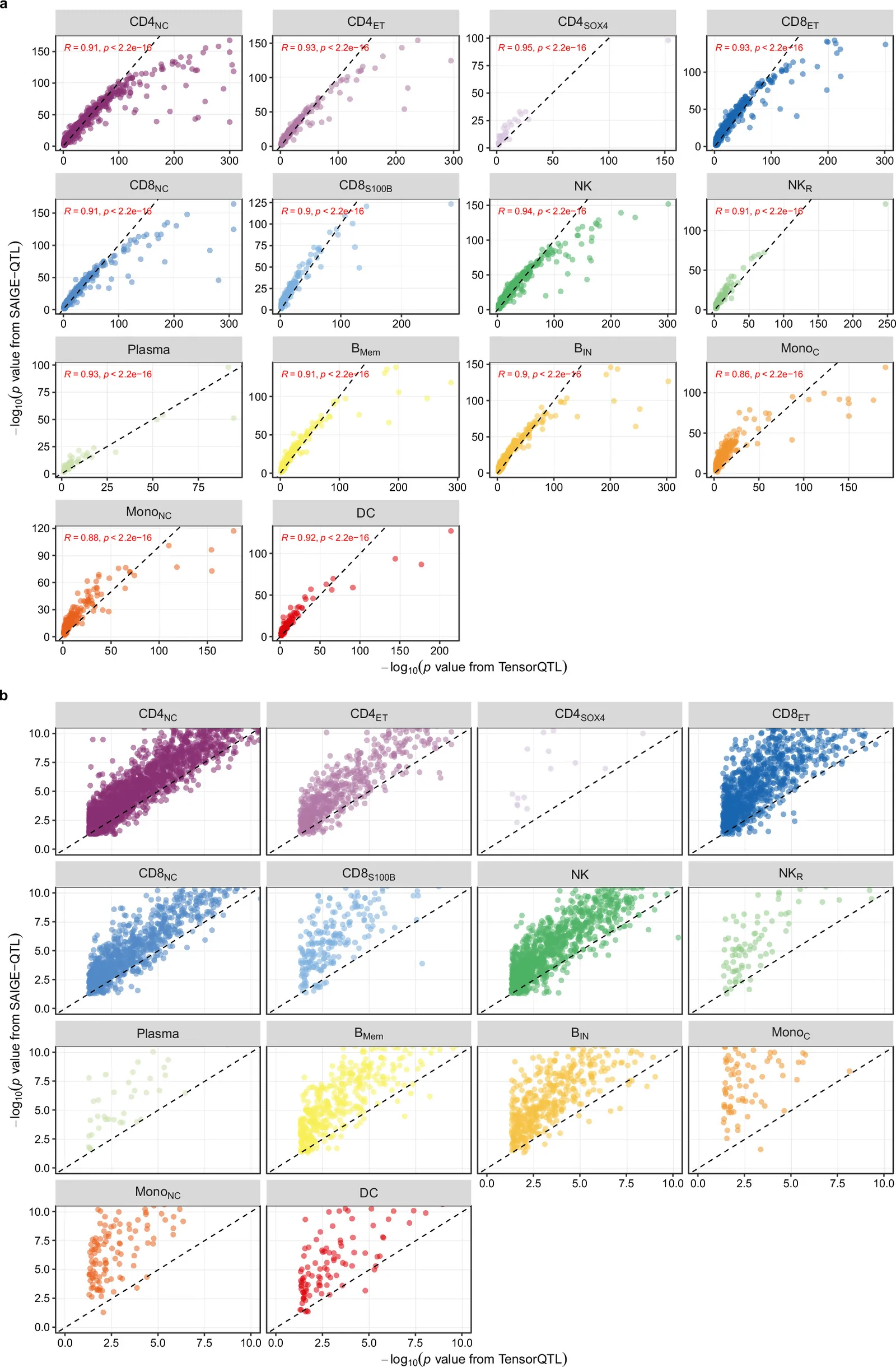
*P*-value concordance between SAIGE-QTL and TensorQTL. Scatter plots of −log_10_ (*P*-values) from TensorQTL (*x*-axis) and SAIGE-QTL (*y*-axis) are shown for each of the 14 cell types. Top variants in eGenes identified in OneK1K (FDR < 5%) are included. **a**, All variants, with Pearson correlation coefficients specified (range 0.90-0.97 across cell types). **b**, As in **a**, but zoomed to *x*-axis and *y*-axis values between 0 and 10.

**Extended Data Fig. 9:**
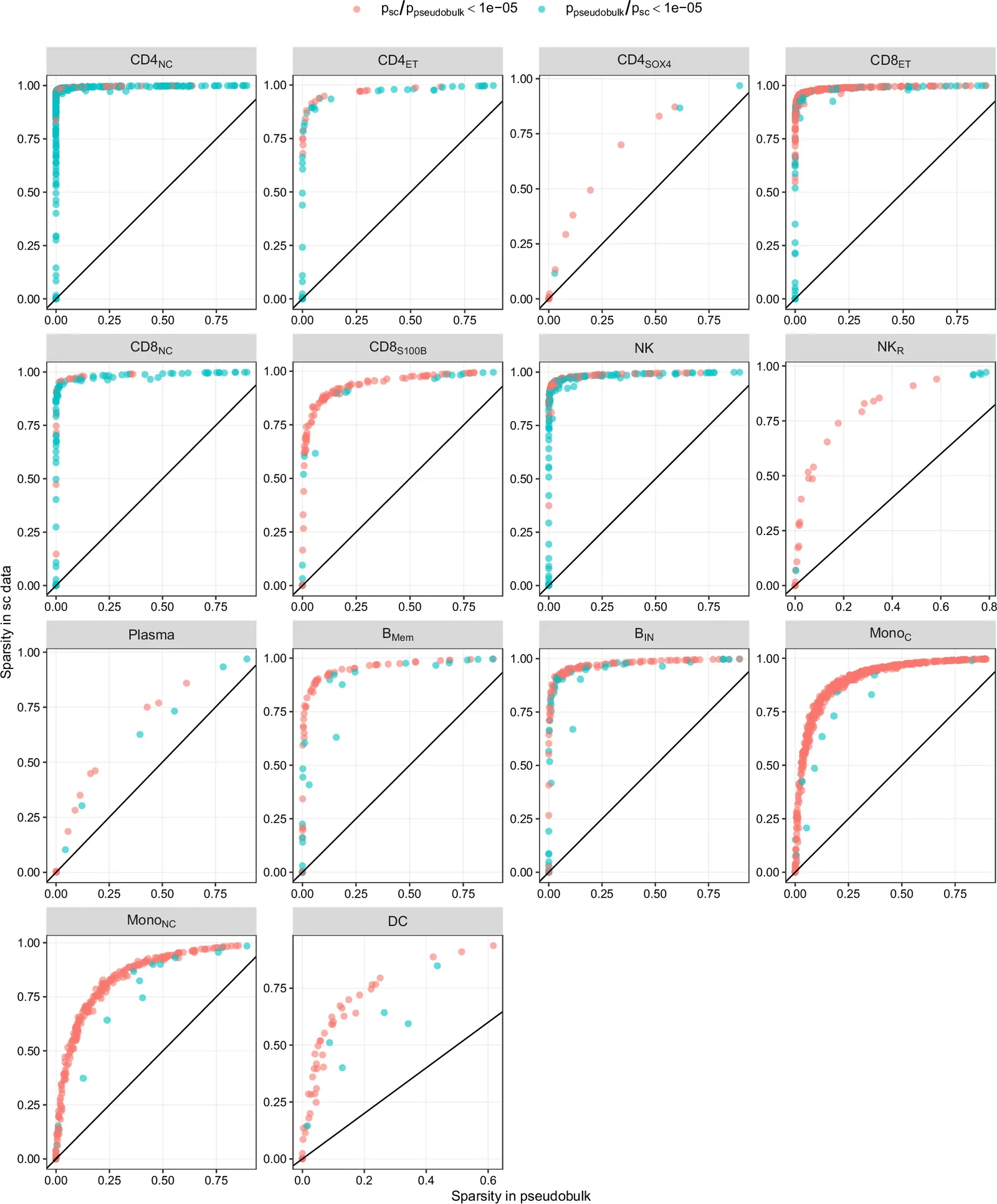
Relationship between sparsity levels and the nominal *P*-values of sc-eQTL using SAIGE-QTL and pseudobulk eQTL using TensorQTL. Sparsity level across cells (*y*-axis) is plotted against sparsity level across donors (*x*-axis) for genes where the two methods’ nominal *P* values were extremely discordant (|log_10_ (*P* from SAIGE-QTL) − log_10_ (*P* from TensorQTL)| > 5; total *n* = 2,091 gene-cell type pairs). If the top variants identified differ between methods, the TensorQTL top variant is used. Each plot represents the results for each of the 14 cell types.

**Extended Data Fig. 10:**
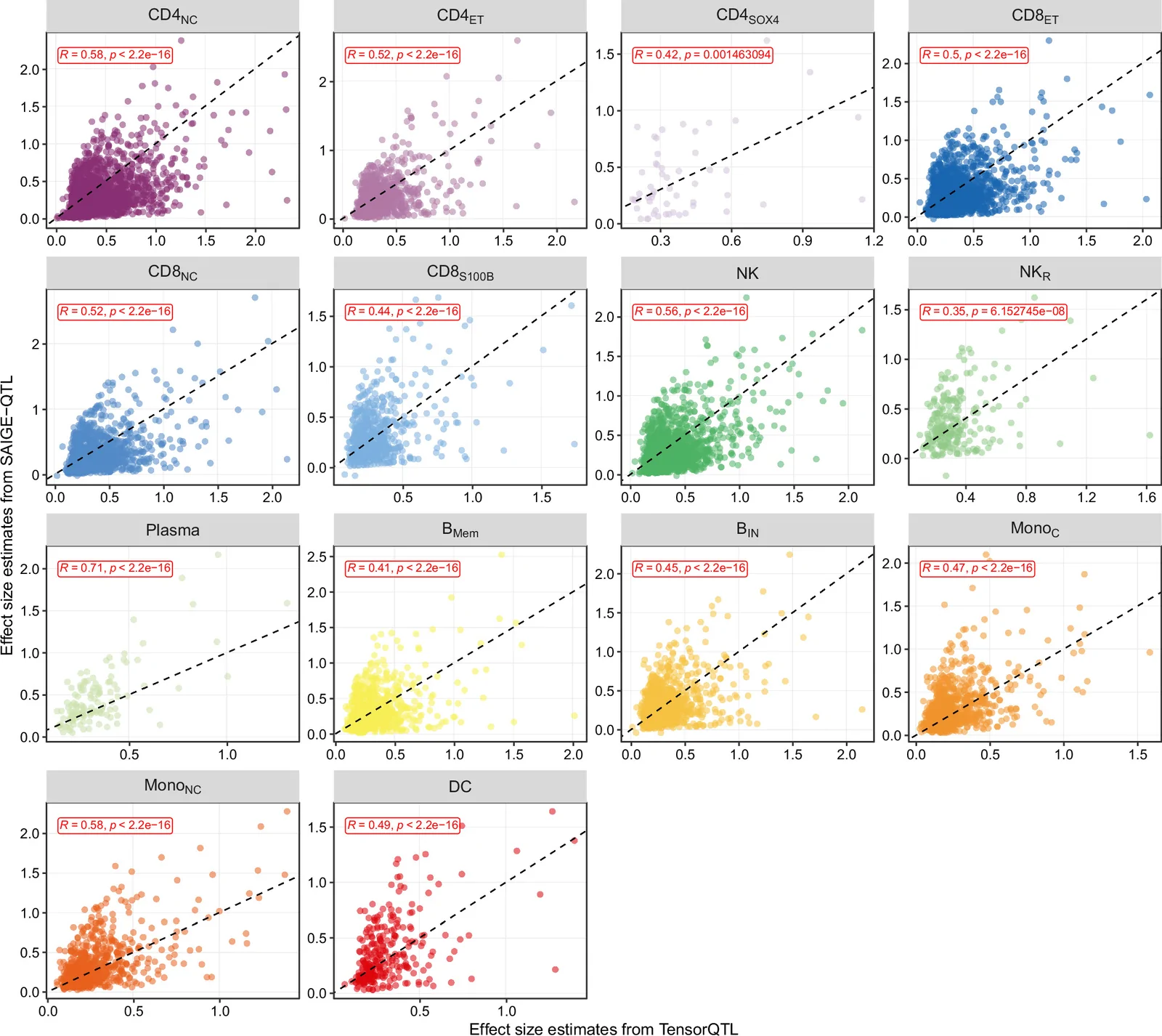
Effect size concordance between SAIGE-QTL and TensorQTL. Scatter plots of estimated effect sizes from TensorQTL (*x*-axis) and SAIGE-QTL (*y*-axis) are shown for each of the 14 cell types, with Pearson correlation coefficients specified. Top variants in SAIGE-QTL results in each eGene identified by either method are included. For each variant, the effect sizes are aligned to the same allele (the one with a positive effect in TensorQTL).

We compared SAIGE-QTL with the widely used tool TensorQTL^33^, which we ran using (mean) pseudobulk counts and the same covariates and window size as above across all 14 cell types (Methods). We obtained good concordance of the nominal *P* values between the two methods (Pearson’s correlation ranges from 0.9 to 0.97; Extended Data Fig. 8a). We also observed that TensorQTL provides more significant nominal *P* values than SAIGE-QTL for eQTLs with extremely significant *P* values (that is, *P* < 10^−100^), while SAIGE-QTL tends to have more significant *P* values than TensorQTL for those less significant eQTL signals (as shown in Extended Data Fig. 8b). Among genes with similar sparsity levels across cells, those with a higher sparsity level after pseudobulking tend to have more significant nominal association *P* values in TensorQTL than SAIGE-QTL (Extended Data Fig. 9). Effect size estimates were concordant too, although to a lesser extent, which can be explained by the different underlying models with different units (Gaussian for pseudobulk versus Poisson model for read counts from individual cells; Pearson’s correlation ranges from 0.35 to 0.71; Extended Data Fig. 10). The low correlation coefficients are probably a result of differences in effect size units between methods: TensorQTL reports effect sizes in standard deviations of mean expression across donors, while SAIGE-QTL reports effect sizes in raw single-cell read counts.

To obtain gene-level *P* values for TensorQTL, we used the beta approximation method described in the FastQTL paper^32^, using 10,000 permutations as recommended by the authors (Methods). For SAIGE-QTL, we used the ACAT-V test to combine correlated *P* values using the Cauchy combination^27,35^, as described above. In both cases, we further corrected for multiple testing across all genes tested (for each cell type) using the *q*-value method^36^, reporting results at FDR < 5%. For both sets of results, we confirmed a clear link between the number of cells available for each cell type and the number of eQTLs detected (Pearson’s correlation: 0.96 and 0.97 for SAIGE-QTL and TensorQTL, respectively; Fig. 1b), with SAIGE-QTL detecting 48.8% more eGenes than TensorQTL (17,218 versus 11,569 eGenes across all 14 cell types) (Supplementary Table 7a). Improved power of SAIGE-QTL compared to TensorQTL was confirmed by comparing the chi-squared statistics of the most significant genetic variants with nominal *P* < 5 × 10^−8^ in eGenes across the two methods, which ranged between 3.8% and 19.2% higher for SAIGE-QTL across all 14 cell types (Fig. 1c). If the two methods identified different top variants, the ones identified by TensorQTL were included in the comparison. As the ACAT-V test can provide slightly more significant *P* values than beta permutation (Extended Data Fig. 2a), we also applied ACAT-V on nominal *P* values from TensorQTL (Extended Data Fig. 2b). We observed that TensorQTL with ACAT-V detected 10.4% more eGenes than with beta permutation (12,777 versus 11,569) (Supplementary Table 7b). Other analyses in this paper used results from TensorQTL with beta permutation, which the original TensorQTL program used.

To further evaluate the power of our method, we assessed the effect of the number of cells available for association. Considering the most abundant OneK1K cell type, CD4_NC_, we considered different levels of down-sampling, considering subsets of 1%, 5%, 10%, 20% and 50% of all cells (keeping the cell-to-donor ratio intact; Methods). In all cases, we ran SAIGE-QTL after selecting all genes expressed in at least 1% of all cells considered (post subsetting). Additionally, we generated pseudobulk counts (by considering the mean expression level across cells for each individual, gene and cell type) for each of the five cell subsets and also ran TensorQTL for the same genes. As expected, the number of eGenes detected decreased as the number of cells considered decreased, and SAIGE-QTL consistently identified more significant eGenes than TensorQTL (7.5% more on average; Fig. 1d, left). We also assessed the effect of the number of individuals available for association on the power. Considering the CD4_NC_ cell type again, we used different subsets of 5%, 10%, 20% and 50% of all samples to evaluate the power of our method compared to TensorQTL. Similarly, the number of eGenes detected decreased as the number of samples considered decreased; SAIGE-QTL identified more significant eGenes than TensorQTL (8% more on average; Fig. 1d, right). Similar improvements of SAIGE over TensorQTL persist when the analyses were limited to using those genetic variants that are tested in all sample subsets (Supplementary Fig. 1)

**Fig. 2:**
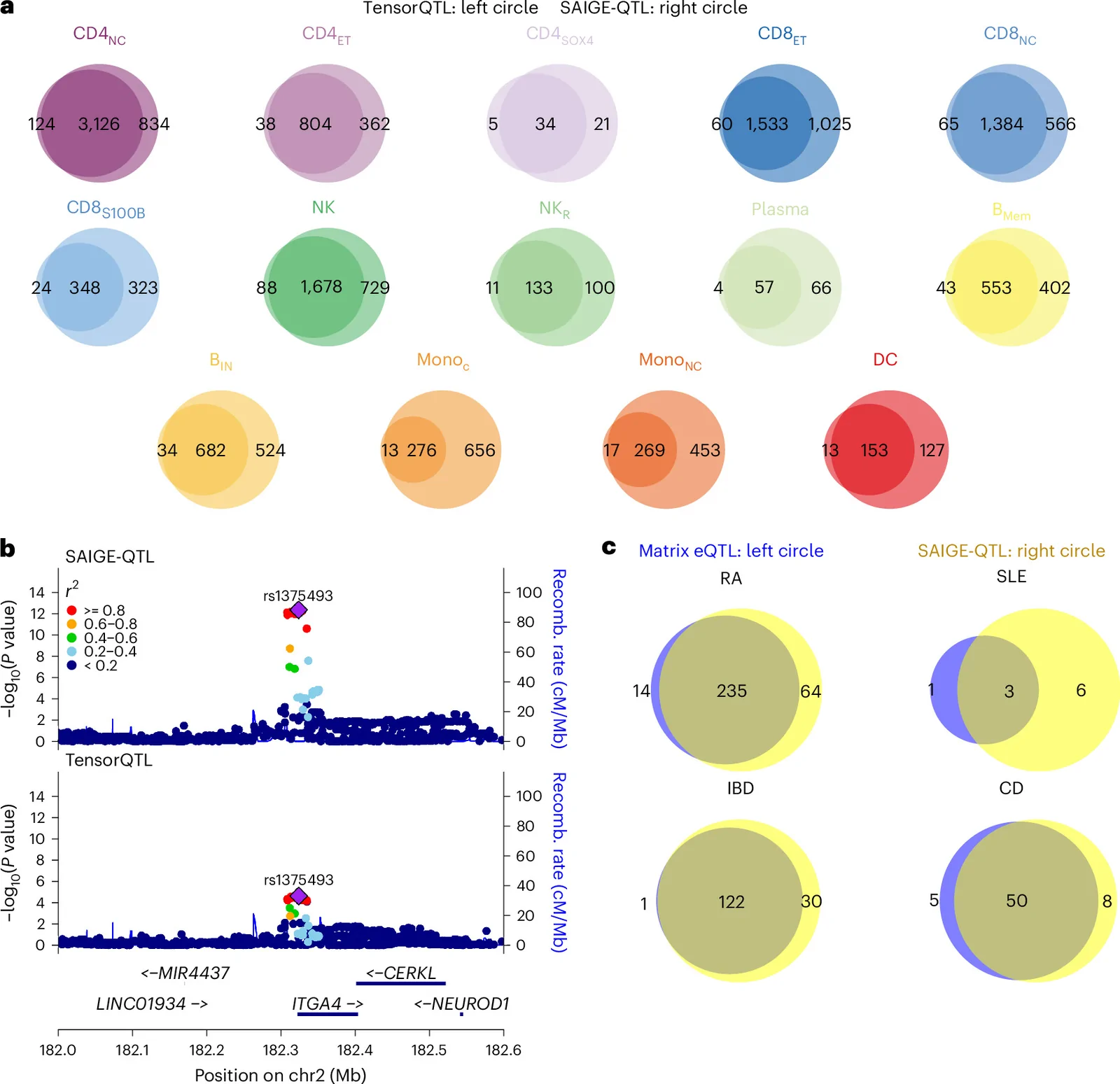
SAIGE-QTL single-cell eQTL mapping identifies more eQTLs than pseudobulk methods. **a**, Venn diagrams representing the overlap of significant eGenes (FDR < 5%, ACAT-V gene-level test) identified by SAIGE-QTL and TensorQTL across all 14 cell types. **b**, Example of disease-specific eQTL detected by SAIGE-QTL and not by TensorQTL (*ITGA4* locus, monocyte-specific eQTL; Extended Data Fig. 2). *P* values are from two-sided single-variant score tests (SAIGE-QTL) and Wald tests (TensorQTL); no multiple-comparison correction is applied within this single-locus view. **c**, Among eGenes identified by SAIGE-QTL and TensorQTL across 14 cell types, gene–cell-type pairs identified by the SMR framework to be associated with four autoimmune disease risks (two-sided SMR test, Bonferroni-corrected at *P* < 0.05/*M* across *M* genes tested per cell type; Methods). CD, Crohn’s disease; RA, rheumatoid arthritis; SLE, systemic lupus erythematosus.

Reassuringly, we retained a large overlap between the sets of eGenes (at FDR < 5%) identified across the two methods, with on average 94% of eGenes identified by TensorQTL also found by SAIGE-QTL (across 14 cell types; range, 87–96%; Fig. 2a). As an example of an eQTL found using SAIGE-QTL and missed by TensorQTL, we identified an eQTL (rs1375493) for the inflammatory bowel disease (IBD)-associated *ITGA4* locus, which is a well-known regulatory signal, and we were able to recapitulate the monocyte-specificity of the eQTL, which has been previously shown^4^ (Fig. 2b and Supplementary Fig. 2). Similar to what was done in the original OneK1K study, we next performed five rounds of conditional association analysis (conditioning on previously significant variants to identify additional independent signals), which identified 4,647 additional eQTLs across the 14 cell types in OneK1K (for 2,435 genes; Supplementary Fig. 3).

### SAIGE-QTL identified additional immune disease-associated loci mediated by gene expression

We then used a Mendelian randomization approach to identify loci contributing to autoimmune disease through gene expression changes in any of the 14 cell types. We used the summary-data-based Mendelian randomization (SMR) tool (Methods) to conduct the analysis using eQTL results from SAIGE-QTL and the original Matrix eQTL^34^ and GWAS summary statistics for four autoimmune diseases: Crohn’s disease^37^, IBD^37^, rheumatoid arthritis^38^ and systemic lupus erythematosus (SLE)^39^. We found that the SMR *P* values are very concordant when using results from the two methods, with results from SAIGE-QTL identifying more SMR associations than Matrix QTL across all four diseases (58 versus 55 for Crohn’s disease; 152 versus 123 for IBD; 299 versus 249 for rheumatoid arthritis; 9 versus 4 for SLE; Supplementary Table 8), with on average 90% gene–cell-type combinations using eQTL results from Matrix eQTL also identified when using eQTL results from SAIGE-QTL (Fig. 2c). In addition, using results from SAIGE-QTL, more unique loci were detected to contribute to the disease of interest through gene expression change in one or more cell types (17 versus 14 for Crohn’s disease; 34 versus 28 for IBD; 77 versus 63 for rheumatoid arthritis; 5 versus 4 for SLE; Supplementary Fig. 4). Notably, several loci identified using eQTL results from SAIGE-QTL but missed by using results from Matrix eQTL act through changes in expression of genes that are known to be implicated in the autoimmune diseases. For example, the locus at 9q21 was shown to affect the risk of Crohn’s disease and IBD through changes in *CARD9* expression in monocytes (classical monocytes, Mono_C_), where previous studies suggested the dysfunction of *CARD9* may contribute to the pathogenesis of IBD^40,41^. The locus 20q13 was identified to causally contribute to rheumatoid arthritis through the gene expression change of *CD40* in plasma, and the CD40 pathway has long been implicated in different studies for RA^42^, which is a promising treatment target^43^.

#### SAIGE-QTL enables genome-wide scan for identifying *trans*-eQTLs

**Fig. 3:**
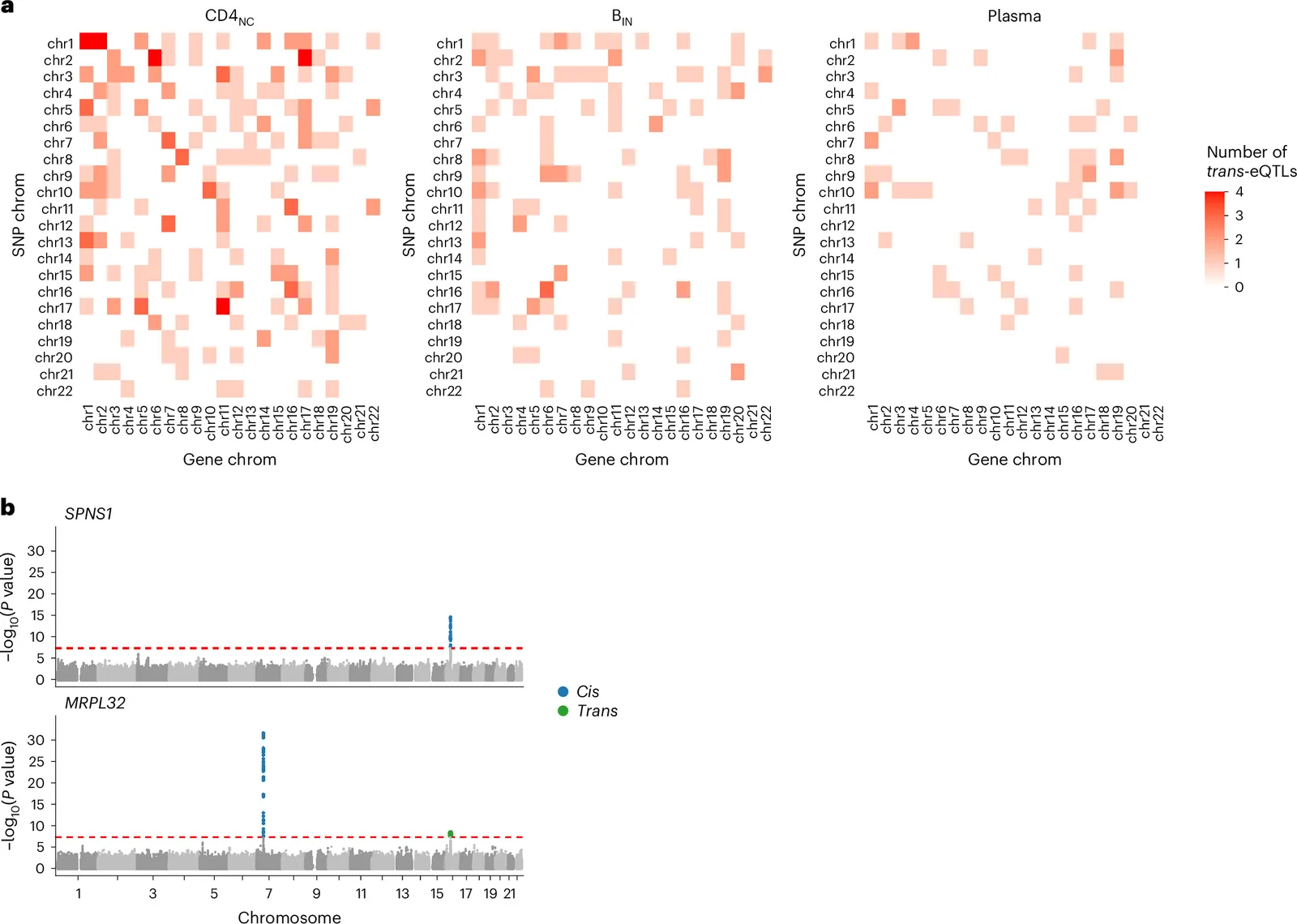
Computational efficiency of SAIGE-QTL allows for genome-wide scans to identify *trans*-eQTLs. **a**, Overview of *trans-*effects (heatmap of chromosome-by-chromosome relationships) in three representative cell types. *P* values are from two-sided single-variant score tests; *P* < 5 × 10^−8^ is the standard genome-wide significance threshold, correcting for the number of variants tested. **b**, Example of a variant that has a *cis-*eQTL effect (blue) on *SPNS1* (top) and also a *trans*-eQTL effect (green) on a different gene (*MRPL32*, bottom) in CD4_NC_. Shown are Manhattan plots for each of the two genes; *P* values are from two-sided single-variant score tests, with the same *P* < 5 × 10^−8^ significance threshold (dashed line) as in **a**. Gray and light-gray points alternate by chromosome for visual clarity only and carry no statistical meaning.

Scalable and fast, SAIGE-QTL enables genome-wide scans of genetic effects on single-cell expression, effectively allowing testing for *trans*-eQTLs (in which the genetic variant tested is far away or even on a different chromosome than the target gene). We mapped *trans*-eQTLs for the three representative cell types selected above (CD4_NC_, B_IN_, plasma). In total, we identify 413 *trans*-eQTLs for 390 genes across the three cell types (at *P* < 5 × 10^−8^, MAF ≥ 10%; Fig. 3a; results for *P* < 5 × 10^−6^ are reported in Methods and Supplementary Fig. 5). As an example, rs3924376 on chromosome 16 is a *cis*-eQTL in CD4_NC_ for *SPNS1*, a gene involved in mitochondrial homeostasis^44^, and a *trans*-eQTL for *MRPL32*, a chromosome 7 gene encoding mitochondrial ribosomal protein L32 (Fig. 3b).

#### Rare variant eQTL signals identified by SAIGE-QTL

**Fig. 4:**
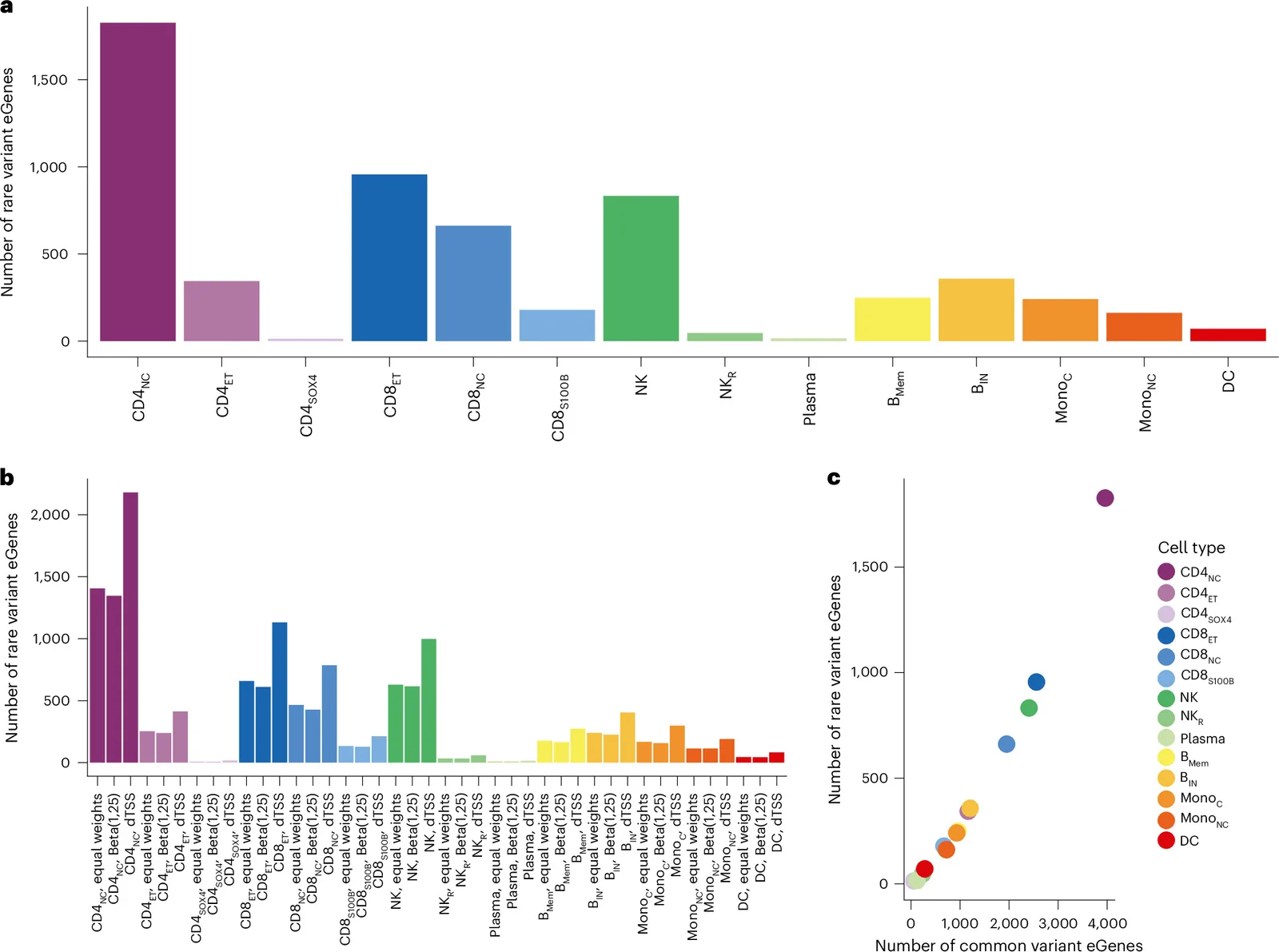
Rare variant eQTLs identified in OneK1K. **a**, Overview of rare variant signals (MAF ≤ 5%) across 14 cell types (all *cis* variants collapsed; Cauchy/ACAT-O combination of Burden, SKAT and ACAT-V tests across the three weighting schemes in **b**). Bars represent the number of significant eGenes (FDR < 5%, *q*-value-corrected). **b**, Effect of different weighting strategies in the set-based tests across all cell types (FDR < 5%, *q*-value-corrected separately per weighting scheme). Compared methods are equal weights, Beta(1,25) distribution and weight based on the dTSS. **c**, Scatterplot of the number of significant eGenes with common variant signals (*x* axis) and rare variant signals (*y* axis) across the 14 cell types.

Finally, we tested for the effects of rare variants on single-cell expression across all 14 cell types. In total, 6,779,365 rare SNPs (MAF ≤ 5%) could be called from the SNP array data from the OneK1K paper^7^. For each gene expressed in at least 10% of donors for a given cell type, we considered all rare variants within a *cis*-window up and downstream of the gene body (±1 Mb). We performed set-based tests (considering all variants in the window at once), including Burden, SKAT and ACAT-V. Overall, we identified 5,541 rare variant (MAF ≤ 5%) eGenes across all 14 cell types (2,317 unique genes; Fig. 4a and Supplementary Fig. 6). Of these, 483 (21%) were independent of common eQTL signals from the same genes, which remained significant (FDR < 0.05) after conditioning on all independent significant common eQTLs (Supplementary Fig. 7). We also ran the set-based tests using weights based on the dTSS (Methods), finding on average 68% (range, 50–89% across cell types) more significant effect compared to using equal weights, and 77% (50–112%) compared to the traditional weighting of variants following a Beta(MAF, 1,25) distribution (Fig. 4b). Compared to the common variants, we observe about one-third of the number of eGenes identified (mean, 0.33; minimum, 0.26; maximum, 0.49) across different cell types, with a very strong correlation owing to the number of cells available for each cell type (Fig. 4c).

#### Performance of SAIGE-QTL compared to existing single-cell QTL methods

We compared SAIGE-QTL with two methods that model single-cell read counts directly: glmer (a Poisson mixed model implemented in the lme4 R package)^45^ previously used^16^, and the association test implemented in CellRegMap (CellRegMap-association)^17^, which uses a linear mixed model. glmer and SAIGE-QTL both model scRNA-seq read counts using the same Poisson mixed model, but SAIGE-QTL uses a Score test^46^, whereas glmer uses a Wald test^47^ for association tests. In a benchmark of 100 genes in B_IN_ cells, SAIGE-QTL reduced computation time 244-fold relative to glmer while producing highly concordant results (Pearson *R*^2^ = 0.991 for −log_10_(*P* values) and 0.998 for effect estimates; Supplementary Table 9 and Supplementary Fig. 8). SAIGE-QTL was also highly concordant with CellRegMap-association (Pearson *R*^2^ = 0.982), which is designed primarily for dynamic eQTL mapping rather than optimized association testing and is therefore not directly comparable in computational performance. SAIGE-QTL additionally supports set-based tests for rare variant eQTL mapping^17,46,47^ (Methods and Supplementary Note).

#### Performance of SAIGE-QTL compared to existing pseudobulk methods

We further compared SAIGE-QTL with two pseudobulk methods, TensorQTL (linear regression) and jaxQTL^48^ (negative binomial regression on pseudobulk counts), evaluating computational cost, type I error and power (Methods). In B_IN_ cells, TensorQTL and jaxQTL were 4.4-fold and 4.0-fold faster than SAIGE-QTL, respectively, but SAIGE-QTL used less memory than both (Supplementary Table 9). Type I error rates were well controlled for SAIGE-QTL and TensorQTL across significance thresholds down to 5 × 10^−5^, whereas jaxQTL showed modest inflation that increased at more stringent thresholds (Supplementary Table 10 and Supplementary Fig. 9). SAIGE-QTL had the highest empirical power across all simulated scenarios, with the advantage over both pseudobulk methods most pronounced in less abundant cell types (Supplementary Table 11 and Supplementary Fig. 10). This power advantage reflects the ability of SAIGE-QTL to model donor-level and cell-level covariates directly at the single-cell level, whereas pseudobulk methods must aggregate covariates and expression to the donor level, discarding information about within-donor cell-to-cell variability and unequal cell counts per donor; simulations confirmed that the power gap widens as cell counts become more uneven and cell-level covariate effects increase (Supplementary Fig. 11 and Supplementary Note).

### Replication of the improved performance of SAIGE-QTL over the pseudobulk approach in two additional datasets

To further assess the performance of SAIGE-QTL across diverse real-world scRNA-seq datasets, we conducted *cis-*eQTL mapping in two additional datasets. First, the California Lupus Epidemiology Study contains scRNA-seq data from 91 European ancestry individuals, in which 21 are SLE cases and 70 are healthy controls^15,49^. Using SAIGE-QTL and TensorQTL, we mapped *cis*-eQTLs in B cells (89 donors) and natural killer cells (91 donors), respectively, across all SNPs with MAF > 5% of genes expressed in at least 10% of donors, adjusting for age, sex, processing batch, SLE disease status, the top six genotype principal components and two cell-type-specific expression PEER factors. For both cell types, SAIGE-QTL was able to identify more eGenes (FDR < 0.05) than TensorQTL (88 versus 83 for B cells and 84 versus 83 for natural killer cells), and 83.1% and 75.9% of eGenes detected through TensorQTL were also found using SAIGE-QTL (Supplementary Fig. 12a and Supplementary Table 12). Strikingly, 89.8% of eGenes in B cells and 94.0% in natural killer cells identified in this study were also detected in the equivalent cell types in OneK1K (B_IN_ cells and natural killer cells, respectively).

Next, we applied SAIGE-QTL to a scRNA-seq dataset containing induced pluripotent stem cells reprogrammed from fibroblasts of patients with primary open-angle glaucoma. The dataset contains 110 unique induced pluripotent stem cell lines, categorized into six distinct cell types: cone, interneuron, lens, retinal ganglion cell, retinal progenitor cell and retinal pigmented epithelial cell, with 101–191,667 cells from 52–110 donors per cell type, as previously described^12^. *Cis*-eQTL mapping was conducted using SAIGE-QTL and TensorQTL using sex, age, primary open-angle glaucoma disease status and the top six genotype principal components as covariates. Across all six cell types, SAIGE-QTL identified 46–99% more eGenes than TensorQTL (15 versus 7 for cone; 529 versus 6 for interneuron; 30 versus 1 for lens; 237 versus 105 for retinal ganglion cell; 391 versus 290 for retinal progenitor cell; 4 versus 0 for retinal pigmented epithelial cell), with 71.4–100% of the eGenes detected by TensorQTL also replicated by SAIGE-QTL (Supplementary Fig. 12b and Supplementary Table 13). These findings further confirmed the improved performance of SAIGE-QTL over the pseudobulk approach across different scRNA-seq datasets.

## Discussion

Here we introduce SAIGE-QTL, an efficient open-source tool implementing a Poisson mixed model. This tool directly models single-cell profiles as discrete counts while accounting for individual effects and relatedness between individuals, and is scalable and efficient, which allows for fast, genome-wide scans. Additionally, compared to existing methods, SAIGE-QTL implements not only single-variant tests but also set-based tests, which are better powered than single-variant tests for low-frequency variants.

We demonstrated that SAIGE-QTL is well calibrated for both single-variant tests for common variants and set-based tests for rare and less frequent variants. As evaluated using the OneK1K dataset^7^, we have shown that eQTL mapping at the cellular level using SAIGE-QTL identified 48.4% more eGenes than the previous analyses using the pseudobulk approach. For about one-third of the number of eGenes identified through common variant tests, we identified rare variant (MAF ≤ 5%) eGenes through set-based tests, of which 21% were independent of common eQTL signals from the same genes. In addition, we identified 413 *trans*-eQTLs for 390 genes in three representative cell types (CD4_NC_, B_IN_, plasma).

In addition to methodological advances, applying SAIGE-QTL to real data enabled several biological insights. Integration with GWAS by Mendelian randomization identified additional disease-relevant loci acting through cell-type-specific expression, and the scalable framework enabled genome-wide *trans*-eQTL discovery. In addition, SAIGE-QTL’s rare variant burden and SKAT tests have been applied to whole-genome sequencing data in the TenK10K Phase 1 study, enabling in-depth characterization of rare variant effects on single-cell expression^50^.

SAIGE-QTL has existing limitations. A Poisson model may not fit genes with substantial overdispersion as well as a negative binomial model, and gene-specific features such as expression sparsity can influence the relative performance of single-cell and pseudobulk analyses, meaning that SAIGE-QTL does not outperform TensorQTL for every gene (Extended Data Fig. 9). The current implementation does not test genotype-by-context interactions, which are important for mapping dynamic eQTLs^16^. Finally, several approaches used by SAIGE-QTL to enhance computational efficiency are not directly applicable to random-slope or spatial-correlation models. Extending the framework to these settings will further broaden genetic analysis of emerging single-cell and spatial transcriptomic resources.

## Methods

SAIGE-QTL builds on the previously proposed SAIGE^21^ and SAIGE-GENE^22,23^ tools, with some key additions: (1) the phenotype is modeled to follow a Poisson distribution, which is better suited for the single-cell expression count data; (2) random effect accounting for multiple cells per individual (in addition to the random effect accounting for relatedness between individuals); (3) improved computational efficiency for fitting Poisson mixed models with a random intercept; (4) ACAT-V^27^ test in addition to the Burden and SKAT for set-based tests for less frequent or rare genetic variants with multiple user-specified weights; (5) the Cauchy method (ACAT-O^27^) for gene-level *P* value combination to identify *cis*-eGenes; and (6) *cis*-window file as an input file to the open-access software to facilitate *cis* testing, and multi-gene implementation for genome-wide (*trans*) tests. The key steps are summarized below, and details of our method can be found in Supplementary Note.

### SAIGE-QTL

In a scRNA-seq study that sequenced *N* cells from *n* individuals, a Poisson generalized linear mixed model is used to model the read counts from the *j*th cell of the *i*th individual. Here *y*_*ij*_ is distributed as (denoted by ~) a Poisson distribution, ${y}_{{ij}}\, \sim {{\rm{Poisson}}}({\mu }_{{ij}})$. Let *X*_*cij*_ denote *p*_c_ cell-level covariates, such as cell states and cell-level expression PEER factors, including the intercept for the *j*th cell of the *i*th individual, and *X*_d*i*_ denote *p*_d_ individual (donor)-level covariates, such as age, gender and genotype principal components for the *i*th individual. Let *G*_*i*_ ∈{0,1,2} represent minor allele counts (MACs) for the genetic variant of interest. The Poisson mixed model can be written as ${\mu }_{{ij}}=\exp \left({\eta }_{{ij}}\right)$ and ${\eta }_{{ij}}={X}_{{\rm{d}\it{i}}}{\alpha }_{\rm{d}}+{X}_{{\rm{c}\it{ij}}}{\alpha }_{\rm{c}}+{G}_{i}\beta +{b}_{i}\,$, where *β*, *α*_d_ and *α*_c_ are the regression coefficients of the genotype *G*_*i*_ and the covariates ${X}_{\rm{d}\it{i}}$ and ${X}_{{\rm{c}\it{ij}}}$, respectively. Formulating the model using matrices and vectors, let *Z* denote the *N* × *n* design matrix with 1s and 0s to indicate which individual each cell belongs to. The model can also be written as $\eta ={X}_{\rm{c}}{\alpha }_{\rm{c}}+Z{X}_{\rm{d}}{\alpha }_{\rm{d}}+{ZG}\beta +Z{b}_{\rm{d}}$, where *η* is the *N* × 1 vector of *η*_*ij*_, *X*_c_ is the *N* × (*p*_c_ + 1) matrix containing the cell-level covariates including the intercept, *X*_d_ is the *n* × *p*_d_ matrix containing individual (donor)-level covariates and the random effect *b* = *Zb*_d_, where *b*_d_ is the *n* × 1 vector of *b*_*i*_ and is assumed to follow multivariate normal distribution ${b}_{\rm{d}} \sim {N}\left(0,{\sum }_{k=1}^{K}{\tau }_{k}{\varPsi }_{k}\right)$ with *Ψ*_*k*_ being the known *K* variance–covariance matrices, which can include the identity matrix to account for intra-individual variance of read counts across multiple cells and a sparse genetic relationship matrix to account for sample relatedness, if any. We test the null hypothesis of no genetic association: *H*_0_, *β* = 0 versus *H*_1_, *β* ≠ 0.

#### Estimating the variance component and other model parameters (step 1)

Similar to SAIGE^21^ and GMMAT^51^, SAIGE-QTL fit the null model $\eta ={X}_{\rm{c}}{\alpha }_{\rm{c}}+Z{X}_{\rm{d}}{\alpha }_{\rm{d}}+Z{b}_{\rm{d}}$ in step 1. Model parameters are estimated iteratively using the PQL^52^ method and the AI-REML algorithm^53^. At iteration *q*, let $\left({{\hat{\tau }}_{k}^{(q)}}\,,\,{{\hat{\alpha }}_{d}^{(q)}},\,{{\hat{\alpha }}_{c}^{(q)}},\,{{\hat{b}}_{d}^{\left(q\right)}}\right)$ be estimated as $({\hat{\tau }}_{k},\,{\hat{\alpha }}_{\rm{d}},\,{\hat{\alpha }}_{\rm{c}},\,{\hat{b}}_{\rm{d}})$, let ${{\hat{\mu }}_{{ij}}^{(q)}}$ be the estimated mean of *y*_*ij*_, ${\hat{W}}^{(q)}={{\rm{diag}}}\left({{\hat{\mu }}_{{ij}}^{(q)}}\right)$ and let ${\hat{\varSigma }}^{(q)}={\left\{{\hat{W}}^{(q)}\right\}}^{-1}$ $+{\sum }_{k=1}^{K}{{\hat{\tau }}_{k}^{(q)}}{Z\varPsi }_{k}{Z}^{T}$ be an *N* × *N* variance matrix of the working vector $\widetilde{y}={X}_{\rm{c}}{{\hat{\alpha }}_{\rm{c}}^{(q)}}+Z{X}_{\rm{d}}{{\hat{\alpha }}_{\rm{d}}^{(q)}}+Z{{\hat{b}}_{\rm{d}}^{(q)}}+\left(y-{\hat{\mu }}^{(q)}\right)/{\hat{\mu }}^{(q)}$.

The SMW formula is used to efficiently compute the product of $${\left\{{\hat{\varSigma }}^{(q)}\right\}}^{-1}$$ and an *N* × 1 vector, *s*, at each iteration of model fitting. The SMW formula exploits the low-rank structure of $${\hat{\varSigma }}^{\left(q\right)}={A}^{-1}+{UCV}$$, where $$A={\hat{W}}^{(q)},U=Z,C={\sum }_{k=1}^{K}{{\hat{\tau }}_{k}^{(q)}}{\varPsi }_{k}$$ and *V* = *Z*^*T*^. This transforms the *N* × *N* matrix inversion problem into solving an *n* × *n* system: $${\left\{{\hat{\varSigma }}^{(q)}\right\}}^{-1}s=$$$$A\left(s-U{\left({C}^{-1}+{VAU}\right)}^{-1}{VAs}\right)$$. This reduces the computational complexity from *O*(*N*^3^) to $$O\left({{\sum }_{k=1}^{K}{{nnz}}_{k}+{n}}^{2}+N\right)$$, where *n* is the number of donors and *nnz*_*k*_ is the number of non-zero elements in the *k*th *n* ×*n* sparse *Ψ*_*k*_. When *K* = 1 with unrelated donors (*Ψ*_1_ = *I*), SMW reduces to closed-form block-wise operations with optimal *O*(*N*) complexity. SAIGE-QTL also implements the PCG^30^ approach for iteratively solving linear systems instead of inverting the *N* × *N* matrices directly. Computing the product of $${\hat{\varSigma }}^{(q)}$$ and an *N* × 1 vector, *s*, requires only consecutive matrix–vector multiplications involving *Z*^*T*^, *Ψ*_*k*_, *Z* and *s*, without pre-computing and storing the *N* × *N* matrix$$\,{Z\varPsi }_{{k}}{Z}^{{T}}.$$ This reduces complexity from *O*(*N*^3^) to $$O({q}_{{{\rm{iter}}}}\left({\sum }_{k=1}^{K}{{nnz}}_{k}+N\right))$$, where *q*_iter_ is the number of PCG iterations.

Both approaches are particularly efficient as *n* ≪ *N*; for example, when millions of cells are generated from hundreds or thousands of donors. SAIGE-QTL defaults to SMW. When *n* is large and SMW incurs high memory usage, the PCG approach can be used for improved scalability.

#### Estimating the variance ratios

A score test statistic for *H*_0_, *β* = 0 is $T={G}^{T}{Z}^{T}\left(Y-\hat{\mu }\right)$, where *G* is the *n* × 1 genotype vector, *Y* is the *N* × 1 phenotype vector with read counts from cells and $\hat{\mu }$ is the *N* × 1 vector with an estimated mean of *Y* under *H*_0_. The variance of *T* is ${\rm{Va}}{\rm{r}}_{{H}_{0}}\left(T\right)={\widetilde{G}}^{T}\hat{P}\,\widetilde{G}$, where $\hat{P}={\hat{\varSigma }}^{-1}-{\hat{\varSigma }}^{-1}X{\left({X}^{{T}}{\hat{\varSigma }}^{-1}X\right)}^{-1}{X}^{{T}}{\hat{\varSigma }}^{-1}$, $\widetilde{G}\,={ZG}-{X}{({X}^{T}\hat{W}X)}^{-1}{X}^{T}\hat{W}{ZG}$ is the covariate-adjusted genotype vector, X is the ${N}\times \,({p}_{\rm{c}}+1\,+\,{p}_{\rm{d}})$ matrix with all covariates including the intercept and $\,{X}=\,\left[{X}_{\rm{c}}Z{X}_{\rm{d}}\right]$. For each genetic variant, given $\hat{P}$, the calculation of ${\rm{Va}}{\rm{r}}_{{H}_{0}}\left(T\right)$ requires $O({N}^{2})$ computation. Similar to SAIGE^21^, BOLT-LMM^54^ and GRAMMAR-Gamma^55^, let ${{\rm{Var}}}_{{H}_{0}}\left(T\right)* ={\widetilde{G}}^{T}\hat{W}\widetilde{G}$, which does not incorporate random effects, and it has been shown previously^21,54–56^ that ${\rm{Va}}{\rm{r}}_{{H}_{0}}(T)/{\rm{Va}}{\rm{r}}_{{H}_{0}}{(T)}^{* }$ is approximately constant for all genetic variants with MAC ≥ 20.

Here we estimate two variance ratios. First, similar to what has been used in the GWAS method fastGWA-GLMM^57^, we use the unadjusted (but mean-centered) genotype data, ${G}_{\rm{c}}\,={Z}(G-\frac{1}{n}({1}^{T}G)1)\,$, where 1 is a column vector of ones of size *n* × 1, to calculate ${\rm{Va}}{\rm{r}}_{{H}_{0}}{({T}_{\rm{c}})}^{*} \,=\,{{G}_{\rm{c}}^{T}}\hat{W}\,{G}_{\rm{c}}$ and then estimate the variance ratio ${\hat{r}}_{\rm{c}}={\rm{Va}}{\rm{r}}_{{H}_{0}}(T)/{\rm{Va}}{\rm{r}}_{{H}_{0}}{({T}_{\rm{c}})}^{* }$. Second, to approximate ${\rm{Va}}{\rm{r}}_{{H}_{0}}(T)$ more accurately, we incorporate the random effects via ${\hat{\varSigma }}^{-1}$ into the denominator to estimate the variance ratio $\hat{r}={\rm{Va}}{\rm{r}}_{{H}_{0}}(T)/{\rm{Va}}{\rm{r}}_{{H}_{0}}{(T)}^{* }$, where ${\rm{Va}}{\rm{r}}_{{H}_{0}}{(T)}^{* }={{\widetilde{G}}_{\rm{d}}^{T}}\,{\hat{\varSigma }}^{-1}{\widetilde{G}}_{\rm{d}}.\hat{r}$ has lower variation than ${\hat{r}}_{\rm{c}}$ as previously demonstrated in SAIGE-GENE^22^. To further improve the computational efficiency, we use the donor-level covariates and intercept-adjusted genotype vector ${\widetilde{G}}_{\rm{d}}\,={ZG}-\,{{ZX}}_{{\rm{d}\it{I}}}$ ${({{X}_{{\rm{d}\it{I}}}}^{T}{Z}^{T}\hat{W}{{ZX}}_{{\rm{d}\it{I}}})}^{-1}$ ${{X}_{{\rm{d}\it{I}}}}^{T}{Z}^{T}\hat{W}{ZG}$ to compute ${\rm{Va}}{\rm{r}}_{{H}_{0}}{(T)}^{* }$ and then estimate $\hat{r}$. With pre-computed ${Z}^{T}{\hat{\varSigma }}^{-1}Z$ and ${Z}^{T}\hat{W}Z$, the computation cost of ${\rm{Va}}{\rm{r}}{(T)}^{* }$ is $O((1\,+\,{p}_{\rm{d}}){n}^{2})$ and ${\rm{Va}}{\rm{r}}{({T}_{\rm{c}})}^{* }$ is *O*(*n*), respectively, instead of *O*(*N*).

We have shown that $\hat{r}$ and ${\hat{r}}_{\rm{c}}$ are approximately constant for all genetic variants (Supplementary Note). To estimate $\hat{r}$ and ${\hat{r}}_{\rm{c}}$, we calculate the variance ratios starting with 30 randomly selected genetic variants at an increment of ten genetic variants until the coefficient of variation is smaller than 0.001 and calculate the mean of the variance ratios as $\hat{r}$ and ${\hat{r}}_{\rm{c}}$, respectively.

#### Score test for single variants and set-based tests for rare variants (step 2)

Using the estimated variance ratio ${\hat{r}}_{\rm{c}}$, the variance-adjusted test statistic can be calculated as ${T}_{\rm{adj}}=\frac{{G}^{T}{Z}^{{T}}(Y-\hat{\mu })}{\sqrt{{\hat{r}}_{\rm{c}}{{G}_{\rm{c}}}^{T}\hat{W}\,{G}_{\rm{c}}}}$, which, under the null hypothesis, has mean of zero and variance unity. For variants with *P* < 0.05, we use $\hat{r}$ to re-calculate ${T}_{\rm{adj}}=\frac{{G}^{T}{Z}^{T}(Y-\hat{\mu })}{\sqrt{{{\hat{r}\widetilde{G}}_{\rm{d}}}^{T}\,{\hat{\varSigma }}^{-1}{\widetilde{G}}_{\rm{d}}}}$. This strategy allows SAIGE-QTL to reduce the computation cost to calculate *T*_adj_ from $O((1\,+\,{p}_{\rm{d}}){n}^{2})$ to *O*(*n*) for ~95% of genetic markers. In addition, it has been previously observed that the asymptotic normality assumption of the score test statistic *T*_adj_ leads to severe type I error inflation for low-frequency and rare variants in the context of logistic mixed models with unbalanced case–control ratios^21–23^ and the modified Poisson mixed model when the event rate is low^56^. SPA^24^ has been used to successfully approximate *T*_adj_ for better-controlled type I error rates. In SAIGE-QTL, we also implement and apply SPA to obtain a better approximation of the distribution of *T*_adj_ to obtain more accurate *P* values (Supplementary Note).

For eQTL analysis of rare variants, owing to the lack of power of single-variant tests, SAIGE-QTL conducts set-based tests, including the Burden, SKAT^26^ and ACAT-V^27^ tests. Test statistics of the Burden and SKAT tests can be constructed based on score statistics from the marginal model for individual variants in the testing set, and test statistics of the ACAT-V^27^ test can be constructed using *P* values from the marginal model for individual variants. Following what has been proposed in SAIGE-GENE+^23^, to reduce data sparsity owing to ultra-rare variants, before testing each variant set, ultra-rare variants with MAC ≤ 20 are collapsed in SAIGE-QTL to a pseudo-variant, and then the pseudo-variant is tested together with all other variants with MAC > 20. In addition, multiple functional annotations and maximum MAF cutoffs are allowed to be incorporated into the set-based tests to improve power, and *P* values are combined using the minimum *P* value or Cauchy combination method^27^.

#### Calculating gene-level *P* values for identifying *cis*-eGenes (step 3)

In step 3, the gene-level *P* value for each gene is calculated by combining the variant-level *P* values of common variants (MAF > 5% is used in the OneK1K study) using the ACAT-V^27^ test based on the Cauchy combination as recently suggested in the linear mixed model method for eQTL mapping, APEX^31^. We assume that there are *M* genetic variants in the *cis*-region, let ${T}_{{{\rm{ACAT}}}}\,=\,{\sum }_{m=1}^{M}{v}_{{m}}\tan \{(0.5-{p}_{{m}})\uppi \}$, where *p*_*m*_ is the *P* value for the *m*th variant, *v*_*m*_ is the weight for the *m*th variant and let $v={\sum }_{m=1}^{M}{v}_{{m}}.$ The *P* value of ACAT is approximated by ${p}_{{{\rm{ACAT}}}}\approx 1/2\,-\,\{\arctan \,({T}_{{{\rm{ACAT}}}}\,/v)\}/\uppi$. It is a computationally efficient approach to combine *P* values, which is robust to the correlation between individual *P* values^27^. It provides highly concordant gene-level *P* values compared to those obtained using the beta approximation method^32,33^ (Extended Data Fig. 2), but is much faster as it does not require any permutations to be performed. It also allows for incorporating variant-level weights; for example, dTSS, to improve the power to detect *cis*-eGenes.

#### OneK1K analysis

We consider the OneK1K dataset as it is available from the original publication^7^. This includes genotypes (from SNP array data) and scRNA-seq from 14 different immune cell types for 1,267,758 peripheral blood mononuclear cells and 982 individuals of European ancestry from the Tasmanian Ophthalmic Biobank in Tasmania, Australia. For eQTL mapping with both SAIGE-QTL and TensorQTL, we considered the same covariates used for eQTL mapping in the original paper: sex and age of the individuals, six genotype principal components and two PEER factors calculated from gene expression from each cell type separately. Given that the original study used pseudobulk aggregate counts, the PEER factors are defined per individual, not cell, so we kept those values here (that is, all cells for a given individual were assigned the same PEER factor value). As in the original publication^7^, the SCTransform v1 function in the Seurat R package^58^ was used to normalize read counts by total reads and account for batch and mitochondrial gene percentage, while still returning Poisson counts. The corrected read counts were used as input for step 1 in SAIGE-QTL to fit null Poisson mixed models for each gene in different cell types.

For each gene in each cell type, we calculated the gene-level *P* value in step 3 based on all genetic variants within a 1 Mb window around the gene body with MAF > 5%. No weights are incorporated in the Cauchy combinations for a fair comparison with the results using TensorQTL.

#### Data simulation for type I error evaluation

To evaluate false-positive control, we used real OneK1K single-cell read counts with permuted donor genotypes across three representative cell types (CD4_NC_, B_IN_ and plasma) spanning a range of gene-level expression sparsity (Supplementary Tables 1 and 4). For each gene, we fit the null model on real counts (step 1), then permuted donor identities before single-variant (step 2a) and set-based (step 2b) association testing on chromosome 1, repeating permutations to obtain sufficient tests for evaluating empirical type I error across a range of significance thresholds (Supplementary Tables 5 and 6). The set tests were repeated ten times with permuted individual IDs, and the quantile–quantile plots are shown in Extended Data Fig. 6. Full simulation design and results are in Supplementary Note (Calibration of SAIGE-QTL under permutation).

#### Data simulation for comparing SAIGE-QTL to pseudobulk eQTL methods

To compare calibration and power against pseudobulk methods (TensorQTL, jaxQTL), we simulated single-cell gene expression using a Poisson mixed-effects model with donor-level random intercepts, based on 2,100 linkage disequilibrium-pruned chromosome 22 variants (MAF > 0.05) from OneK1K, for three representative cell types (CD4_NC_, B_IN_ and plasma). Type I error was evaluated under the null hypothesis, and we considered even and uneven per-donor cell-count designs and four levels of cell-level expression sparsity (5%, 25%, 75%, 95% non-zero coverage). Power was evaluated by adding a single causal variant (MAF = 0.2), and for each cell type, power was evaluated across four cell coverage levels corresponding to the empirical 25th, 50th, 75th and 90th percentiles of non-zero expression coverage, reflecting empirical single-cell data in which cell-level coverage is often low and highly right-skewed across cell types (Supplementary Fig. 13). Empirical power and corresponding 95% confidence intervals were computed across 500 replicates. We additionally varied cell-level covariate effect size and per-donor cell-count heterogeneity to assess their impact on the relative power of SAIGE-QTL versus pseudobulk methods. Full simulation design and results are in Supplementary Note (Comparison with pseudobulk eQTL methods: cost, calibration and power).

#### CD4_NC_ cell subsetting

For the subsetting analysis (Fig. 1d), we considered the most abundant cell type from OneK1K: CD4_NC_. For each percentage level of subsetting (1%, 5%, 10%, 20% and 50%), we subsetted the number of total cells to that percentage, while retaining the cell-to-donor ratio for each individual included. Next, we re-mapped *cis*-eQTLs using SAIGE-QTL for each set, maintaining all other settings (covariates included, *cis*-window size). Only genes expressed in at least 1% of the cells were tested, so those numbers decreased for smaller sets. Pseudobulking for TensorQTL was performed post subsetting, by considering the mean expression for each gene, cell type and individual. These values were then logged before eQTL mapping to mirror the approach used in the OneK1K paper^7^.

#### Mendelian randomization analysis

To identify genes whose expression levels are associated with human complex disease risk, we used the SMR^59^ framework and used the top associated *cis*-eQTL of the gene as an instrumental variable. We input the eQTL summary statistics of the OneK1K cohort from SAIGE-QTL and four GWAS summary statistics of autoimmune diseases: Crohn’s disease^37^, IBD^37^, rheumatoid arthritis^38^ and SLE^39^. We also ran the SMR tool using the eQTL summary obtained from Matrix eQTL^34^ for the same 14 cell types as a comparison. A HEIDI (HEterogeneity In Dependent Instruments) test implemented in the SMR framework was further performed to distinguish pleiotropy or causality from linkage. The genes that are not rejected by the HEIDI test indicate that the gene expression levels are associated with the disease risk owing to pleiotropy or causality at one shared genetic variant.

For each cell type, we calculated the *q*-values of each SNP–gene pair (the SNPs within a 1 Mb window around the gene body) and only tested the genes whose top associated *cis*-eQTLs passed the threshold of FDR < 5%, to match what we used throughout the study (the thresholds used for each method and cell type are provided as Supplementary Table 14). Specifically, for each cell type, we considered the weakest eGene still under the threshold of FDR < 5%. Then, we considered the smallest *P* value for that gene across the *cis*-SNPs tested (note that this necessarily results in the number of genes tested being different between SAIGE-QTL and Matrix eQTL). The significance of the SMR test was determined at a *P* value from SMR < 0.05 / *M*, where *M* is the total number of genes tested in each cell type.

#### *Trans*-eQTL mapping

For each of the three representative cell types (CD4_NC_, B_IN_ and plasma), considering the large number of tests and limited power for detecting *trans*-eQTLs with low frequency, we conducted genome-wide single-variant tests for all common variants with MAF > 10%. We define *trans* signals as those outside of a 2 Mb window around the gene on the same chromosome, as well as all inter-chromosome associations. Additionally, we exclude the MHC region on chromosome 6.

We report results at two different significance levels: *P* < 5 × 10^−6^ (Supplementary Fig. 5) and *P* < 5 × 10^−8^ (Fig. 3). At *P* < 5 × 10^−8^, we identified 223 *trans*-eQTLs for CD4_NC_, 124 for B_IN_ and 69 for plasma (211, 120 and 69 unique genes, respectively), for a total of 413 *tran*s-eQTLs for 390 unique genes across all three cell types. At *P* < 5 × 10^−6^, we identified 47,210 *trans*-eQTLs for CD4_NC_, 33,936 for B_IN_ and 13,677 for plasma (12,693, 10,477 and 5,460 unique genes, respectively), for a total of 94,795 *trans*-eQTLs for 13,159 unique genes across all three cell types.

#### Rare variant testing

When testing for the effects of rare variation (MAF ≤ 5%) on single-cell gene expression, we used the Burden, SKAT and ACAT-V tests, and used the Cauchy approach to combine *P* values. We also considered three sets of weights: equal weights, in which each variant is assigned the same weight; weights following a Beta(1,25) distribution, as commonly used in rare variant association tests; and weights based on a variant’s dTSS. For the latter, we followed the procedure outlined by the APEX authors^31^, in which each variant received a weight proportional to ${{\rm{e}}}^{-\gamma \left|d\right|}$, where *d* is the number of base pairs between the variant and the TSS and *γ* = 10^−5^. Additionally, to determine the independence of these signals from common variant effects, we performed conditional analysis, conditioning on all common variant eQTL signals at FDR < 5%, across all five rounds of conditional analysis (Supplementary Fig. 7).

### Inclusion and ethics statement

All collaborators of this study who have fulfilled the criteria for authorship required by Nature Portfolio journals have been included as authors, as their participation was essential for the design and implementation of the study. This work includes findings that are locally relevant, which have been determined in collaboration with local partners. This research was not severely restricted or prohibited in the setting of the researchers, and does not result in stigmatization, incrimination, discrimination or personal risk to participants. Local and regional research relevant to our study was taken into account in citations.

### Reporting summary

Further information on research design is available in the Nature Portfolio Reporting Summary linked to this article.

## Online content

Any methods, additional references, Nature Portfolio reporting summaries, source data, extended data, supplementary information, acknowledgements, peer review information; details of author contributions and competing interests; and statements of data and code availability are available at https://doi.org/10.1038/s41588-026-02798-2.

## Supplementary information

Supplementary Information Supplementary Figs. 1–13 and Note.

Reporting Summary

Peer Review File

Supplementary Tables 1–14 Supplementary Tables 1–14.

## Data Availability

The OneK1K scRNA-seq and genotype data are available in the Gene Expression Omnibus (GSE196830). Summary statistics for the eQTLs in this study can be found on Zenodo at https://zenodo.org/records/10884040 (ref. ^60^). Source data for figures are deposited on Zenodo at https://zenodo.org/records/21987029 (ref. ^61^). Crohn’s disease and IBD GWAS summary statistics^37^ were downloaded from IBD Genetics Consortium website (www.ibdgenetics.org); rheumatoid arthritis GWAS summary statistics^38^ were downloaded from the GWAS Catalog (study ID: GCST90132223) (https://ftp.ebi.ac.uk/pub/databases/gwas/summary_statistics/GCST90132001-GCST90133000/GCST90132223/GCST90132223_buildGRCh37.tsv.gz); SLE GWAS summary statistics^39^ were downloaded from the GWAS Catalog (study ID: GCST90018917) (https://ftp.ebi.ac.uk/pub/databases/gwas/summary_statistics/GCST90018001-GCST90019000/GCST90018917/GCST90018917_buildGRCh37.tsv.gz).

## Code availability

SAIGE-QTL is implemented as an open-source R method available at https://github.com/weizhou0/SAIGEQTL. Code to reproduce all analyses and figures included here can be found at https://github.com/annacuomo/SAIGE_QTL_analyses. Both the SAIGE-QTL software and the analysis code have been deposited in Zenodo^62^.
