# Supplementary material for: Efficient and accurate mixed model association tool for single-cell eQTL analysis
Source: Nat Genet. 2026 Nov 6;58(11):2984–92. doi: 10.1038/s41588-026-02798-2 (PMC13754595; doi:10.1038/s41588-026-02798-2)
Supplement: Supplementary file 1 [file 41588_2026_2798_MOESM1_ESM.pdf]

nature genetics

Supplementary information https://doi.org/10.1038/s41588-026-02798-2

Efficient and accurate mixed model association tool for single-cell eQTL analysis

In the format provided by the authors and unedited

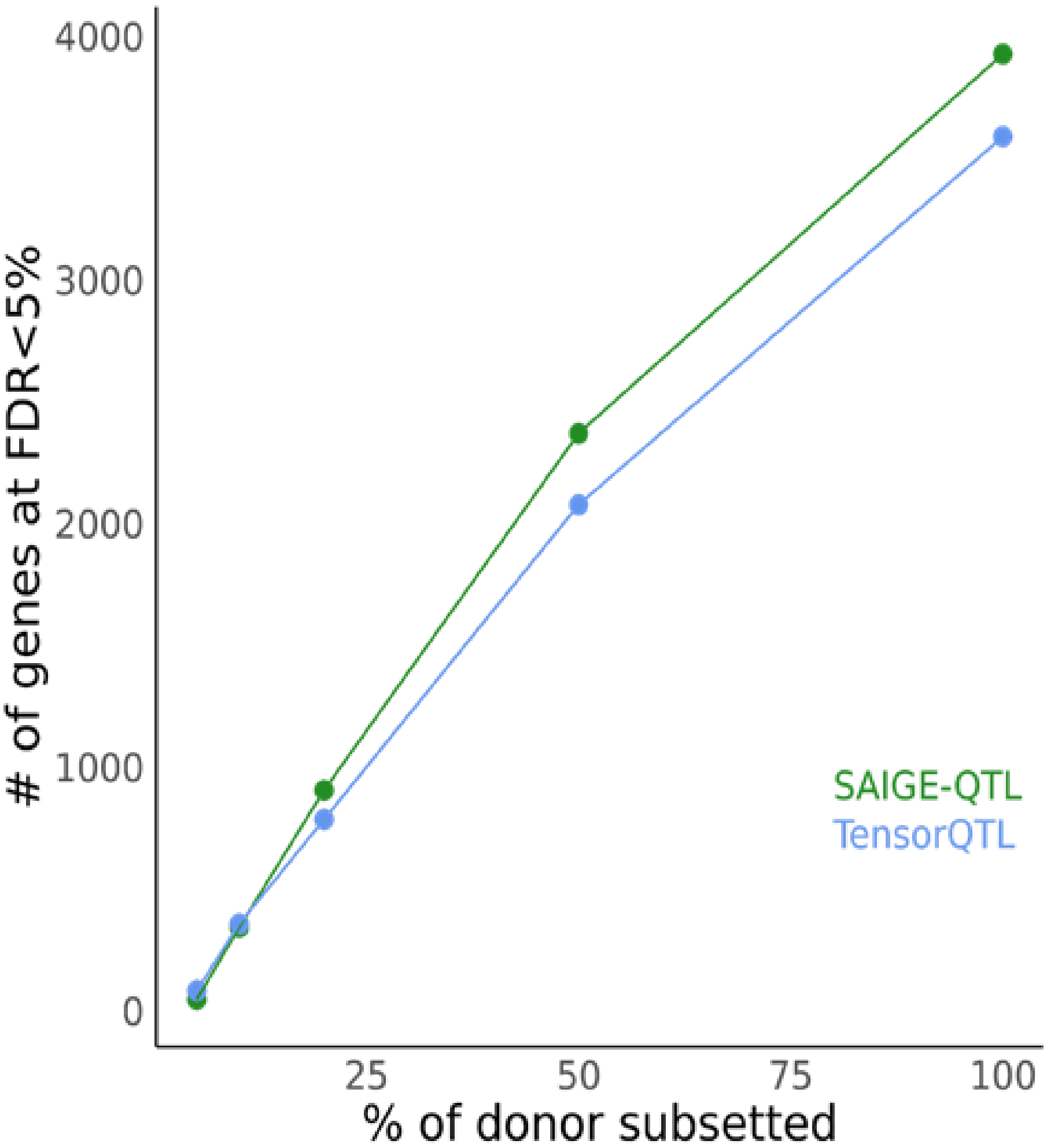

Supplementary Figure 1: Power evaluation of SAIGE-QTL
Scatter plot of power (number of eGenes identified at FDR < 5%, y axis) and percentage of donors subsampled (n = 982 donors total) for the most abundant cell type ($CD4_{NC}$, Methods), when analyses were limited to using genetic variants that are tested in all donor subsets.

Cell type	Beta	SE	P-value
B_IN	0	0.02	9.2e-01
B_Mem	-0.02	0.02	5.3e-01
CD4_ET	0.03	0.02	6.7e-02
CD4_NC	0.01	0.01	4.7e-01
CD4_SOX4	0.09	0.07	2.0e-01
CD8_ET	0.01	0.01	5.4e-01
CD8_NC	0.01	0.01	5.4e-01
CD8_S100B	0.02	0.02	2.6e-01
DC	-0.01	0.04	8.3e-01
Mono_C	-0.2	0.03	3.8e-13
Mono_NC	-0.07	0.02	1.1e-03
NK	0.02	0.01	2.7e-01
NK_R	0.02	0.03	5.7e-01
Plasma	0.01	0.04	8.2e-01

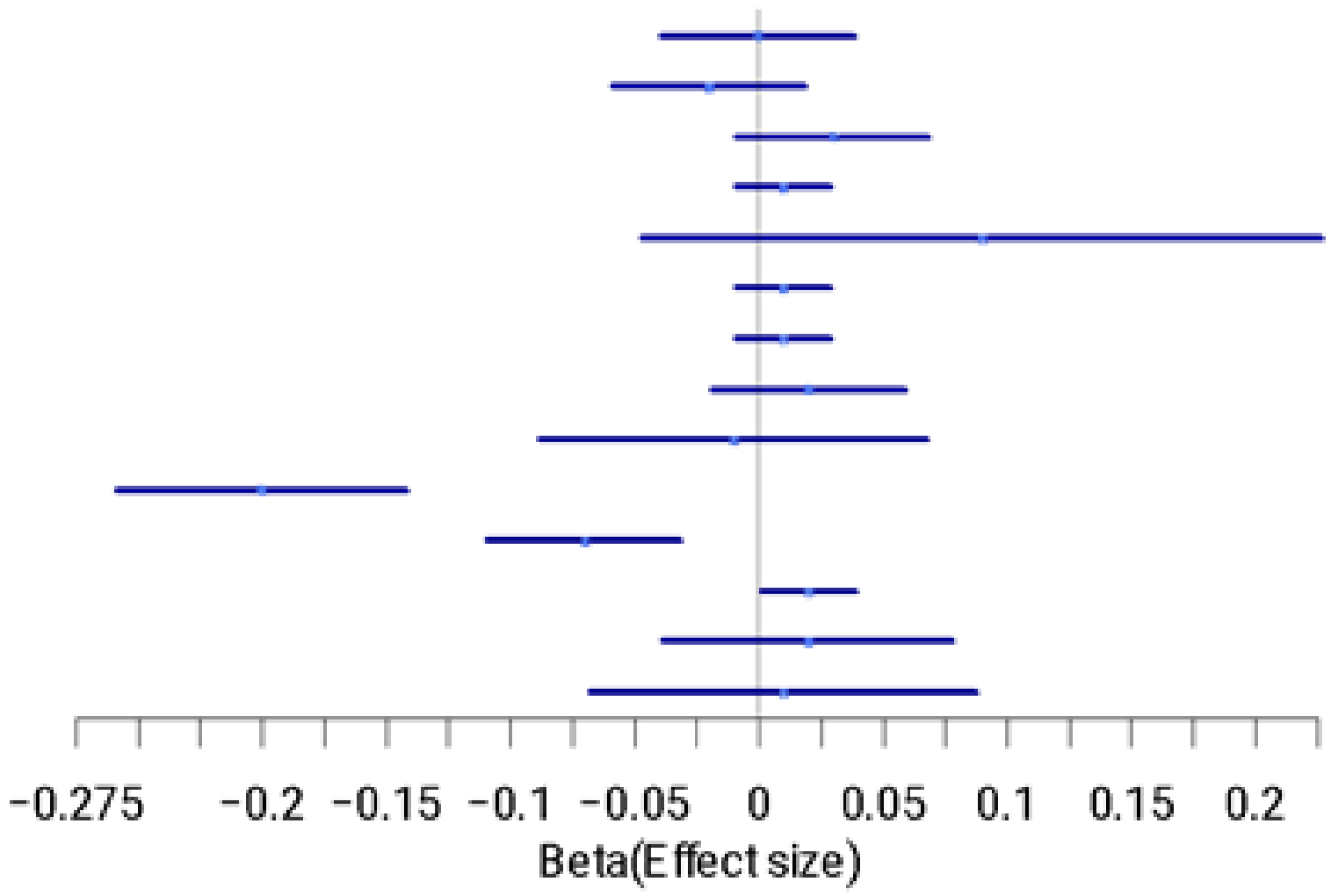

Supplementary Figure 2: Forest plot of effect sizes with 95% confidence intervals of the top cis-eQTL variant rs1375493 in gene *ITGA4* across 14 cell types.
For the significant eQTL identified for *ITGA4* and shown in Figure 2b, this is the forest plot of effect sizes across all cell types in the OneK1K cohort, highlighting that this is a monocyte-specific signal. Beta, standard error (SE) and exact two-sided P values (from SAIGE-QTL single-variant score tests) are shown for each cell type; error bars represent 95% confidence intervals.

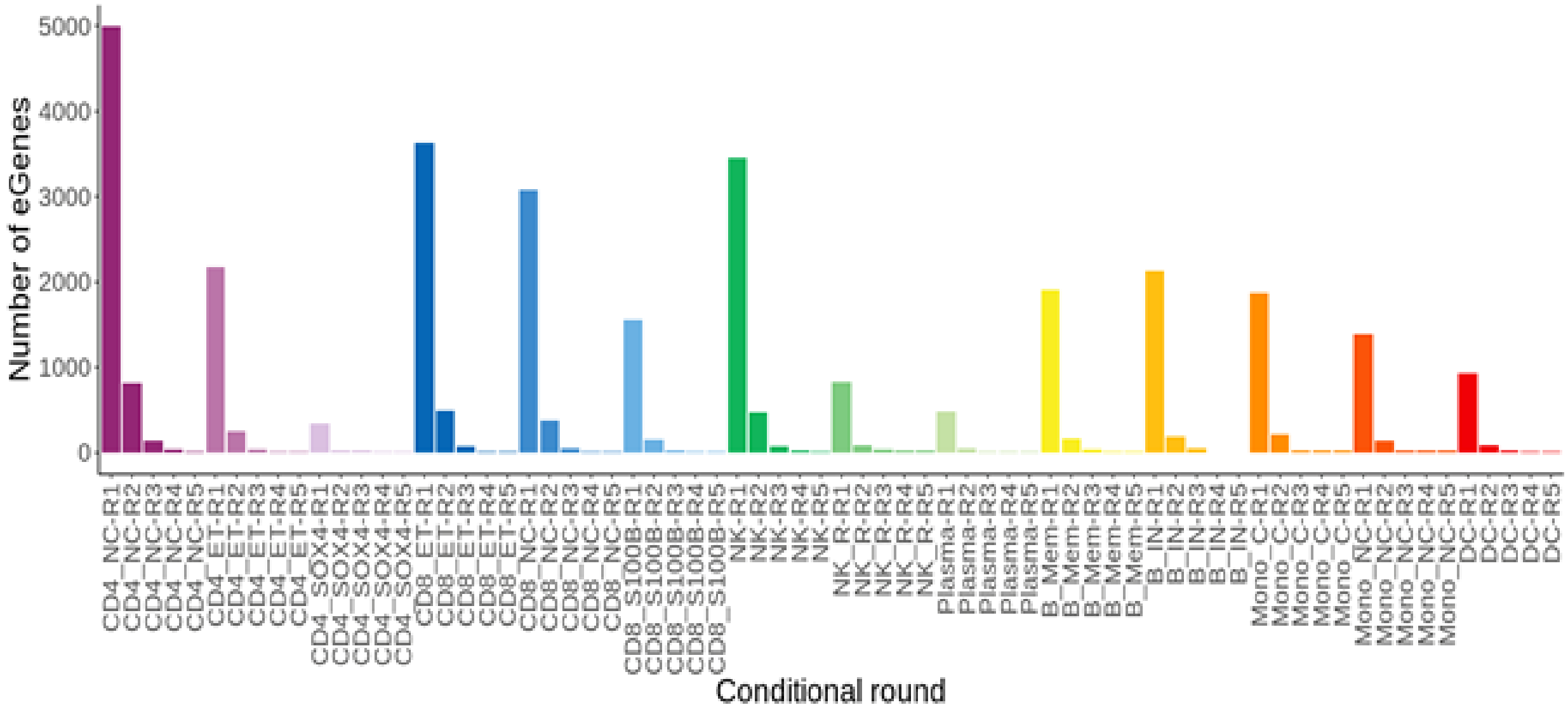

Supplementary Figure 3: Common variants conditional analysis results.
For each of 14 cell types (represented by the different colours), bars represent the number of significant eGenes (FDR<5%) identified for each of 5 rounds of conditional analysis (R1 to R5).

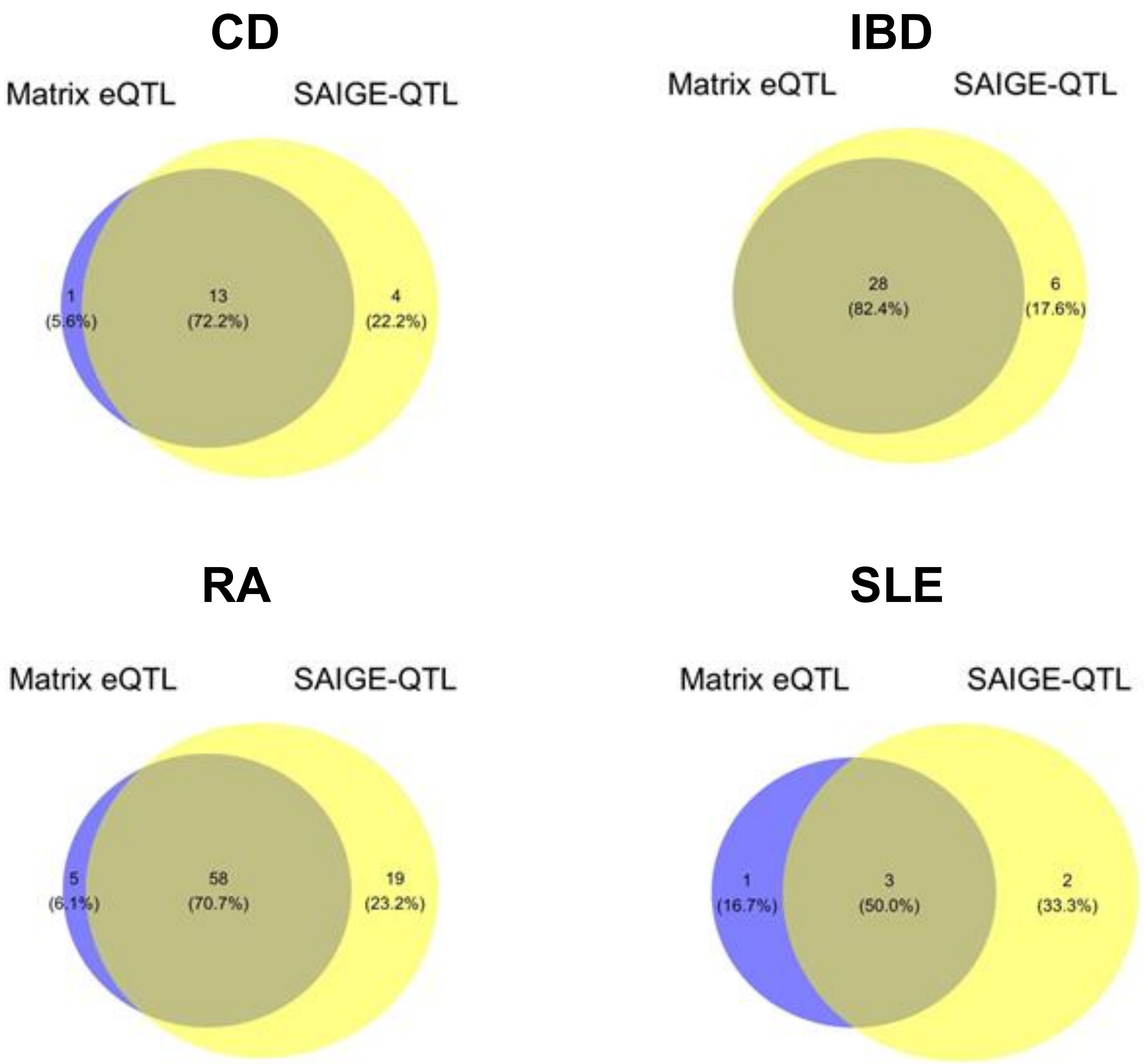

Supplementary Figure 4: Number of loci identified in any cell type by SMR using eQTLs identified by the two methods.

Venn diagrams representing the overlap of significant summary-based Mendelian Randomisation (SMR) results identified based on eQTLs detected using each of the two methods, for the three immune-mediated diseases considered. Similar plots for gene-cell type combinations are shown in the main figure (Fig 2C).

CD: Crohn's disease, IBD: inflammatory bowel disease, RA: rheumatoid arthritis, SLE: systemic lupus erythematosus.

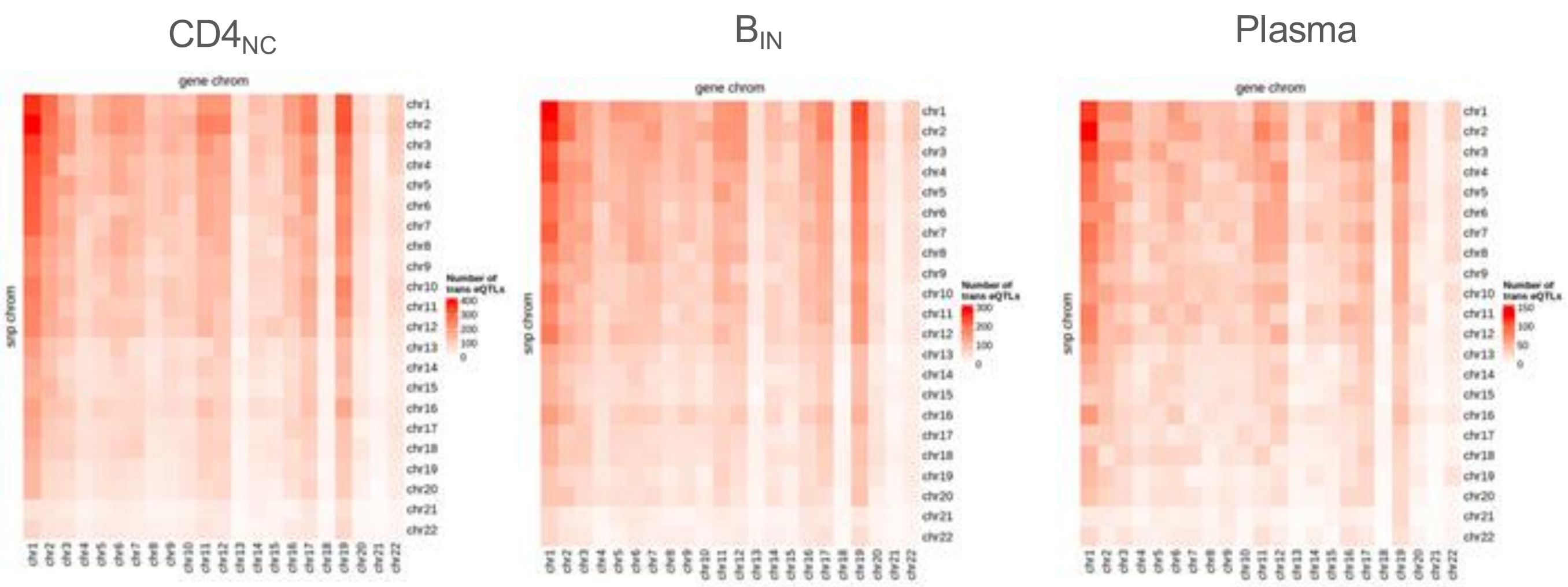

Supplementary Figure 5: *Trans* eQTL effects across three representative cell types.

Same as main Figure 3a, but considering all associations at a nominal significance threshold of two-sided $P < 5 \times 10^{-6}$ (single-variant score tests).

For three representative cell types (Plasma: least abundant; B_{IN} : intermediate, naive and immature B cells; $CD4_{N}$: most abundant, CD4-positive naive and central memory T cells), heatmaps where the colour intensity represents the number of significant independent *trans*-eQTLs (scale bar, right of each panel). Rows correspond to the chromosomal location of the variant, and columns correspond to the chromosomal location of the gene.

a. MAF<= 5% in cis regions of genes in oneK1K (set-based tests)

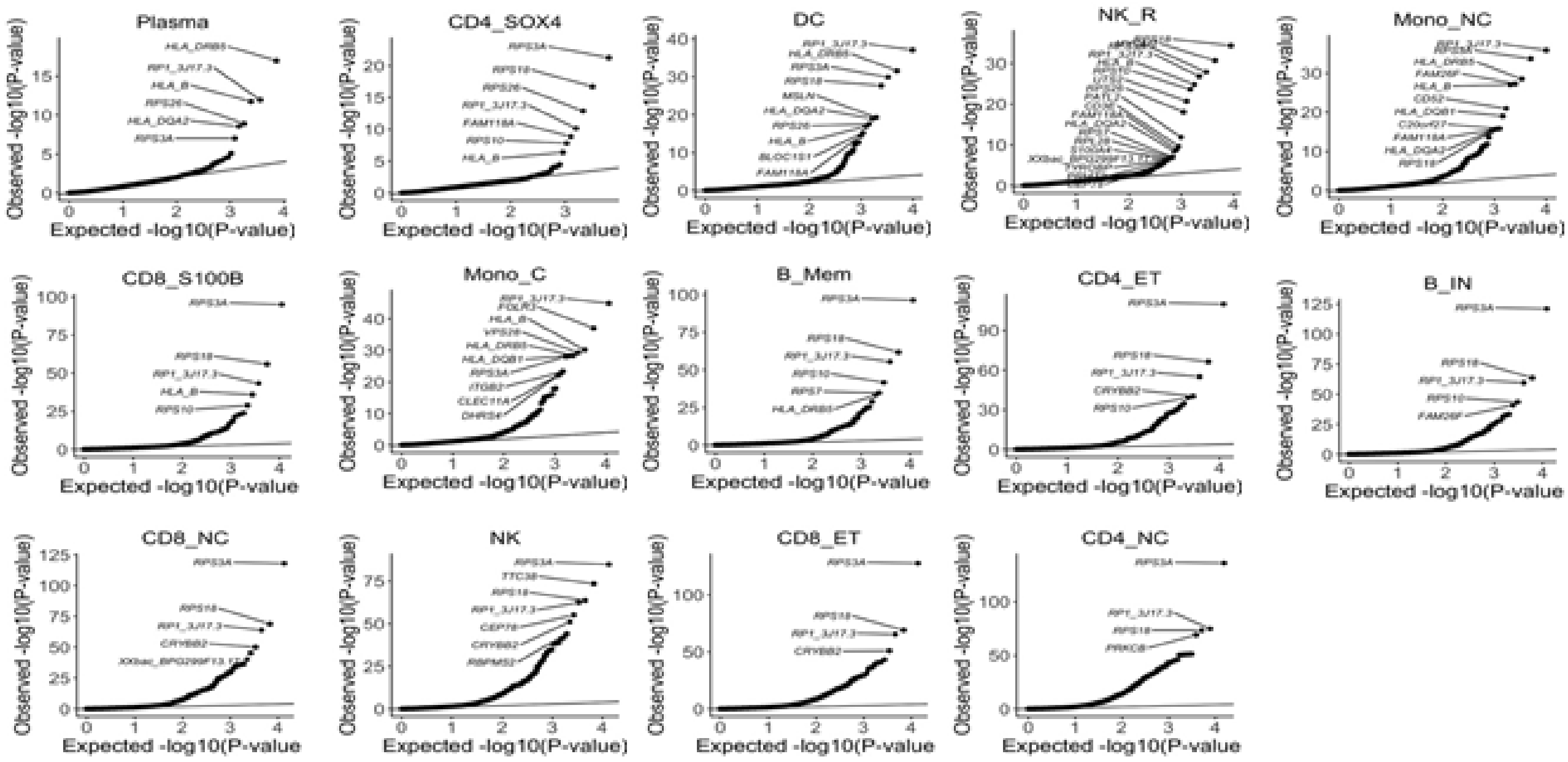

b. Null with permuted sample IDs (set-based tests)

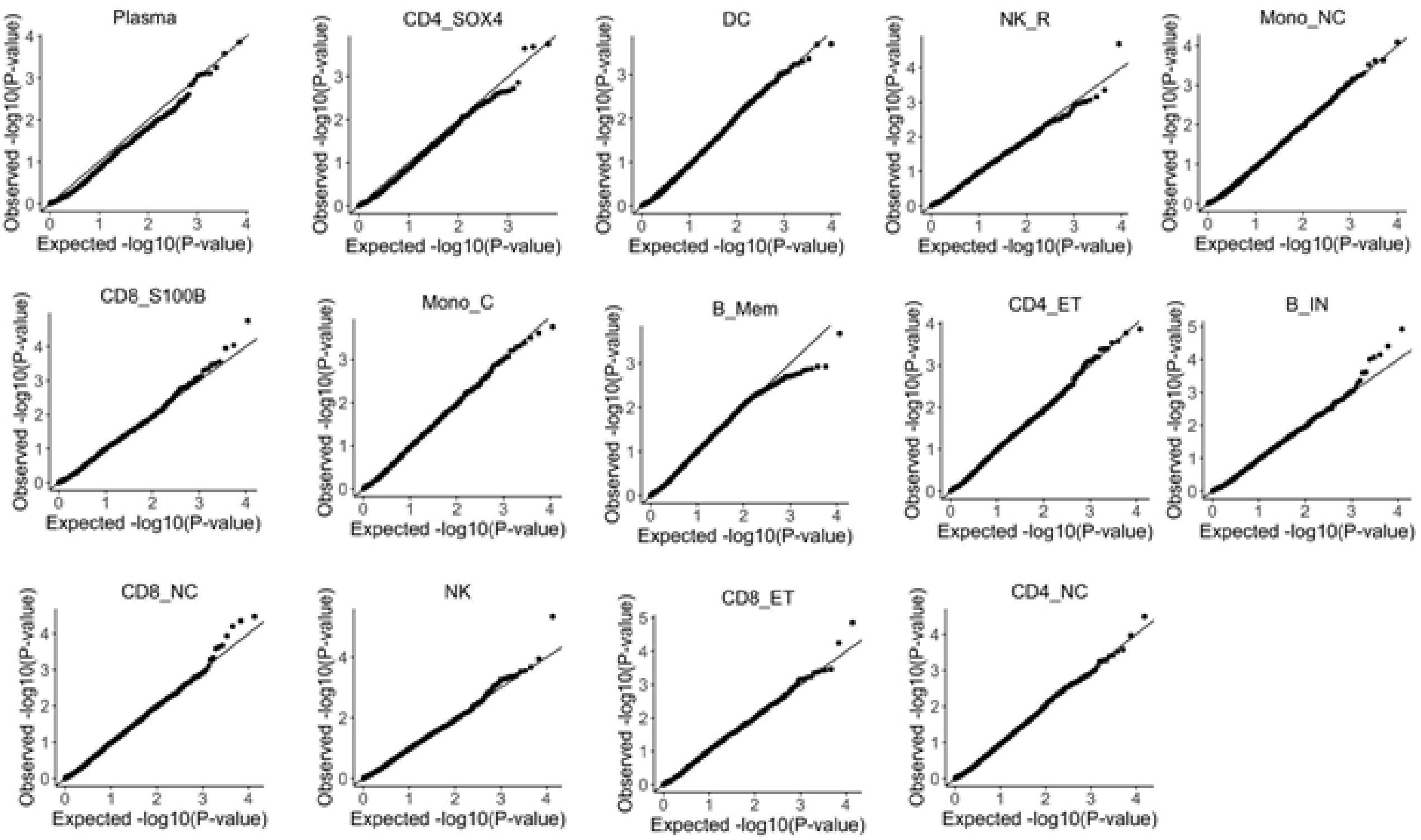

Supplementary Figure 6: QQ plots of set-based test p-values for rare variants (MAF <= 5%) in OneK1K.

a. All genes (expressed in >= 10% of donors) across 14 cell types.

b. As in a, but after permuting genotypes to remove association signal to assess calibration of our test.

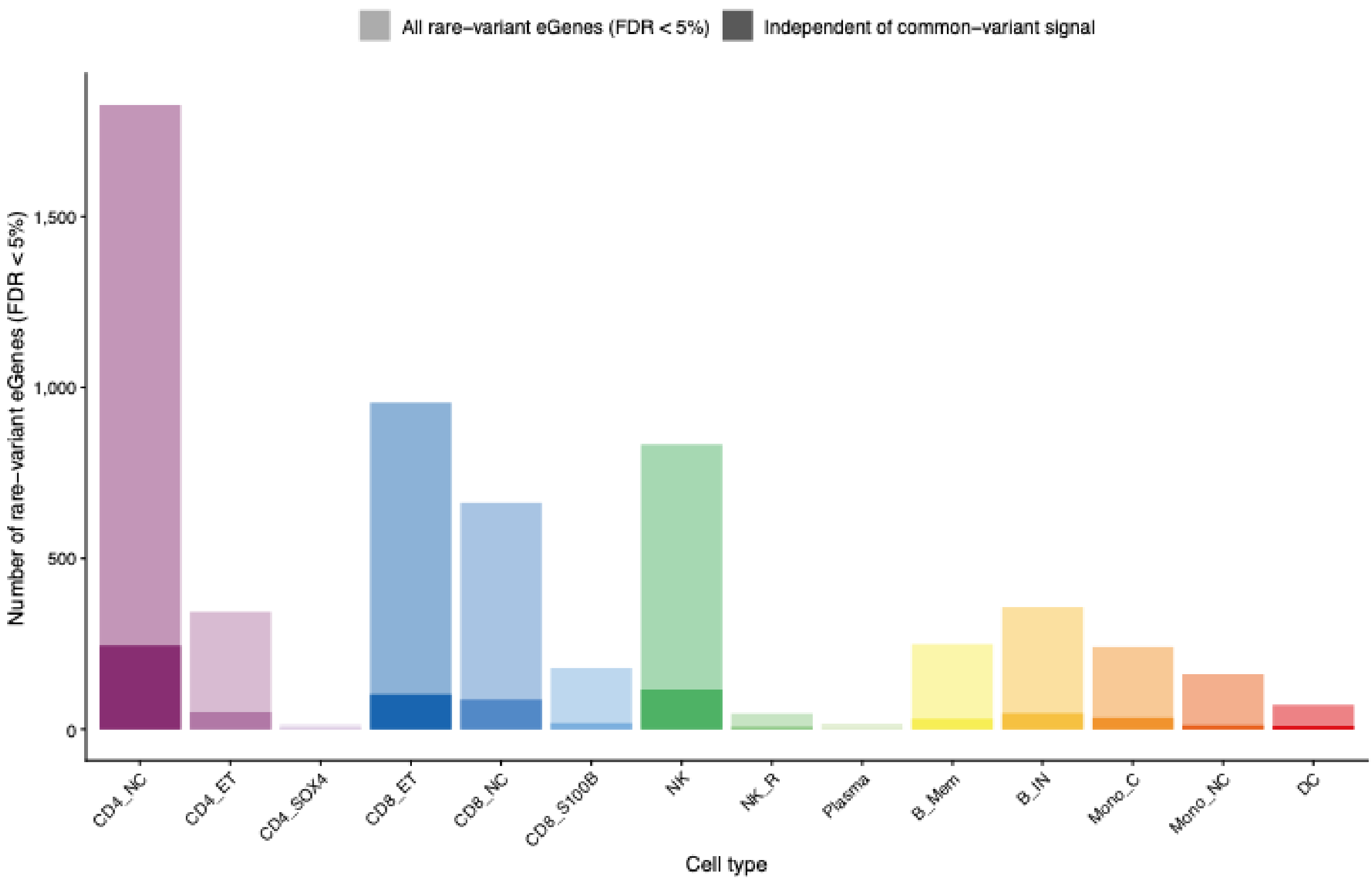

Supplementary Figure 7: Rare variants conditional analysis results.
For each cell type, bars represent the number of rare-variant eGenes (FDR < 5%) identified by our analysis (light shade), and the number that remain significant after conditioning on any significant single-variant signal for the same gene (dark shade).

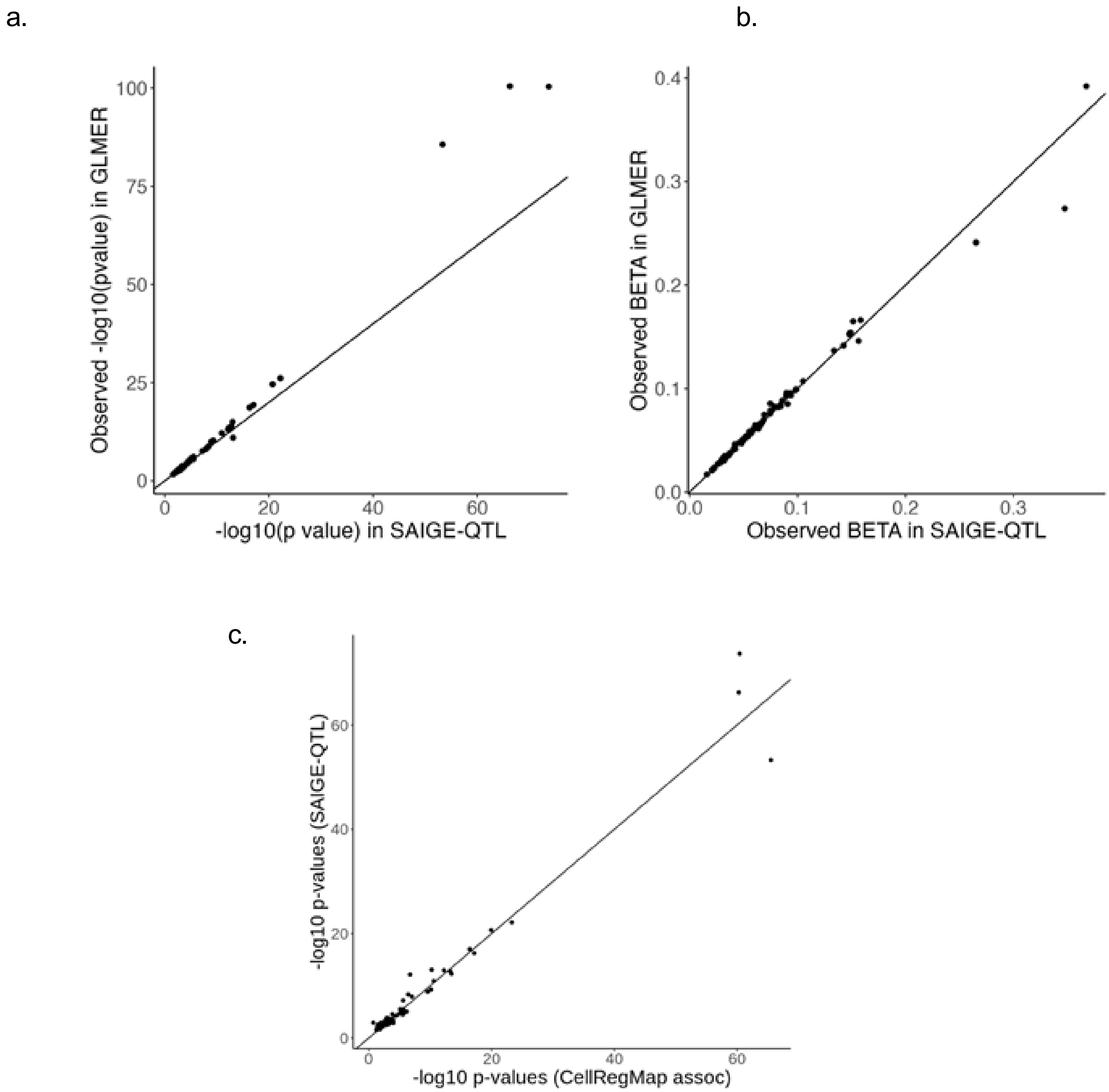

Supplementary Figure 8: Comparing variant P values from SAIGE-QTL to existing single-cell QTL methods.

cis-eQTL mapping were conducted for n=88 randomly selected genes in innate and naïve B cells (“B_{IN}”) and the plots were based on the top hit (most significant variant) for each gene. When different methods had different top hits, the ones from SAIGE-QTL were used. SAIGE-QTL and CellRegMap-association P values are from two-sided score tests; glmer P values are from two-sided Wald tests.

a. Scatter plot of –log10(p-values) from the Poisson mixed models implemented in the glmer function in the lme4 R package and SAIGE-QTL.
b. Scatter plot effect size estimates from the Poisson mixed models implemented in the glmer function in the lme4 R package and SAIGE-QTL.
c. Scatter plot of –log10(p-values) from SAIGE-QTL and CellRegMap-association

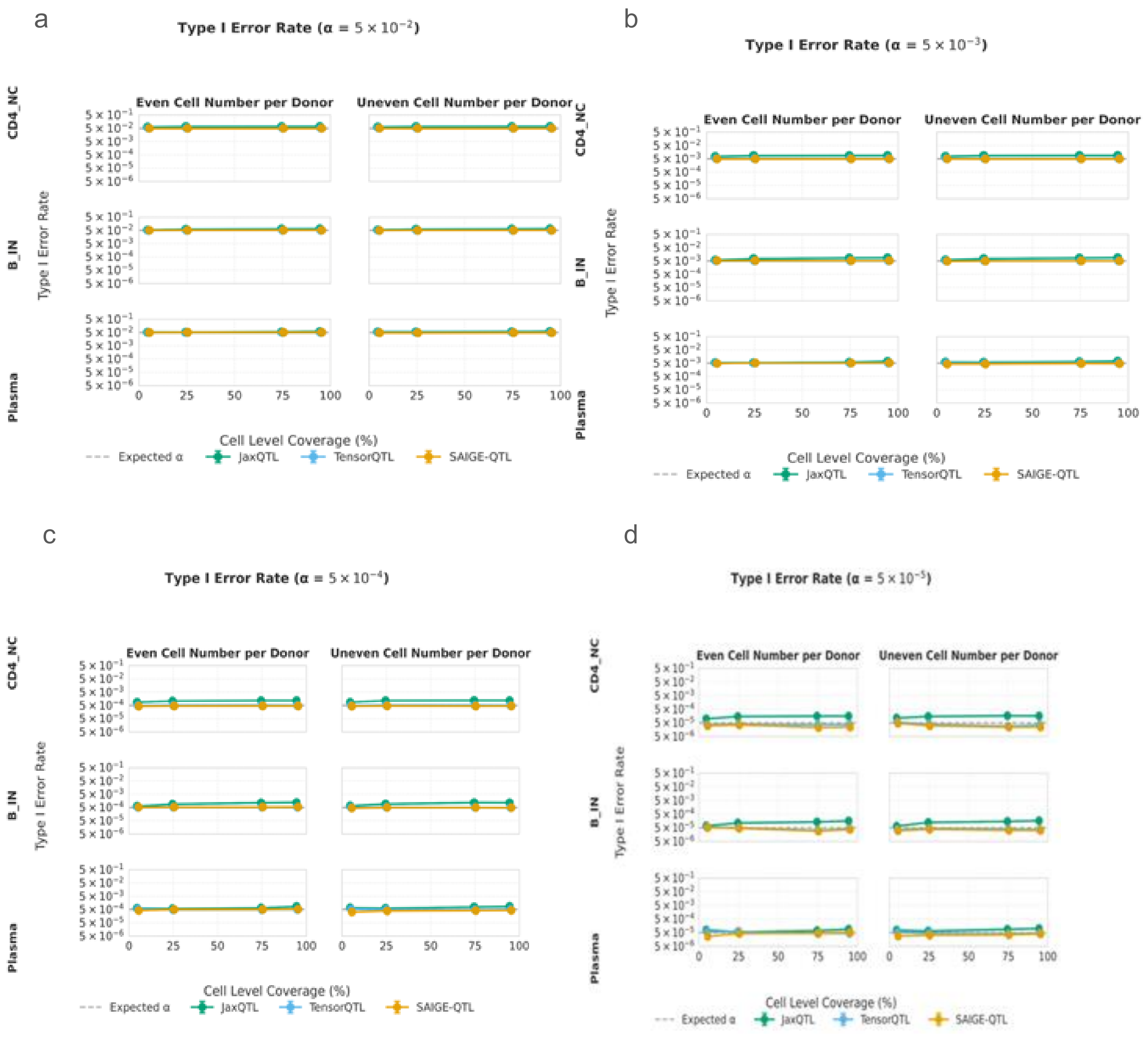

Supplementary Figure 9: Type I error rate evaluation under simulated scenarios comparing SAIGE-QTL, TensorQTL, and JaxQTL.

Panels a-d correspond to Type I Error rate simulated under different nominal significance thresholds (from $5x10^{-2}$ to $5x10^{-5}$). Each scenario was evaluated using n = 500 simulated genes and 2,100 LD-pruned SNPs per replicate (Methods).

For each cell type, under varying cell level non-zero expression coverages (1-sparsity level across cells), SAIGE-QTL maintains a well-controlled Type I error rate even at the most stringent significance thresholds that are typical of genome-wide studies.

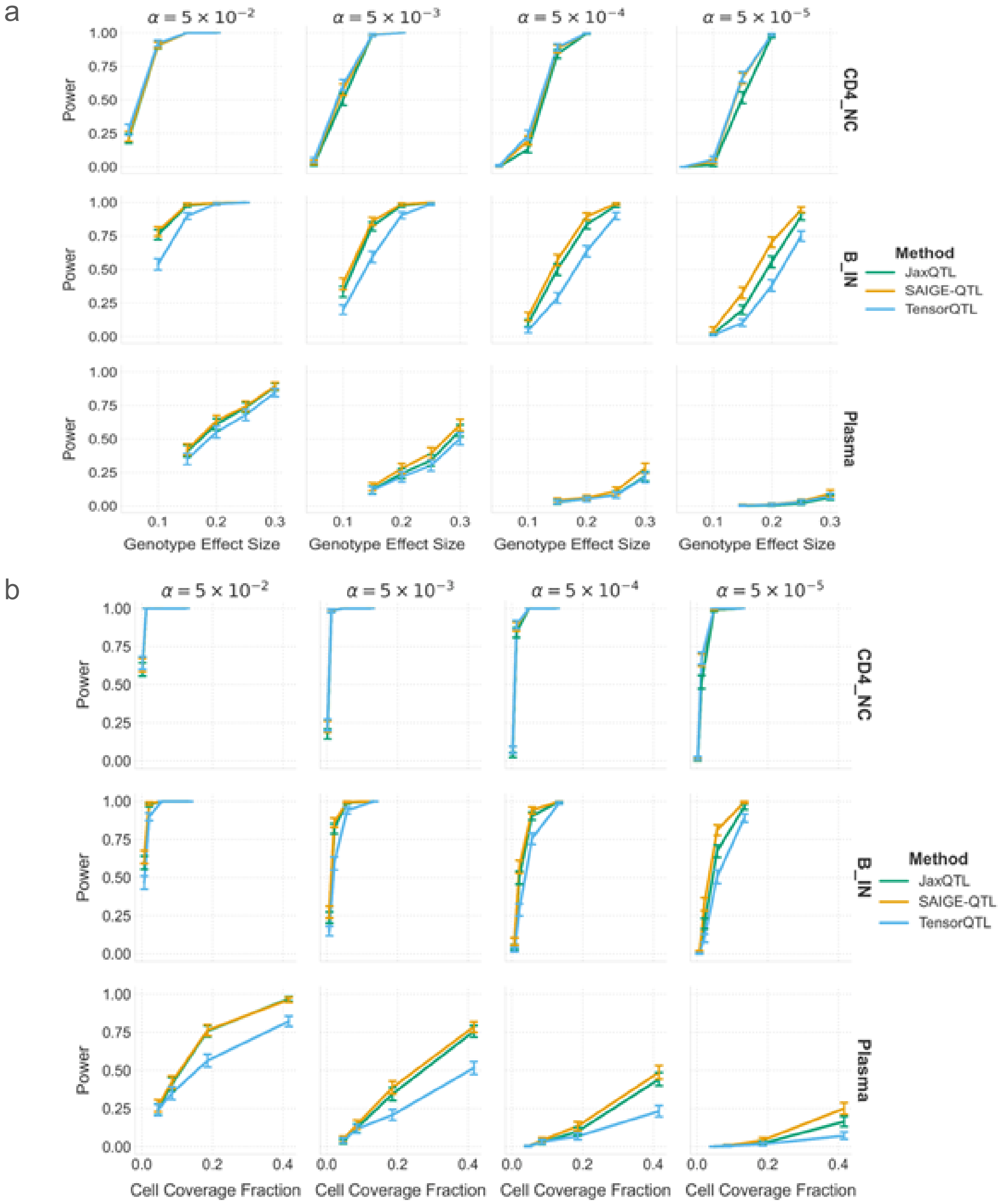

Supplementary Figure 10: Power evaluation across different (a) cell coverage fraction level (1- sparsity levels across cells) and (b) genetic effect sizes for SAIGE-QTL, TensorQTL, and JaxQTL under four significance α levels and three representative cell types.

a: Power given genotype effect sizes. Each plotted dot is the mean power across the 4 corresponding cell-level coverage values simulated at that genotype effect size (x-axis). error bars represent 95% CI across n = 500 simulated genes.

b: Power spanning different cell-level non-zero expression coverages (1-sparsity levels across cells). Each plotted dot is the mean power across the four corresponding genotype effect size values simulated at that cell-level non-zero expression fraction (x-axis). error bars represent 95% CI across n = 500 simulated genes.

Simulations are performed based on the OneK1K dataset for all cell types, which has uneven number of cells per donor. To maintain consistency with our real-data analyses of OneK1K, no cell-level covariates were included in the simulations.

SAIGE-QTL maintained highest power compared to the other two methods by directly modeling read counts from cells, especially in less abundant cell types (B_{IN} and Plasma)

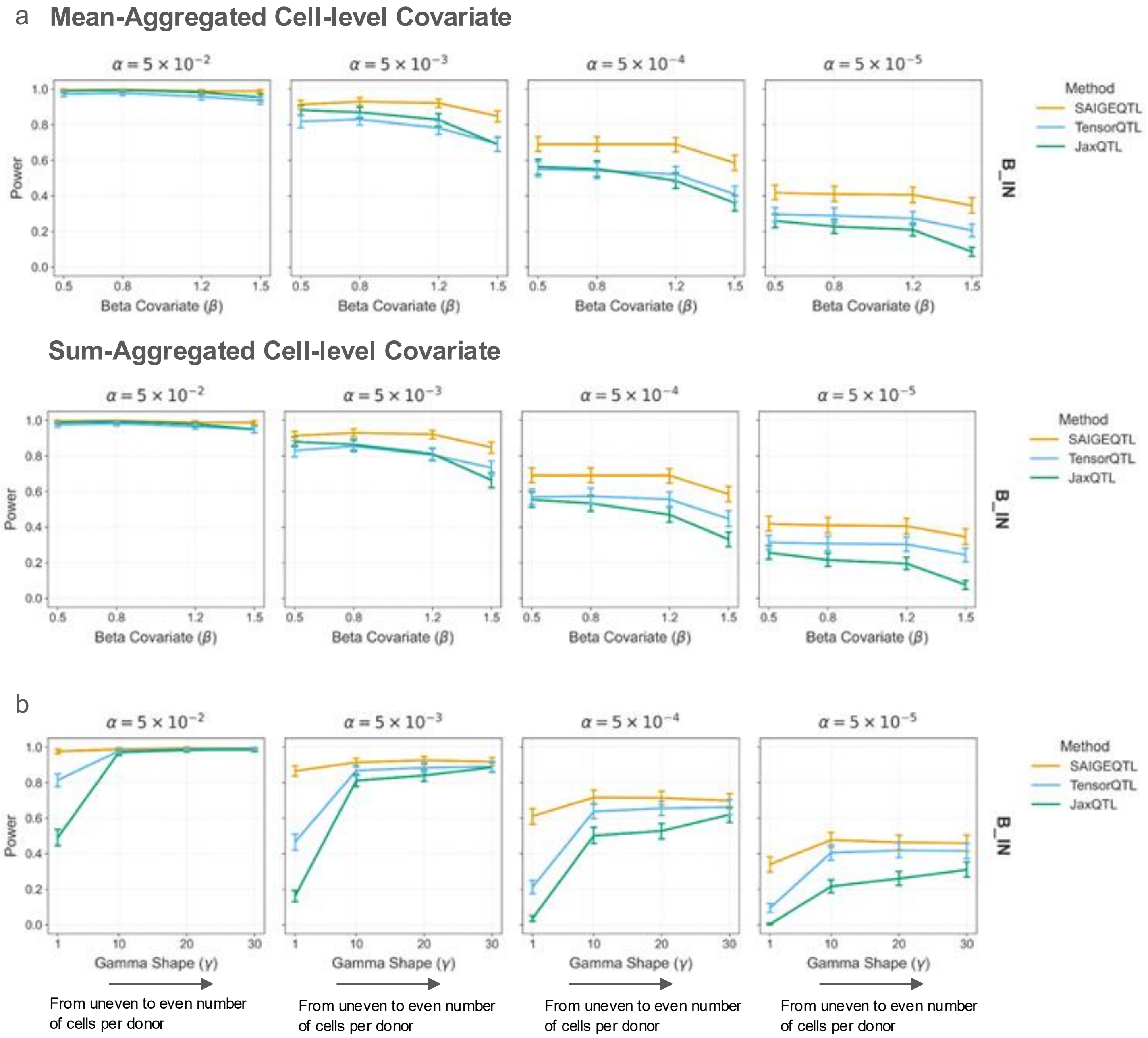

Supplementary Figure 11: Power evaluation under scenarios with (a) cell-level covariates and (b) from uneven to even number of cells per donor for SAIGE-QTL, TensorQTL, and JaxQTL.

a. Power plots with varying effect sizes of a simulated cell-level covariate (the covariate is either mean- or sum-aggregated to the donor-level to accommodate for pseudo-bulk method usages). Error bars represent 95% CI based on n=500 simulated B_{IN} cell-type datasets (Methods).

b. Power plots with the number of cells per donor spanning from an uneven to a more even distribution (left to right, increasing Gamma shape parameter γ leading to a more even distribution). Error bars represent 95% CI based on n=500 simulated B_{IN} cell-type datasets (Methods).

SAIGE-QTL shows a power advantage over pseudo-bulk methods in scenarios of within-donor variation, including the presence of cell-level covariates and unbalanced cell number per donor. The power advantage became more evident as within-donor covariates effects on expression increased, and as unevenness of cell-count distribution increased.

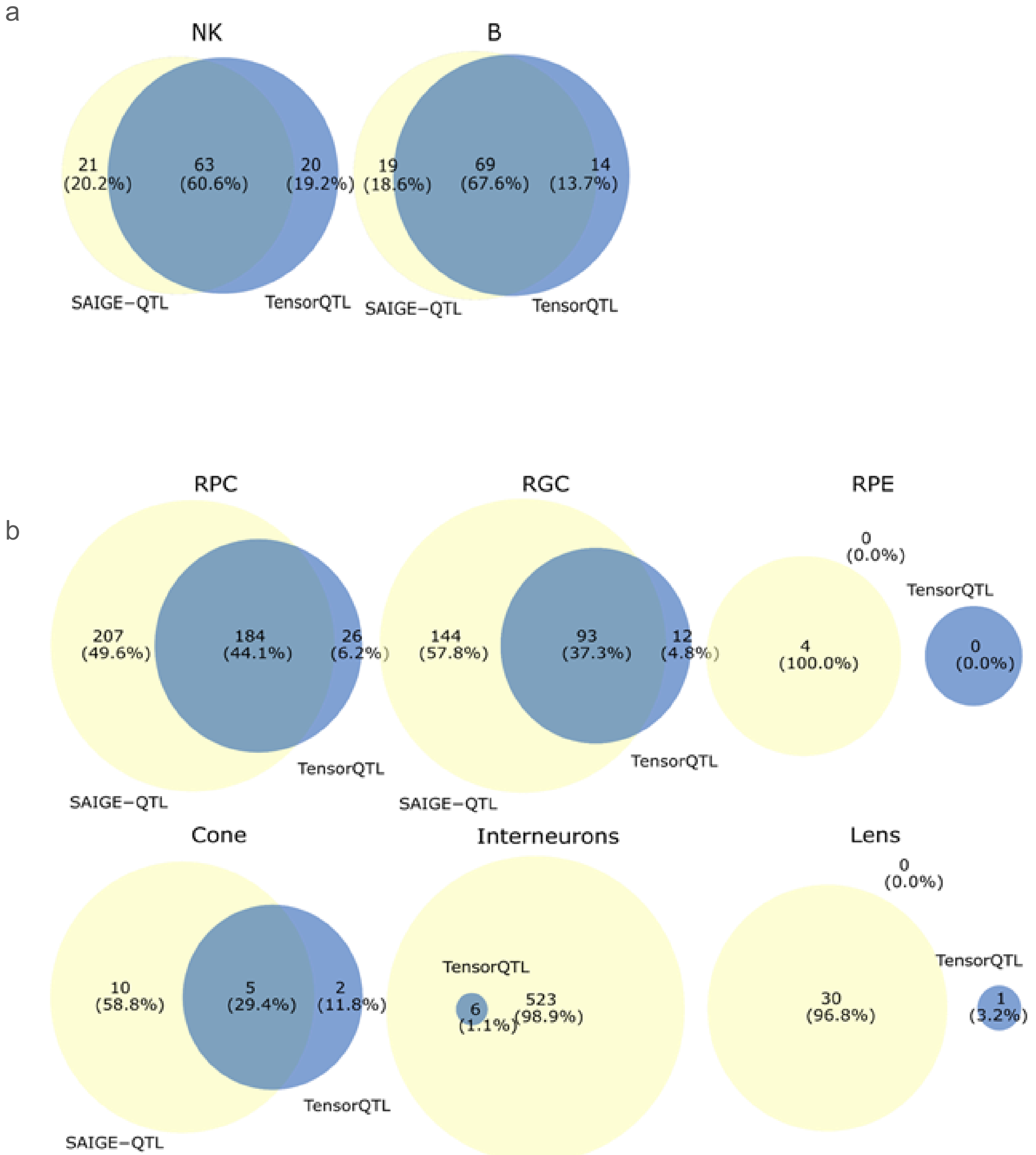

Supplementary Figure 12: Number of eGenes identified in PBMC and retinal cells using SAIGE-QTL and TensorQTL.

a. Venn diagrams representing the overlap of eGenes identified based on eQTLs detected using each of the two methods, for the systemic lupus erythematosus (SLE) disease considered.

b. Venn diagrams representing the overlap of eGenes identified based on eQTLs detected using each of the two methods, for the primary open-angle glaucoma (POAG) disease considered.

For all cell types in PBMC and retinol cells, more unique eGenes were detected to contribute to the disease of interest using results from SAIGE-QTL. And the majority of the eGenes identified through TensorQTL was also detected through SAIGE-QTL.

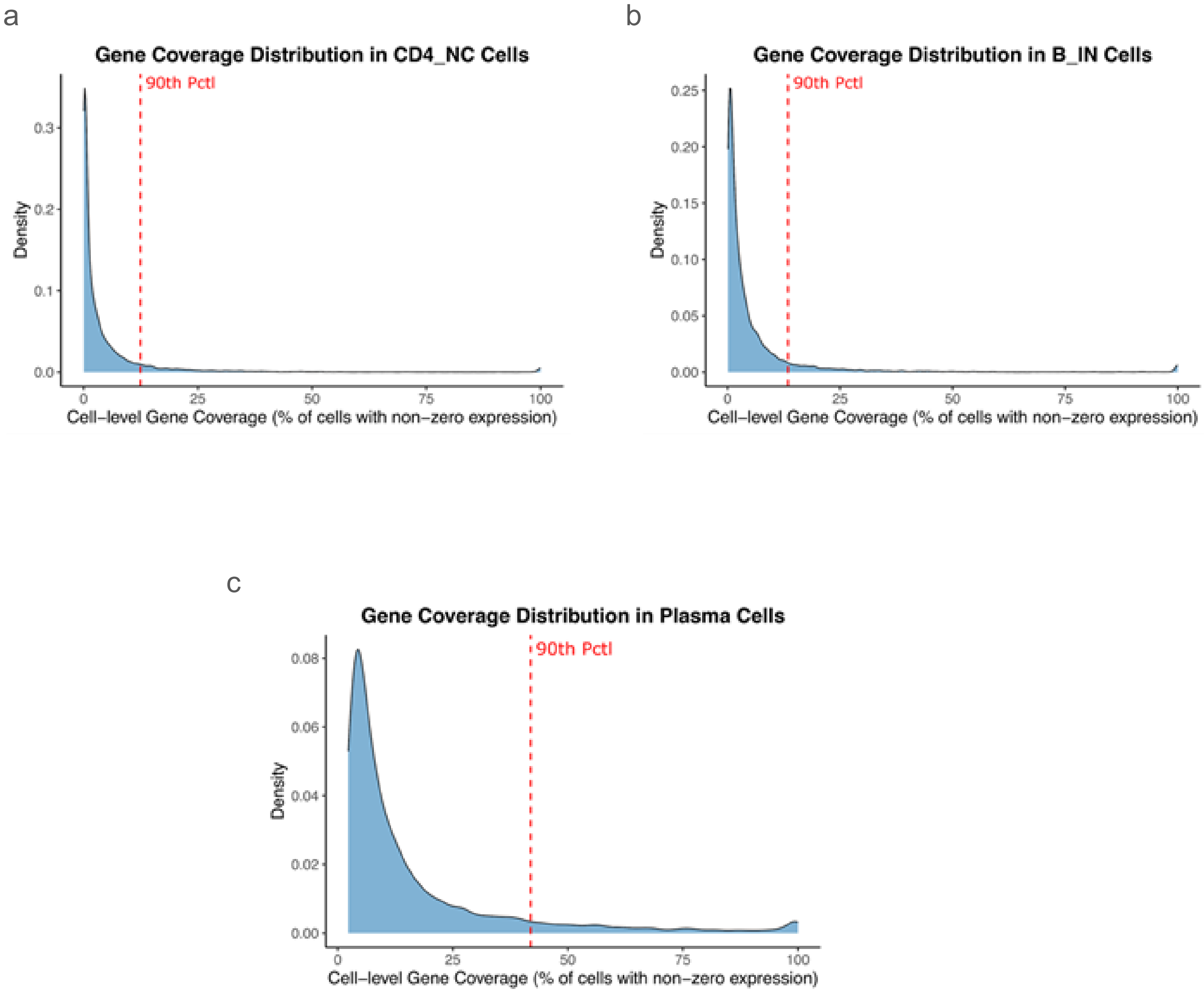

Supplementary Figure 13: Distribution of cell-level coverages across representative cell types.

Density plots show the distribution of genes' cell-level coverage, defined as the percentage of cells with non-zero expression for each gene, across three representative three cell types: a. $CD4_{NC}$, b. B_{IN}, c. Plasma cells. Only genes with donor-level non-zero expression coverage exceeding 10% were included in this plot. Red dashed vertical lines indicate the 90th percentile of the cell-level coverage distribution for each cell type.

Across all cell types, cell-level coverages are heavily right-skewed, with the majority of genes expressed in lower fraction of cells, motivating the range of target coverage levels used in power simulation studies.

Efficient and accurate mixed model association tool for single-cell eQTL analysis
Supplementary Note

1 Notations of the SAIGE-QTL method

Assuming the data contain read counts from RNA sequencing mapped to a gene for N cells across n individuals, c_i cells belong to the ith individual and $N = \sum_{i=n}^{n} c_i$. The sequencing read count for the gene of interest, y_{ij}, of the jth cell from the ith individual is assumed to follow Poisson(μ_{ij}), where $\mu_{ij} = exp\left(\eta_{ij}\right)$ and $\eta_{ij} = X_{cij}\alpha_c + X_{di}\alpha_d + G_i\beta + b_i$. Let G_i represent the allele count (0,1, or 2) of the ith individual for the genetic variant to test, X_{cij} represent p_c cell-level covariates, such as cell states, including the intercept for the jth cell of the ith individual, and X_{di} represent p_d individual (donor)-level covariates, such as age, gender, and ancestry PCs, for the ith individual. The random effects $\boldsymbol{b_d}$ is a length n vector of b_i and is assumed to follow multivariate Normal distribution $b_i \sim N(0, \sum_{k=1}^{K} \tau_k \boldsymbol{\Psi}_k)$, where τ_k are the variance component parameters and $\boldsymbol{\Psi}_k$ are known $n \times n$ variance-covariance matrices, which can include the identity matrix to account for intra-individual variance of read counts across multiple cells and the genetic relationship matrix to account for sample relatedness, if any.

The read counts y_{ij} are assumed to be independent conditional on the random effect $(X_{cij}, X_{di}, G_i, b_i)$ and follows the Poisson distribution with mean and variance $E(y_{ij}|X_{cij}, X_{di}, G_i, b_i) = \mu_{ij}$ and $Var(y_{ij}|X_{cij}, X_{di}, G_i, b_i) = \phi v(\mu_{ij})$, where $v(\mu_{ij}) = \mu_{ij}$ is the variance function and the dispersion parameter $\phi = 1$.

Let $\boldsymbol{Z}$ be the $N \times n$ design matrix. Each row in $\boldsymbol{Z}$ contains a 1 and all other elements are 0s to indicate which individual has the cell. The Poisson mixed model can be written as

$$\boldsymbol{\eta} = \boldsymbol{X_c}\boldsymbol{\alpha_c} + \boldsymbol{Z}\boldsymbol{X_d}\boldsymbol{\alpha_d} + \boldsymbol{Z}\boldsymbol{G}\beta + \boldsymbol{b},$$

where $\boldsymbol{\eta}$ is a length of N vector of η_{ij}, $\boldsymbol{X_c}$ is a $N \times (p_c + 1)$ matrix containing cell-level covariates including the intercept, $\boldsymbol{X_d}$ is a $n \times p_d$ matrix containing individual-level covariates, and the random effects for all cells $\boldsymbol{b} = \boldsymbol{Z}\boldsymbol{b_d}$ is a vector with length of N . $\boldsymbol{b} \sim N(0, \sum_{k=1}^{K} \tau_k Z \Psi_k Z^T)$ accounts for all sources of correlation in the data.

1.1 Poisson generalized linear mixed model and penalized quasi-likelihood

Similar algorithms that were used in previous GWAS methods [1, 2, 3] are used in SAIGE-QTL to fit the Poisson generalized linear mixed model under the null hypothesis $H_0 : \beta = 0$. To estimate $(\boldsymbol{\alpha}, \phi, \boldsymbol{\tau})$, where $\boldsymbol{\alpha} = (\boldsymbol{\alpha_c}, \boldsymbol{\alpha_d})$, the integrated quasi-likelihood function can be written as

$$\begin{aligned} L\left(\boldsymbol{\alpha}, \beta = 0, \phi, \boldsymbol{\tau}\right) = & \int exp\{\sum_{i=1}^{n}\sum_{j=1}^{c_i} ql_{ij}(\boldsymbol{\alpha}, \beta = 0|\boldsymbol{b})\} \\ & \times (2\pi)^{-\frac{N}{2}} \frac{1}{\left|\sum_{k=1}^{K} \tau_k \boldsymbol{V_K}\right|^{1/2}} \exp\left(-\frac{1}{2}\mathbf{b}^\top(\sum_{k=1}^{K} \tau_k \boldsymbol{V_K})^{-1}\mathbf{b}\right) d\mathbf{b} \\ = & (2\pi)^{-\frac{N}{2}} \frac{1}{\left|\sum_{k=1}^{K} \tau_k \boldsymbol{V_K}\right|^{1/2}} \\ & \times \int exp\left(\sum_{i=1}^{n}\sum_{j=1}^{c_i} ql_{ij}(\boldsymbol{\alpha}, \beta = 0|\boldsymbol{b}) - \left(\frac{1}{2}\mathbf{b}^\top(\sum_{k=1}^{K} \tau_k \boldsymbol{V_K})^{-1}\mathbf{b}\right)\right) d\mathbf{b} \end{aligned},$$

where $\mathbf{V_k} = \mathbf{Z\Psi_k Z^T}$

$$ql_{ij}(\boldsymbol{\alpha}, \beta = 0|\boldsymbol{b}) = \int_{y_{ij}}^{\mu_{ij}} \frac{(y_{ij} - \mu)}{\phi v(\mu)} d(\mu) = \int_{y_{ij}}^{\mu_{ij}} \frac{y_{ij} - \mu}{\mu} d(\mu)$$

is the quasi-likelihood for the jth cell from the ith individual given the random effect $\boldsymbol{b}$.

Let $f(\boldsymbol{b}) = \sum_{i=1}^{n} \sum_{j=1}^{c_i} ql_{ij}(\boldsymbol{\alpha}, \beta = 0|\boldsymbol{b}) - \frac{1}{2}\mathbf{b}^\top(\sum_{k=1}^{K} \tau_k \boldsymbol{V_K})^{-1}\mathbf{b}$, we can then approximate the integral using the Laplace approximation,

$$\int \exp f(\mathbf{b}) db \approx (2\pi)^{N/2} \left| -f''(\tilde{\mathbf{b}}) \right|^{-1/2} \exp(f(\tilde{\mathbf{b}})),$$

where $\tilde{\mathbf{b}} = \arg_b \max f(\mathbf{b})$ is the solution to $f'(\mathbf{b}) = 0$. Therefore, the log integrated quasi-likelihood function can be approximated by

$$ql\left(\alpha, \beta = 0, \phi, \boldsymbol{\tau}\right) \approx -\frac{1}{2} \log \left| \sum_{k=1}^{K} \tau_k \boldsymbol{V_K} \right| - \frac{1}{2} \log \left| -f''(\tilde{\mathbf{b}}) \right| + f(\tilde{\mathbf{b}})$$

$$\frac{\partial ql_{ij}(\boldsymbol{\alpha}, \beta = 0|\boldsymbol{b})}{\partial b} = \frac{\partial ql_{ij}}{\partial \mu_{ij}} \frac{\partial \mu_{ij}}{\partial \eta_{ij}} \frac{\partial \eta_{ij}}{\partial b_{ij}} = \frac{y_{ij} - \mu_{ij}}{\mu_{ij}} \mu_{ij} Z_{ij}^T = (y_{ij} - \mu_{ij}) Z_{ij}^T$$

$$\frac{\partial^2 ql_{ij}(\boldsymbol{\alpha}, \beta = 0|\boldsymbol{b})}{\partial b \partial b^T} = -\frac{\partial \mu_{ij}}{\partial \eta_{ij}} \frac{\partial \eta_{ij}}{\partial b_{ij}} Z^T = -\mu_{ij} Z_{ij} Z_{ij}^T$$

$$f''(\tilde{\mathbf{b}}) = -(\sum_{i=1}^{n} \sum_{j=1}^{c_i} \mu_{ij} Z_{ij} Z_{ij}^T) - (\sum_{k=1}^{K} \tau_k \boldsymbol{V_K})^{-1}$$

$$\begin{aligned} ql\left(\alpha, \beta = 0, \phi, \boldsymbol{\tau}\right) \approx & -\frac{1}{2} \log \left| \sum_{k=1}^{K} \tau_k \boldsymbol{V_K} \right| - \frac{1}{2} \log \left| (\sum_{i=1}^{n} \sum_{j=1}^{c_i} \mu_{ij} Z_{ij} Z_{ij}^T) + (\sum_{k=1}^{K} \tau_k \boldsymbol{V_K})^{-1} \right| \\ & + \sum_{i=1}^{n} \sum_{j=1}^{c_i} ql_{ij}(\boldsymbol{\alpha}, \beta = 0|\tilde{\mathbf{b}}) - \frac{1}{2} \tilde{\mathbf{b}}^\top (\sum_{k=1}^{K} \tau_k \boldsymbol{V_K})^{-1} \tilde{\mathbf{b}} , \\ & = -\frac{1}{2} \log \left| \sum_{k=1}^{K} Z(\tau_k \Psi_k \boldsymbol{W} + I) Z^T \right| + \sum_{i=1}^{n} \sum_{j=1}^{c_i} ql_{ij}(\boldsymbol{\alpha}, \beta = 0|\tilde{\mathbf{b}}) - \frac{1}{2} \tilde{\mathbf{b}}^\top (\sum_{k=1}^{K} \tau_k \boldsymbol{V_K})^{-1} \tilde{\mathbf{b}} \end{aligned} \tag{1}$$

where $\mathbf{W} = diag(\mu)$

1.2 Estimation of the fixed effects and random effects given the variance components

We can obtain the score functions by taking derivatives of $ql\left(\alpha, \beta = 0, \phi, \boldsymbol{\tau}\right)$ with respect to β, α, and b.

$$\begin{aligned} \frac{\partial ql\left(\boldsymbol{\alpha}, \beta = 0, \phi, \boldsymbol{\tau}\right)}{\partial \boldsymbol{\alpha}} =& \boldsymbol{X}^T(\boldsymbol{y} - \boldsymbol{\mu}) \\ \frac{\partial ql\left(\boldsymbol{\alpha}, \beta = 0, \phi, \boldsymbol{\tau}\right)}{\partial \beta} =& \boldsymbol{G}^T(\boldsymbol{y} - \boldsymbol{\mu}) \\ \frac{\partial ql\left(\boldsymbol{\alpha}, \beta = 0, \phi, \boldsymbol{\tau}\right)}{\partial \boldsymbol{b}} =& (\boldsymbol{y} - \boldsymbol{\mu}) - (\sum_{k=1}^{K} \tau_k \boldsymbol{V_K})^{-1} \boldsymbol{b}, \end{aligned}$$

where $\boldsymbol{X} = \begin{bmatrix} \boldsymbol{X_c} & \boldsymbol{ZX_d} \end{bmatrix}$. For the score test of $H_0 : \beta = 0$ vs $H_1 : \beta \neq 0$, we only estimate the $(\hat{\alpha}, \hat{\beta}, \hat{\mathbf{b}})$ that maximize $ql\left(\boldsymbol{\alpha}, \beta = 0, \phi, \boldsymbol{\tau}\right)$ under H_0, setting $\beta = 0$. We denote the working outcome vector $\mathbf{Y} = \boldsymbol{\eta} + \boldsymbol{W}^{-1}(\boldsymbol{y} - \boldsymbol{\mu})$, where $\boldsymbol{W} = diag(\mu_i)$, $\boldsymbol{\eta} = (\eta_1, \ldots, \eta_N)$, $\boldsymbol{\delta} = (\delta_1, \ldots, \delta_N)$, and $\boldsymbol{\mu} = (\mu_1, \ldots, \mu_N)$. Then, $\boldsymbol{y} - \boldsymbol{\mu} = \boldsymbol{W}(\boldsymbol{Y} - \boldsymbol{\eta}) = \boldsymbol{W}(\boldsymbol{Y} - \boldsymbol{X\beta} - \boldsymbol{Zb})$, and the score equations can be written as,

$$\begin{bmatrix} \mathbf{X}^\top \mathbf{W} \mathbf{X} & \mathbf{X}^\top \mathbf{W} \\ \mathbf{W} \mathbf{X} & \mathbf{W} + (\sum_{\mathbf{k=1}}^{\mathbf{K}} \tau_{\mathbf{k}} \boldsymbol{V_K})^{\mathbf{-1}} \end{bmatrix} \begin{bmatrix} \alpha \\ b \end{bmatrix} = \begin{bmatrix} \mathbf{X}^\top \mathbf{W} \mathbf{Y} \\ \mathbf{W} \mathbf{Y} \end{bmatrix}$$

Let $\mathbf{\Sigma} = \mathbf{W^{-1}} + \sum_{\mathbf{k=1}}^{\mathbf{K}} \tau_{\mathbf{k}} \boldsymbol{V_K}$, then

$$\hat{\boldsymbol{\alpha}} = \left(\mathbf{X}^\top \mathbf{\Sigma}^{-\mathbf{1}} \mathbf{X}\right)^{-\mathbf{1}} \mathbf{X}^\top \mathbf{\Sigma}^{-\mathbf{1}} \mathbf{Y}$$
$$\hat{\mathbf{b}} = \left(\sum_{\mathbf{k=1}}^{\mathbf{K}} \tau_{\mathbf{k}} \boldsymbol{V_K}\right) \mathbf{\Sigma}^{-\mathbf{1}} (\mathbf{Y} - \mathbf{X}\hat{\boldsymbol{\alpha}}).$$

1.3 Estimation of the variance component

Given $\hat{\boldsymbol{\alpha}} = \hat{\boldsymbol{\alpha}}(\boldsymbol{\tau}), \hat{\boldsymbol{b}} = \hat{\boldsymbol{b}}(\boldsymbol{\tau})$ estimated, from (1) the log-likelihood of the variance component can be derived as,

$$ql(\hat{\boldsymbol{\alpha}}(\boldsymbol{\tau}, \phi), \beta = 0, \phi, \boldsymbol{\tau}) = c - \frac{1}{2} log|\mathbf{\Sigma}| - \frac{\mathbf{1}}{\mathbf{2}} \mathbf{Y}^\top \mathbf{P} \mathbf{Y},$$

where $\mathbf{P} = \mathbf{\Sigma}^{-\mathbf{1}} - \mathbf{\Sigma}^{-\mathbf{1}} \mathbf{X} \left(\mathbf{X}^\top \mathbf{\Sigma}^{-\mathbf{1}} \mathbf{X}\right)^{-\mathbf{1}} \mathbf{X}^\top \mathbf{\Sigma}^{-\mathbf{1}}$ and $\boldsymbol{\Sigma} = \boldsymbol{W}^{-\mathbf{1}} + \sum_{\boldsymbol{k=1}}^{\boldsymbol{K}} \boldsymbol{\tau_k} \boldsymbol{V_k}$. We maximize the corresponding restricted maximum-likelihood (REML),

$$ql_R(\hat{\boldsymbol{\alpha}}(\boldsymbol{\tau}, \phi), \beta = 0, \phi, \boldsymbol{\tau}) = c_R - \frac{1}{2} log|\mathbf{\Sigma}| - \frac{1}{2} log|\mathbf{X}^\top \mathbf{\Sigma}^{-\mathbf{1}} \mathbf{X}| - \frac{\mathbf{1}}{\mathbf{2}} \mathbf{Y}^\top \mathbf{P} \mathbf{Y}.$$

The score function with respect to τ are given by,

$$\mathbf{U}_\tau = \frac{\partial ql_R(\hat{\boldsymbol{\alpha}}(\boldsymbol{\tau}, \phi), \beta = 0, \phi, \boldsymbol{\tau})}{\partial \tau_k} = \frac{1}{2} \left[\mathbf{Y}^\top \mathbf{P} \mathbf{Z} \mathbf{\Psi_k} \mathbf{Z^T} \mathbf{P} \mathbf{Y} - tr(\mathbf{P} \mathbf{Z} \mathbf{\Psi_k} \mathbf{Z^T})\right].$$

The corresponding observed information function, and the expected information function are given by

$$\mathbf{J}_\tau = -\frac{\partial^2 ql_R(\hat{\boldsymbol{\alpha}}(\boldsymbol{\tau}, \phi), \beta = 0, \phi, \boldsymbol{\tau})}{\partial \tau_k^2} = -\frac{1}{2} tr(\mathbf{P} \mathbf{V_k} \mathbf{P} \mathbf{V_k}) + \mathbf{Y}^\top \mathbf{P} \mathbf{V_k} \mathbf{P} \mathbf{V_k} \mathbf{P} \mathbf{Y},$$
$$E\left(\mathbf{J}_\tau\right) = E\left[-\frac{\partial^2 ql_R(\hat{\boldsymbol{\alpha}}(\boldsymbol{\tau}, \phi), \beta = 0, \phi, \boldsymbol{\tau})}{\partial \tau_k^2}\right] = \frac{1}{2} tr(\mathbf{P} \mathbf{V_k} \mathbf{P} \mathbf{V_k}),$$

respectively. Evaluating both observed and expected information functions involves computationally expensive trace computations. To avoid the trace computations, the average information is used in the AI-REML[1, 2, 4] algorithm. The average information is expressed as the average of J_τ and $E(J_\tau)$,

$$\mathbf{AI}_{\tau_k \tau_q} = \frac{1}{2} \mathbf{Y}^\top \mathbf{P} \mathbf{V_k} \mathbf{P} \mathbf{V_q} \mathbf{P} \mathbf{Y},$$

where $\mathbf{AI}$ is an $K \times K$ matrix with the (k, q)-th element $\mathbf{AI}_{\tau_k \tau_q}$.

1.3.1 Algorithm to fit the null mixed model

The null model fitting algorithm can be summarized as,

1. Fit a Poisson linear model with $\boldsymbol{\tau} = 0$ to get initial estimates $\hat{\boldsymbol{\alpha}}^{(0)}$ and working outcome vector $Y^{(0)}$.
2. At the i-th step, update $\hat{\boldsymbol{\tau}}$ using $\hat{\boldsymbol{\tau}}^{(i)} = \hat{\boldsymbol{\tau}}^{(i-1)} + \left\{\mathbf{AI}_{\boldsymbol{\tau}}\Big|_{\boldsymbol{\tau} = \hat{\boldsymbol{\tau}}^{(i-1)}}\right\}^{-1} \left\{\mathbf{U}_{\boldsymbol{\tau}}\Big|_{\boldsymbol{\tau} = \hat{\boldsymbol{\tau}}^{(i-1)}}\right\}$.
3. Update $\hat{\boldsymbol{\alpha}}$, $\hat{\boldsymbol{b}}$ using $\mathbf{Y}$ and $\hat{\boldsymbol{\tau}}^{(i)}$.
4. Update $\mathbf{Y}$ using $\hat{\boldsymbol{b}}^{(i)}, \hat{\boldsymbol{\alpha}}^{(i)}, \hat{\boldsymbol{\tau}}^{(i)}$.
5. Repeat steps 2–4, until $\max\left\{\frac{|\hat{\alpha}^{(i)} - \hat{\alpha}^{(i-1)}|}{|\hat{\alpha}^{(i)}| + |\hat{\alpha}^{(i-1)}|}, \frac{|\hat{\boldsymbol{\tau}}^{(i)} - \hat{\boldsymbol{\tau}}^{(i-1)}|}{|\hat{\boldsymbol{\tau}}^{(i)}| + |\hat{\boldsymbol{\tau}}^{(i-1)}|}\right\} \leq tolerance.$

1.4 Score test

The score test statistic under the null hypothesis is given by,

$$T = \frac{\partial ql}{\partial \beta}\Big|_{\left(\hat{b}, \beta = 0, \hat{\alpha}, \hat{\tau}\right)} = \mathbf{G}^\top \mathbf{Z}^\top (\mathbf{y} - \hat{\mu}) = \tilde{\mathbf{G}}^\top (\mathbf{y} - \hat{\mu}), \tag{2}$$

where $\tilde{\mathbf{G}} = \mathbf{ZG} - \mathbf{X}\left(\mathbf{X}^\top\hat{\mathbf{W}}\mathbf{X}\right)^{-1}\mathbf{X}^\top\hat{\mathbf{W}}\mathbf{ZG}$ is the covariate adjusted genotype vector, and $\boldsymbol{X} = \begin{bmatrix}\boldsymbol{X_c} & \boldsymbol{ZX_d}\end{bmatrix}$ is the covariate matrix with intercept included. The information matrix corresponding to the score equations in (2) is given by,

$$\mathbf{I}(\alpha,\beta,b) = \begin{bmatrix} \mathbf{X}^\top\mathbf{WX} & \mathbf{X}^\top\mathbf{WG} & \mathbf{X}^\top\mathbf{W} \\ \mathbf{G}^\top\mathbf{WX} & \mathbf{G}^\top\mathbf{WG} & \mathbf{G}^\top\mathbf{W} \\ \mathbf{WX} & \mathbf{Z}^\top\mathbf{WG} & \mathbf{W} + (\sum_{\mathbf{k=1}}^{\mathbf{K}}\tau_{\mathbf{k}}\mathbf{V_k})^{-1} \end{bmatrix},$$

A_{ij} and $A_{\bullet i}$ denote the (i,j)-th element and i-th column of a matrix A, respectively. Then, the variance of the score statistic under H_0 is given by,

$$Var_{H_0}(T) = \left(\mathbf{I}(\hat{\alpha},\hat{\beta},\hat{\mathbf{b}})^{-1}\right)_{\mathbf{22}} = \mathbf{G}^\top\hat{\mathbf{P}}\mathbf{G} = \tilde{\mathbf{G}}^\top\hat{\mathbf{P}}\tilde{\mathbf{G}},$$

where $\hat{\mathbf{P}} = \hat{\mathbf{\Sigma}}^{-1} - \hat{\mathbf{\Sigma}}^{-1}\mathbf{X}\left(\mathbf{X}^\top\mathbf{\Sigma}^{-1}\mathbf{X}\right)^{-1}\mathbf{X}^\top\hat{\mathbf{\Sigma}}^{-1}$, $\hat{\mathbf{\Sigma}} = \hat{\mathbf{W}}^{-1} + \sum_{\mathbf{k=1}}^{\mathbf{K}}\hat{\tau}_{\mathbf{k}}\mathbf{V_k}$, and $\mathbf{V_k} = \mathbf{Z\Psi_kZ^T}$.

2 Variance ratio approximation

Computation of the variance of the score statistic $Var_{H_0}(T) = \tilde{\mathbf{G}}^\top\hat{\mathbf{P}}\tilde{\mathbf{G}}$ requires calculating $\hat{\mathbf{P}}\tilde{\mathbf{G}}$ repeatedly for all markers, which is computationally expensive. To avoid calculating $\hat{\mathbf{P}}\tilde{\mathbf{G}}$ for all the markers, previous linear mixed model [5, 6, 7] and logistic mixed model methods [2] first estimate the variance ratio $\hat{r} = \tilde{\mathbf{G}}^\top\hat{\mathbf{P}}\tilde{\mathbf{G}}/\tilde{\mathbf{G}}^\top\hat{\mathbf{W}}\tilde{\mathbf{G}}$ using a small set of markers, and then approximate the variance of the score statistic for all markers by $\hat{r}\tilde{\mathbf{G}}^\top\hat{\mathbf{W}}\tilde{\mathbf{G}}$ in step 2. It has been shown in the studies that $\hat{r}$ is approximately constant for all genetic variants. This saves substantial computation time since $\hat{\mathbf{W}}$ is a diagonal matrix. Following the theoretical justification by Dey et al.[3] to show the approximation also works in the frailty models, we can demonstrate that it also works in the Poisson mixed model for eQTL mapping with scRNA-seq data.

Let $E(G_i) = \mu_g$ and the covariance matrix of $\mathbf{G}$ is given by $\sigma_g^2\mathbf{\Psi}$, where $\mathbf{\Psi}$ is the correlation matrix of $\mathbf{G}$ and represents the $n \times n$ kinship matrix. The exact characterization of $\mathbf{\Psi}$ is not needed for the proof. Then the elements of $\mathbf{G}$ follows the $Bin(2, p_g)$ distribution, then $\mu_g = 2p_g$, and $\sigma_g^2 = 2p_g(1-p_g)$. Let $\mathbf{G_c}$ be the $n\times 1$ unadjusted (but mean-centered) genotype vector. Then $E(\mathbf{G_c}) = 0$ and $Cov(\mathbf{G_c}) = \sigma_g^2\mathbf{\Psi}$. Let $\mathbf{Q}_{\tilde{\mathbf{X}}} = \mathbf{X}(\mathbf{X}^\top\hat{\mathbf{W}}\mathbf{X})^{-1}\mathbf{X}^\top\hat{\mathbf{W}}$ be the weighted projection matrix. Then, $E(\tilde{\mathbf{G}}) = (\mathbf{I} - \mathbf{Q}_{\tilde{\mathbf{X}}})\mathbf{Z}\mu_g\mathbf{1} = 0$, and $Cov(\tilde{\mathbf{G}}) = \sigma_g^2(\mathbf{I} - \mathbf{Q}_{\tilde{\mathbf{X}}})\mathbf{Z\Psi Z^T}(\mathbf{I} - \mathbf{Q}_{\tilde{\mathbf{X}}})^\top$, where $\mathbf{1}$ is the $n \times 1$ vector of all element equal to unity. We scale both the numerator and denominator of the variance ratio by N^{-1} so that they don't blow to infinity when looked at individually. Then, for the numerator,

$$E(N^{-1}\tilde{\mathbf{G}}^\top\hat{\mathbf{P}}\tilde{\mathbf{G}}) = \frac{\sigma_g^2}{N}tr\left[\hat{\mathbf{P}}(\mathbf{I} - \mathbf{Q}_{\tilde{\mathbf{X}}})\mathbf{Z\Psi Z^T}(\mathbf{I} - \mathbf{Q}_{\tilde{\mathbf{X}}})^\top\right] = \frac{\sigma_g^2}{N}tr(\hat{\mathbf{P}}\mathbf{Z\Psi Z^T}),$$

since $(\mathbf{I} - \mathbf{Q}_{\tilde{\mathbf{X}}})^\top\hat{\mathbf{P}}(\mathbf{I} - \mathbf{Q}_{\tilde{\mathbf{X}}}) = \hat{\mathbf{P}}$. Similarly, for the denominator,

$$E(N^{-1}\mathbf{G_c}^\top\mathbf{Z}^\top\hat{\mathbf{W}}\mathbf{ZG_c}) = \frac{\sigma_g^2}{N}tr\left(\mathbf{Z}^\top\hat{\mathbf{W}}\mathbf{Z\Psi}\right),$$

As the eigenvalues of $\hat{\mathbf{P}}, \hat{\mathbf{W}}, \mathbf{\Psi}$ are bounded, and the distribution of $\mathbf{G}$ has bounded support, the variances of the numerator and the denominator terms are both $O(N^{-1})$, and the variance ratio converges to,

$$\hat{r} = \frac{\tilde{\mathbf{G}}^\top\hat{\mathbf{P}}\tilde{\mathbf{G}}}{\mathbf{G_c}^\top\mathbf{Z}^\top\hat{\mathbf{W}}\mathbf{ZG_c}} \xrightarrow{p} \frac{lim_{N\to\infty}\left\{N^{-1}tr(\hat{\mathbf{P}}\mathbf{Z\Psi Z^T})\right\}}{lim_{N\to\infty}\left\{N^{-1}tr\left(\mathbf{Z}^\top\hat{\mathbf{W}}\mathbf{Z\Psi}\right)\right\}}.$$

The ratio on the right-hand side is constant across all markers as the individual limits in the numerator and denominator exist and are bounded away from zero.

In addition, as we have previously shown in SAIGE-GENE[8], the variation of the estimated variance ratio can be smaller when $\hat{\mathbf{\Sigma}}^{-1}$ is used in the denominator than when only $\hat{\mathbf{W}}$ is used. Therefore, in SAIGE-QTL, we incorporate $\hat{\mathbf{\Sigma}}^{-1}$ and the covariate adjusted genotype vector into the denominator to approximate the $Var_{H_0}(T)$ with a smaller variation. More specifically, the variance ratio $\hat{r_s} = \tilde{\mathbf{G}}^\top\hat{\mathbf{P}}\tilde{\mathbf{G}}/\tilde{\mathbf{G}}_{\mathbf{d}}^\top\hat{\mathbf{\Sigma}}^{-1}\tilde{\mathbf{G}}_{\mathbf{d}}$ is estimated. Let $\boldsymbol{\tilde{X}_d} = \begin{bmatrix}\mathbf{1} & \boldsymbol{X_d}\end{bmatrix}$ represent the donor-level covariate matrix with intercept included with the size $n \times (p_d + 1)$, then $\tilde{\mathbf{G}}_{\mathbf{d}}$ is the donor-level

covariate adjusted genotype vector with size $N \times 1$ and

$$\tilde{\mathbf{G}}_d = \mathbf{ZG} - \mathbf{Z}\tilde{\mathbf{X}}_\mathbf{d}\left(\tilde{\mathbf{X}}_\mathbf{d}^\top \mathbf{Z}^\top \hat{\mathbf{W}}\mathbf{Z}\tilde{\mathbf{X}}_\mathbf{d}\right)^{-1}\tilde{\mathbf{X}}_\mathbf{d}^\top \mathbf{Z}^\top \hat{\mathbf{W}}\mathbf{ZG}$$
$$= \mathbf{Z}(\mathbf{G} - \tilde{\mathbf{X}}_\mathbf{d}\left(\tilde{\mathbf{X}}_\mathbf{d}^\top \mathbf{Z}^\top \hat{\mathbf{W}}\mathbf{Z}\tilde{\mathbf{X}}_\mathbf{d}\right)^{-1}\tilde{\mathbf{X}}_\mathbf{d}^\top \mathbf{Z}^\top \hat{\mathbf{W}}\mathbf{ZG})$$

Similarly, Let $\mathbf{Q}_{\tilde{\mathbf{X}}_\mathbf{d}} = \tilde{\mathbf{X}}_\mathbf{d}\left(\tilde{\mathbf{X}}_\mathbf{d}^\top \mathbf{Z}^\top \hat{\mathbf{W}}\mathbf{Z}\tilde{\mathbf{X}}_\mathbf{d}\right)^{-1}\tilde{\mathbf{X}}_\mathbf{d}^\top \mathbf{Z}^\top \hat{\mathbf{W}}\mathbf{Z}$ be the weighted projection matrix to project out the donor-level covariates with intercept, then $\tilde{\mathbf{G}}_\mathbf{d} = \mathbf{Z}(\mathbf{I} - \mathbf{Q}_{\tilde{\mathbf{X}}_\mathbf{d}})\mathbf{G}$, $E(\tilde{\mathbf{G}}_\mathbf{d}) = \mathbf{Z}(\mathbf{I} - \mathbf{Q}_{\tilde{\mathbf{X}}_\mathbf{d}})\mu_g\mathbf{1} = 0$, and $Cov(\tilde{\mathbf{G}}_\mathbf{d}) = \sigma_g^2\mathbf{Z}(\mathbf{I} - \mathbf{Q}_{\tilde{\mathbf{X}}_\mathbf{d}})\mathbf{\Psi}(\mathbf{I} - \mathbf{Q}_{\tilde{\mathbf{X}}_\mathbf{d}})^\top\mathbf{Z}^\top$.

For the denominator,

$$E(N^{-1}\tilde{\mathbf{G}}_\mathbf{d}^\top\hat{\mathbf{\Sigma}}^{-1}\tilde{\mathbf{G}}_\mathbf{d}) = \frac{\sigma_g^2}{N}tr\left(\hat{\mathbf{\Sigma}}^{-1}\mathbf{Z}(\mathbf{I} - \mathbf{Q}_{\tilde{\mathbf{X}}_\mathbf{d}})\mathbf{\Psi}(\mathbf{I} - \mathbf{Q}_{\tilde{\mathbf{X}}_\mathbf{d}})^\top\mathbf{Z}^\top\right),$$

Similarly, as the eigenvalues of $\hat{\mathbf{P}}, \hat{\mathbf{W}}, \mathbf{\Psi}, \hat{\mathbf{\Sigma}}^{-1}$ are bounded, and the distribution of $\mathbf{G}$ has bounded support, the variances of the numerator and the denominator terms are both $O(N^{-1})$, and the variance ratio converges to,

$$\hat{r}_s = \frac{\tilde{\mathbf{G}}^\top\hat{\mathbf{P}}\tilde{\mathbf{G}}}{\tilde{\mathbf{G}}_\mathbf{d}^\top\hat{\mathbf{\Sigma}}^{-1}\tilde{\mathbf{G}}_\mathbf{d}} \xrightarrow{p} \frac{lim_{N\to\infty}\left\{N^{-1}tr(\hat{\mathbf{P}}\mathbf{Z}\mathbf{\Psi}\mathbf{Z}^\mathbf{T})\right\}}{lim_{N\to\infty}\left\{N^{-1}tr\left(\hat{\mathbf{\Sigma}}^{-1}\mathbf{Z}(\mathbf{I} - \mathbf{Q}_{\tilde{\mathbf{X}}_\mathbf{d}})\mathbf{\Psi}(\mathbf{I} - \mathbf{Q}_{\tilde{\mathbf{X}}_\mathbf{d}})^\top\mathbf{Z}^\top\right)\right\}},$$

which is constant across all markers as the individual limits in the numerator and denominator exist and are bounded away from zero.

For each genetic variant, $\hat{r}$ is first used to approximate the variance $Var_{H_0}(T)$, if the p-value < 0.05, $\hat{r_s}$ is used to obtain a more accurate $Var_{H_0}(T)$.

3 Using saddlepoint approximation (SPA) for the null distribution of the score statistic

In traditional score tests, the distribution of the score statistic under H_0 is approximated by a Normal distribution, which uses the first two moments, mean and variance. This approach can perform poorly in the tail regions, especially if the underlying distribution is highly skewed when the studying event is very rare or the testing genetic variant has a very low minor allele count (MAC). Here, similar to what has been applied in the logistic mixed models [9, 10, 2, 11] previously, we use the SPA[12] to approximate the null distribution of the score statistic to obtain accurate p-values. For this, we utilize the fact that given the random effects $\boldsymbol{b}$, the phenotype Y, which are read counts from RNA sequencing, independently follow a Poisson distribution. $T_{adj} = \tilde{\mathbf{G}}^\top(\mathbf{y} - \hat{\mu})/\sqrt{\hat{r}\tilde{\mathbf{G}}^\top\hat{\mathbf{W}}\tilde{\mathbf{G}}}$ is a weighted sum of independent Poisson random variables. The approximated cumulant generating function (CGF) of T_{adj} is

$$K(\xi; \hat{\mu}, c) = \sum_{i=1}^{N}\hat{\mu_i}(e^{\tilde{G}_i c\xi} - \tilde{G}_i c\xi - 1),$$

where $c = (\tilde{G}^\top W\tilde{G})^{-1/2}$. $K'(\xi; \hat{\mu}, c)$ and $K''(\xi; \hat{\mu}, c)$ are the first and second derivatives of K with respect to ξ.

$$K'(\xi; \hat{\mu}, c) = \sum_{i=1}^{N}\hat{\mu}_i\tilde{G}_i c(e^{\tilde{G}_i c\xi} - 1),$$
$$K''(\xi; \hat{\mu}, c) = \sum_{i=1}^{N}\hat{\mu}_i\tilde{G}_i^2 c^2 e^{\tilde{G}_i c\xi}.$$

To calculate the probability that $T_{adj} < s$, where s is the observed test statistic, we use the following formula

$$Pr(T < s) = \Phi\left\{w + \frac{1}{w}log\left(\frac{v}{w}\right)\right\},$$

where $w = sign(\hat{\xi})[2\{\hat{\xi}s - K(\hat{\xi})\}]^{\frac{1}{2}}$, $v = \hat{\xi}[K''(\hat{\xi})]^{\frac{1}{2}}$, $\hat{\xi}$ is the solution of $K'(\hat{\xi}) = s$, and Φ is the standard normal distribution function.

4 Approaches to reduce computation and memory cost

We developed SAIGE-QTL based on the SAIGE framework, which utilizes several state-of-the-art approaches to reduce computation and memory cost to fit the null GLMM and to test for genetic associations. Given that in the single-cell RNA sequencing data, each individual may have hundreds and thousands of cells ($N >> n$), which N is the total number of cells and n is the total number of individuals, we use several approaches to make SAIGE-QTL conduct matrix operations on the scale of number of individuals to reduce a computation cost from $O(N)$ to $O(n)$.

First, to compute the test statistics $\mathbf{T} = \mathbf{G}^\top \mathbf{Z}^\top(\mathbf{y} - \hat{\mu})$ for each genetic variant, we pre-compute the statistics $\mathbf{Z}^\top(\mathbf{y} - \hat{\mu})$, so the computation cost of obtaining $\mathbf{T}$ is $O(n)$.

Second, to calculate $Var_{H_0}(T) = \tilde{\mathbf{G}}^\top \hat{\mathbf{P}} \tilde{\mathbf{G}}$ when estimating the variance ratios using randomly selected genetic markers, we need to compute quantities of the form $\hat{\mathbf{\Sigma}}^{-1}\mathbf{a}$, where $\mathbf{a}$ is a vector of length N. The standard computation technique of inverting $\hat{\mathbf{\Sigma}}$ (computation cost $O(N^3)$) and multiplying $\hat{\mathbf{\Sigma}}^{-1}$ with $\mathbf{a}$ can be extremely time consuming when N is large. By default, SAIGE-QTL computes $\hat{\mathbf{\Sigma}}^{-1}\mathbf{a}$ using the Sherman–Morrison–Woodbury[13, 14] (SMW) formula, which exploits the low-rank structure of $\hat{\mathbf{\Sigma}}$ to reduce this to an $n \times n$ donor-level computation with $O(nnz + n^2 + N)$ complexity (optimal $O(N)$ complexity for unrelated donors), where n is the number of donors and nnz is the number of non-zero elements in the $n \times n$ sparse genetic relationship matrix (GRM). When n is large and the memory usage of SMW is high, we instead implemented the pre-conditioned conjugate gradient[15] (PCG) method, which computes $\hat{\mathbf{\Sigma}}^{-1}\mathbf{a} = \mathbf{x}$ by iteratively solving the linear system of equations $\hat{\mathbf{\Sigma}}\mathbf{x} = \mathbf{a}$. Only $\hat{\mathbf{\Sigma}}\mathbf{a}$ needs to be computed when the PCG method is used. Instead of storing the $N \times N$ variance-covariance matrix $\mathbf{V}$(memory cost $O(N^2)$), we only store the K matrices $\mathbf{\Psi_k}$ with the size $n \times n$ (memory cost $O(kn^2)$) and the $N \times n$ design matrix $\mathbf{Z}$, which only cost $4N$ bytes if stored using a sparse matrix with 1s. Then, $\hat{\mathbf{\Sigma}}\mathbf{a}$ involves the computation for $\mathbf{Z}\mathbf{\Psi_k}\mathbf{Z}^\top\mathbf{a}$, which is computed consecutively, with the time cost O(Nnk). As implemented in SAIGE, we use the Hutchinson's randomized trace estimation[16, 17] for calculating $tr(\mathbf{PV})$. We also implemented multithreaded parallel computation for the matrix-vector multiplications in the PCG steps using Intel Threading Building Block (TBB) from the RcppParallel[18] package.

Third, as described in Section 2, in step 2, the variance of the score statistic for all markers can be approximated by $\hat{r}\mathbf{G_c}^\top \mathbf{Z}^\top \hat{\mathbf{W}} \mathbf{Z} \mathbf{G_c}$ or $\hat{r}_s \tilde{\mathbf{G}}_\mathbf{d}^\top \hat{\mathbf{\Sigma}}^{-1} \tilde{\mathbf{G}}_\mathbf{d}$. We pre-compute both $\mathbf{Z}^\top \hat{\mathbf{W}} \mathbf{Z}$ and $\mathbf{Z}^\top \hat{\mathbf{\Sigma}}^{-1} \mathbf{Z}$, so the computation to approximate the variance of the score statistic becomes $O(n)$ and for $\sim 5\%$ of genetic variants, it costs $O(n(p_d + 1))$.

Fourth, to reduce the redundancies of reading genotypes for each genetic variant when mapping eQTLs for all 20,000 genes, SAIGE-QTL allows for analyzing multiple genes simultaneously. We observed that computation time has been dropped dramatically compared to analyzing each gene separately. This is particularly useful for conducting genome-wide trans-eQTL mapping.

5 Overview of the SAIGE-QTL three-step framework

SAIGE-QTL maps *cis*-eQTLs in three steps (**Extended Data Fig. 1**). Step 1 iteratively estimates the null-model parameters using the penalized quasi-likelihood (PQL) method [19] and the computationally efficient average information restricted maximum likelihood (AI-REML) algorithm [4], also used in SAIGE [2] and GMMAT [1]. Inverting the $N \times N$ matrices required by a naive implementation costs $O(N^3)$, where N is the total number of cells, which can range from hundreds of thousands to millions. The Sherman–Morrison–Woodbury (SMW) formula [13, 14] exploits low-rank structure to transform this into an $n \times n$ donor-level system with $O(nnz + n^2 + N)$ complexity when $n \ll N$, where n is the number of donors and nnz is the number of non-zero elements in the $n \times n$ sparse genetic relationship matrix (GRM) for donors. For datasets with unrelated donors, for which the sparse GRM becomes the identity matrix, SMW further reduces to closed-form block-wise operations with optimal $O(N)$ complexity. When n is large and the memory usage of SMW is high, SAIGE-QTL provides an option to use the preconditioned conjugate gradient (PCG) approach [20], solving linear systems iteratively instead of inverting the $N \times N$ matrices directly, with $O(q_{iter}\, nnz + N)$ complexity, where q_{iter} is the number of iterations; for unrelated donors, PCG simplifies to $O(q_{iter}N)$ complexity as no GRM operation is required. Once the null model is fitted for a gene in a cell type, it can be used to test the association of any genetic marker or region with that gene's expression in that cell type.

In Step 2, for common variants, the score test is performed using model residuals and parameters estimated in Step 1. Computing the variance of the score statistic separately for all variants requires inverting an $N \times N$ matrix for each variant, which is prohibitively expensive. SAIGE-QTL instead uses a variance-ratio approximation derived under the Poisson mixed model, extending the

approach used in SAIGE and other GWAS methods [6, 5]. Two variance ratios are computed using a random subset of genetic markers, which have been shown to be approximately constant for variants with minor allele count (MAC) ≥ 20 for linear mixed models [6, 5] and generalized linear mixed models [2]: a ratio of the full variance of the score statistic to the variance without any random effects, and a ratio of the full variance to the variance with random effects (which is cheaper to compute than the full variance). Estimates of the second ratio have lower variation than the first, as previously demonstrated in SAIGE-GENE [8]. To improve computational efficiency while maintaining test accuracy, SAIGE-QTL first approximates the full variance using the ratio computed without random effects, similar to previous mixed-model GWAS methods, e.g., BOLT-LMM [6] and GRAMMAR-Gamma [5]. If the resulting association p-value is below a cutoff (e.g., 0.05), SAIGE-QTL re-estimates the full variance using the ratio computed with random effects and recalculates the p-value. SAIGE-QTL additionally approximates the null distribution of the score test statistic using the saddlepoint approximation (SPA) [12], as previously used in SAIGE [2], to obtain more accurate p-values than the Normal approximation (see Section 2 and the SPA section above for full derivations).

In Step 3, a gene-level p-value is obtained for each gene by combining the single-variant p-values from Step 2 across all variants in the *cis*-window using the ACAT-V test [21], based on the Cauchy combination method, as recently proposed for the linear mixed model eQTL mapping method APEX [22]. For M genetic variants in the *cis*-region with p-values p_m and weights v_m ($m = 1, \ldots, M$), let $T_{ACAT} = \sum_{m=1}^{M} v_m \tan\{(0.5 - p_m)\pi\}$ and $v = \sum_{m=1}^{M} v_m$; the ACAT-V p-value is then approximated by $p_{ACAT} \approx 1/2 - \{\arctan(T_{ACAT}/v)\}/\pi$. This Cauchy combination is computationally efficient and robust to correlation among the individual p-values [21], and provides highly concordant gene-level p-values to those obtained using the Beta approximation method used in FastQTL [23] and TensorQTL [24], while being substantially faster since it does not require permutations. ACAT-V also allows incorporating variant-level weights (e.g., distance to the transcription start site) to improve power to detect *cis*-eGenes.

6 Computational cost of SAIGE-QTL in OneK1K

The cost of Step 1 (fitting the null model with the default Sherman–Morrison–Woodbury setting) ranged between 32 and 1,239 CPU hours and 0.3 and 1.6 GB of memory usage across cell types, with differences driven largely by the number of cells per cell type (**Supplementary Table 2**). The projected overall computation time for Step 2, testing cis-eQTLs within a ± 1 Mb window around the gene body for all common variants (MAF $> 5\%$) across 20,000 genes, was between 28 and 40 CPU hours across the 14 cell types (estimated by projecting from genes expressed in at least 10% of donors; **Supplementary Table 3**).

We also evaluated the time cost of testing all common variation genome-wide for three representative cell types: CD4 naive and central memory T cells ("$CD4_{NC}$"), the most abundant cell type (463,528 cells); immature and naive B cells ("B_{IN}"), an intermediate cell type (82,068 cells); and Plasma, the least abundant cell type (3,625 cells). Testing 5.3 million genome-wide variants with MAF $> 5\%$ for 20,000 expressed genes was projected to take 630, 719 and 1,013 CPU hours for Plasma, B_{IN} and $CD4_{NC}$, respectively (**Supplementary Table 3**). Because each genetic marker can be tested for multiple genes within a single Step 2 job, the overhead of genotype reading is amortized across genes, substantially reducing computation time; parallel computation across multiple CPU cores further reduces runtime for jobs testing multiple genes.

Using PCG instead of SMW for matrix inversion in Step 1 was slightly more expensive, ranging between 34 and 1,569 CPU hours and 0.37 to 1.96 GB of memory usage. As the number of cells per donor increases ($n \ll N$), SMW is substantially faster than PCG: in a benchmark sampling ten times more cells from $CD4_{NC}$ (the original most abundant cell type) and testing ten randomly selected genes, Step 1 with SMW was approximately 10–20 times faster than with PCG, while producing numerically consistent results (**Extended Data Fig. 3**). SAIGE-QTL therefore defaults to SMW for Step 1, with PCG available as an alternative when SMW fails.

7 Calibration of SAIGE-QTL under permutation

We evaluated false-positive calibration using real OneK1K single-cell read counts with permuted donor genotypes, using the same three representative cell types ($CD4_{NC}$, B_{IN}, Plasma). Genes were binned into ten sparsity levels (in 10% increments, sparsity defined as the proportion of zero read counts), with up to 100 genes randomly sampled per bin per cell type (fewer if unavailable,

with more permutations to compensate; **Supplementary Table 1, 4**). For each gene, we fitted the null Poisson mixed model to the real read counts in Step 1, then permuted donor identities before testing 405,663 chromosome 1 variants with MAF $> 5\%$ in Step 2a, repeating the permutation 150 times, yielding at least 1.03×10^9 tests per gene at each sparsity level. Single-variant score tests were well calibrated across six significance thresholds from 5×10^{-7} to 0.05, with slight deflation for genes of medium-to-high sparsity and in less abundant cell types (Plasma and B_{IN}; **Supplementary Table 5, Extended Data Fig. 4**).

We similarly evaluated the gene-level ACAT-V test (Step 3), which combines single-variant p-values for common variants (MAF $> 5\%$) within each cis-region, using the same permutation design (100 genes per sparsity level per cell type, at least 2×10^7 tests per sparsity level per cell type). Gene-level ACAT-V tests were well controlled with a slight deflation, similar to the single-variant tests (**Supplementary Table 6, Extended Data Fig. 5**).

For rare variants (MAF $\leq 5\%$), we performed the set-based tests (Step 2b: Burden, SKAT [25], ACAT-V [21]) on chromosome 1 gene regions under 250 donor-label permutations, using two sets of variant weights (equal weights, and weights following a Beta(1,25) distribution, as commonly used in rare-variant association tests), generating approximately 8×10^4 null gene-based tests per sparsity level and cell type. This design allowed type I error assessment at significance thresholds down to 5×10^{-4}, and set-based tests were well calibrated across sparsity groups and cell types (**Extended Data Fig. 6**).

8 Comparison with existing single-cell eQTL methods

We compared SAIGE-QTL to two additional methods that model single-cell counts directly: the `glmer` function in the `lme4` R package, which fits a Poisson mixed model [26] and was previously used by Nathan et al. [27]; and the association test implemented in CellRegMap (CellRegMap-association) [28], which uses a linear mixed model. We benchmarked cis-eQTL mapping for 100 randomly selected genes in innate and naive B cells ("B_{IN}", 397,291 markers with MAF $> 5\%$).

SAIGE-QTL reduced computation time 244-fold relative to glmer (38 minutes for Step 1 and 16 minutes for Step 2 with SAIGE-QTL, versus 220 hours with glmer using one CPU). The two methods use the same Poisson mixed model but differ in test statistic: SAIGE-QTL uses a score test [29] whereas glmer uses a Wald test [30]. Comparing top hits per gene (using the SAIGE-QTL top hit when the two methods disagreed), the two methods produced highly concordant results (Pearson $R^2 = 0.991$ for $-\log_{10}(P)$ values and 0.998 for estimated effect sizes; **Supplementary Table 9, Supplementary Figure 8**). The Wald test in glmer tends to produce more significant p-values than the score tests used by SAIGE-QTL and CellRegMap-association because it fits the full mixed-effects model directly, typically yielding more accurate effect-size and variance estimates, at substantially higher computational cost; users can refine effect-size and p-value estimates for eGenes of interest by refitting the full model with genotype included as a covariate in Step 1 of SAIGE-QTL.

CellRegMap-association is implemented within CellRegMap, which was designed primarily for dynamic eQTL mapping rather than optimized for standard association testing, so its computational performance is not directly comparable to SAIGE-QTL. SAIGE-QTL nonetheless produced highly concordant results with CellRegMap-association (Pearson $R^2 = 0.982$ for $-\log_{10}(P)$ values). Other single-cell eQTL methods, such as GASPACHO and CellRegMap's interaction test, target a different goal (identifying eQTLs that vary along continuous cell states or trajectories) and are therefore not directly comparable to SAIGE-QTL's current goal of population-level eQTL discovery.

Compared with these methods, SAIGE-QTL additionally supports set-based tests for rare-variant eQTL mapping. In the same 100-gene benchmark (480–3,812 rare variants per gene, MAF $< 5\%$, mean $\approx$ 1,489), set-based tests in B_{IN} cells required $\sim$23 minutes of runtime and 1.7 GB of memory using a single CPU; extrapolating, rare-variant eQTL mapping for $\sim$22,000 genes in B_{IN} cells is estimated to require $\sim$85 CPU hours.

9 Comparison with pseudobulk eQTL methods: cost, calibration and power

We compared SAIGE-QTL with two pseudobulk eQTL methods: TensorQTL [24], which applies linear regression to pseudobulk (donor-mean) expression after rank-based inverse-normal transformation of the phenotype, and jaxQTL [31], which applies negative-binomial regression (as recom-

mended) to pseudobulk counts obtained by summing single-cell read counts across donors within each cell type. We compared computational cost, type I error (TIE) rates, and power to detect eQTLs.

Benchmarking cis-eQTL mapping for the same 100 randomly selected genes in B_{IN} cells (single CPU, Linux HPC cluster, dual Intel Xeon Gold 6230 CPUs at 2.10 GHz, AMD64 architecture), TensorQTL and jaxQTL were approximately 4.4- and 4.0-fold faster than SAIGE-QTL, respectively, as expected since SAIGE-QTL models read counts directly from cells ($N = 82{,}068$) whereas the pseudobulk methods operate on donor-collapsed counts ($n = 982$). Despite its higher runtime, SAIGE-QTL was more memory-efficient, requiring less than 1 GB compared with 6.3 GB for TensorQTL and 36.5 GB for jaxQTL (**Supplementary Table 9**).

We estimated TIE rates for all three methods using real OneK1K read counts with permuted genotypes, across genes at four sparsity levels (5%, 25%, 75% and 95% cell-level nonzero expression), even and uneven per-donor cell counts (the uneven design preserving OneK1K's empirical donor-specific cell counts; the even design equalizing cell counts at the cell type's median empirical value via within-donor resampling), and the three representative cell types ($CD4_{NC}$, B_{IN}, Plasma). Per-cell read counts were generated from a Poisson mixed-effects model, $\log(\lambda_{ij}) = \log(L_{ij}) + \mu + b_i$ with $y_{ij} \sim \text{Poisson}(\lambda_{ij})$, where L_{ij} is the observed total read count for cell j of donor i and $b_i \sim N(0, 0.2)$ is a donor-specific random effect; the intercept μ was calibrated via a binary search so that the empirical proportion of nonzero counts matched each target sparsity level. Each scenario was replicated across 500 genes and 2,100 LD-pruned SNPs, allowing type I error estimation down to a significance level of 5×10^{-5}. SAIGE-QTL and TensorQTL had well-controlled TIE rates across significance thresholds from 5×10^{-2} to 5×10^{-5} under all scenarios, while jaxQTL showed modest inflation that increased at more stringent thresholds (e.g., $\alpha \le 5 \times 10^{-4}$; **Supplementary Table 10, Supplementary Figure 9**).

We then compared empirical power using the same simulation scenarios, extending the phenotype model to $\log(\lambda_{ij}) = \log(L_{ij}) + \mu + \beta_g G_i + b_i$ with a single causal variant (MAF = 0.2) and varying effect sizes β_g (0.05 to 3). Because jaxQTL's type I error was inflated, we estimated power at each method's test-specific empirical α yielding a true type I error rate between 5×10^{-2} and 5×10^{-5}, so inflated tests were compared at more stringent significance thresholds. SAIGE-QTL maintained the highest empirical power across all scenarios, with the power advantage over the two pseudobulk methods largest in less abundant cell types (e.g., Plasma; **Supplementary Table 11, Supplementary Figure 10**).

By modeling read counts at the single-cell level, SAIGE-QTL directly accounts for both donor-level and cell-level covariates (e.g., cell state) within the Poisson mixed-model framework. Pseudobulk tools instead must first aggregate cell-level covariates across all cells from a donor before including them as donor-level covariates, which is suboptimal for capturing cell-level covariate effects and can reduce power. Pseudobulk approaches also summarize both expression and covariates to the donor level, failing to account for differing numbers of cells sequenced per donor: for example, a donor-level mean estimated from 1,000 cells has substantially lower uncertainty than one estimated from only 10 cells, a distinction pseudobulk methods do not model explicitly. By modeling cell-level covariates and within-donor cell-to-cell correlation directly, SAIGE-QTL is more robust to uneven cell counts across donors.

To quantify this, we simulated 4,000 additional B_{IN}-cell-type gene-expression datasets, with each condition (i.e., each combination of parameter values shown along the x axis) evaluated using $n = 500$ simulated genes, varying (i) per-donor cell counts, drawn from a Gamma distribution with shape parameter γ and scale equal to the empirical mean number of cells per donor divided by γ (such that $Var(n_i) =$ mean cells per donor$^2/\gamma$), spanning even (large γ) to uneven (small γ) sampling intensity across donors, and (ii) the effect size of an added cell-level covariate, $\beta_{cov} C_{ij}$ with $C_{ij} \sim N(0, 0.5)$, ranging from low to high. As cell counts became more uneven and cell-level covariate effects increased, the power advantage of SAIGE-QTL over TensorQTL and jaxQTL became more pronounced (**Supplementary Figure 11**), confirming the benefit of directly modeling gene expression counts and covariates at the single-cell level.

References

[1] Han Chen, Chaolong Wang, Matthew P Conomos, Adrienne M Stilp, Zilin Li, Tamar Sofer, Adam A Szpiro, Wei Chen, John M Brehm, Juan C Celedón, Susan Redline, George J Papanicolaou, and Timothy A Thornton. Control for population structure and relatedness for binary traits in genetic association studies via logistic mixed models. *The American Journal of Human Genetics*, 98(4):653–666, 2016.

[2] Wei Zhou, Jonas B Nielsen, Lars G Fritsche, Rounak Dey, Maiken E Gabrielsen, Brooke N Wolford, Jonathon Lefaive, Peter Vandehaar, Sarah A Gagliano, Aliya Gifford, Lisa A Bastarache, Wei-Qi Wei, Joshua C Denny, Maoxuan Lin, Kristian Hveem, Hyun Min Kang, Goncalo R Abecasis, Cristen J Willer, and Seunggeun Lee. Efficiently controlling for case-control imbalance and sample relatedness in large-scale genetic association studies. *Nature genetics*, 50(9):1335–1341, 2018.

[3] Rounak Dey, Wei Zhou, Tuomo Kiiskinen, Aki Havulinna, Amanda Elliott, Juha Karjalainen, Mitja Kurki, Ashley Qin, FinnGen, Seunggeun Lee, et al. An efficient and accurate frailty model approach for genome-wide survival association analysis controlling for population structure and relatedness in large-scale biobanks. *bioRxiv*, pages 2020–10, 2020.

[4] Arthur R Gilmour, Robin Thompson, and Brian R Cullis. Average information REML: An efficient algorithm for variance parameter estimation in linear mixed models. *Biometrics*, 51(4):1440–1450, 1995.

[5] Gulnara R Svishcheva, Tatiana I Axenovich, Nadezhda M Belonogova, Cornelia M Van Duijn, and Yurii S Aulchenko. Rapid variance components–based method for whole-genome association analysis. *Nature Genetics*, 44(10):1166, 2012.

[6] Po-Ru Loh, George Tucker, Brendan K Bulik-Sullivan, Bjarni J Vilhjálmsson, Hilary K Finucane, Rany M Salem, Daniel I Chasman, Paul M Ridker, Benjamin M Neale, Bonnie Berger, Nick Patterson, and Alkes L Price. Efficient bayesian mixed-model analysis increases association power in large cohorts. *Nature genetics*, 47(3):284, 2015.

[7] Longda Jiang, Zhili Zheng, Ting Qi, Kathryn Kemper, Naomi Wray, Peter Visscher, and Jian Yang. A resource-efficient tool for mixed model association analysis of large-scale data. *Nature Genetics*, 51(12):1749–2, 2019.

[8] Wei Zhou, Zhangchen Zhao, Jonas B Nielsen, Lars G Fritsche, Jonathon LeFaive, Sarah A Gagliano Taliun, Wenjian Bi, Maiken E Gabrielsen, Mark J Daly, Benjamin M Neale, et al. Scalable generalized linear mixed model for region-based association tests in large biobanks and cohorts. *Nature genetics*, 52(6):634–639, 2020.

[9] Rounak Dey, Ellen M Schmidt, Goncalo R Abecasis, and Seunggeun Lee. A fast and accurate algorithm to test for binary phenotypes and its application to phewas. *The American Journal of Human Genetics*, 101(1):37–49, 2017.

[10] Rounak Dey, Jonas B. Nielsen, Lars G. Fritsche, Wei Zhou, Huanhuan Zhu, Cristen J. Willer, and Seunggeun Lee. Robust meta-analysis of biobank-based genome-wide association studies with unbalanced binary phenotypes. *Genetic Epidemiology*, 43(5):462–476, 2019.

[11] Wenjian Bi, Zhangchen Zhao, Rounak Dey, Lars G Fritsche, Bhramar Mukherjee, and Seunggeun Lee. A fast and accurate method for genome-wide scale phenome-wide G x E analysis and its application to UK Biobank. *The American Journal of Human Genetics*, 105(6):1182–1192, 2019.

[12] H. E. Daniels. Saddlepoint approximations in statistics. *The Annals of Mathematical Statistics*, 25(4):631–650, 1954.

[13] J. Sherman and W. J. Morrison. Adjustment of an inverse matrix corresponding to a change in one element of a given matrix. *Annals of Mathematical Statistics*, 21:124–127, 1950.

[14] M. A. Woodbury. Inverting modified matrices. Technical Report Memorandum Report 42, Statistical Research Group, Princeton University, 1950.

[15] S Tsuruta, I Misztal, and I Strandén. Use of the preconditioned conjugate gradient algorithm as a generic solver for mixed-model equations in animal breeding applications. *Journal of animal science*, 79(5):1166–1172, 2001.

[16] M.F. Hutchinson. A stochastic estimator of the trace of the influence matrix for laplacian smoothing splines. *Communications in Statistics - Simulation and Computation*, 19(2):433–450, 1990.

[17] Haim Avron and Sivan Toledo. Randomized algorithms for estimating the trace of an implicit symmetric positive semi-definite matrix. *Journal of the ACM (JACM)*, 58(2):1–34, 2011.

[18] JJ Allaire, Romain Francois, Kevin Ushey, Gregory Vandenbrouck, Marcus Geelnard, and Intel. *RcppParallel: Parallel Programming Tools for 'Rcpp'*, 2019. R package version 4.4.4.

[19] N. E. Breslow and D. G. Clayton. Approximate inference in generalized linear mixed models. *Journal of the American Statistical Association*, 88:9–25, 1993.

[20] E. F. Kaasschieter. Preconditioned conjugate gradients for solving singular systems. *Journal of Computational and Applied Mathematics*, 24:265–275, 1988.

[21] Y. Liu, S. Chen, Z. Li, A. C. Morrison, E. Boerwinkle, and X. Lin. Acat: A fast and powerful p value combination method for rare-variant analysis in sequencing studies. *American Journal of Human Genetics*, 104:410–421, 2019.

[22] C. Quick, X. Wen, G. Abecasis, M. Boehnke, and H. M. Kang. A versatile toolkit for molecular qtl mapping and meta-analysis at scale. *bioRxiv*, 2020.

[23] H. Ongen, A. Buil, A. A. Brown, E. T. Dermitzakis, and O. Delaneau. Fast and efficient qtl mapper for thousands of molecular phenotypes. *Bioinformatics*, 32:1479–1485, 2016.

[24] A. Taylor-Weiner, F. Aguet, N. J. Haradhvala, S. Gosai, S. Anand, J. Kim, K. Ardlie, E. M. Van Allen, and G. Getz. Scaling computational genomics to millions of individuals with gpus. *Genome Biology*, 20:228, 2019.

[25] M. C. Wu, S. Lee, T. Cai, Y. Li, M. Boehnke, and X. Lin. Rare-variant association testing for sequencing data with the sequence kernel association test. *American Journal of Human Genetics*, 89:82–93, 2011.

[26] D. Bates, M. Mächler, B. Bolker, and S. Walker. Fitting linear mixed-effects models using lme4. *Journal of Statistical Software*, 67:1–48, 2015.

[27] A. Nathan, S. Asgari, K. Ishigaki, C. Valencia, T. Amariuta, Y. Luo, J. I. Beynor, Y. Baglaenko, S. Suliman, A. L. Price, M. Lecca, M. Alcalde-Herraiz, J. B. Kang, S. Chun, K. Slowikowski, F. Zhang, E. M. Behrens, S. Prahalad, V. P. Bykerk, S. M. Goodman, A. B. Pernis, L. B. Ivashkiv, K. Wei, J. A. Lederer, S. M. Fortune, D. A. Rao, and S. Raychaudhuri. Single-cell eqtl models reveal dynamic t cell state dependence of disease loci. *Nature*, 606:120–128, 2022.

[28] A. S. E. Cuomo, T. Heinen, D. Vagiaki, D. Horta, J. C. Marioni, and O. Stegle. Cellregmap: a statistical framework for mapping context-specific regulatory variants using scrna-seq. *Molecular Systems Biology*, 18:e10663, 2022.

[29] C. Radhakrishna Rao. Large sample tests of statistical hypotheses concerning several parameters with applications to problems of estimation. *Mathematical Proceedings of the Cambridge Philosophical Society*, 44:50–57, 1948.

[30] A. Wald. Tests of statistical hypotheses concerning several parameters when the number of observations is large. *Transactions of the American Mathematical Society*, 54:426–482, 1943.

[31] Z. E. Zhang, A. Kim, N. Suboc, N. Mancuso, and S. Gazal. Efficient count-based models improve power and robustness for large-scale single-cell eqtl mapping. *medRxiv*, 2025.
