# Supplementary material for: Efficient and accurate mixed model association tool for single-cell eQTL analysis
Source: Nat Genet. 2026 Nov 6;58(11):2984–92. doi: 10.1038/s41588-026-02798-2 (PMC13754595; doi:10.1038/s41588-026-02798-2)
Supplement: Supplementary file 2 [file 41588_2026_2798_MOESM2_ESM.pdf]

nature portfolio

Corresponding author(s): Wei Zhou, Benjamin Neale

Last updated by author(s): 08/17/2026

Reporting Summary

Nature Portfolio wishes to improve the reproducibility of the work that we publish. This form provides structure for consistency and transparency in reporting. For further information on Nature Portfolio policies, see our Editorial Policies and the Editorial Policy Checklist.

Statistics

For all statistical analyses, confirm that the following items are present in the figure legend, table legend, main text, or Methods section.

n/a	Confirmed	
☐	☒	The exact sample size (*n*) for each experimental group/condition, given as a discrete number and unit of measurement
☐	☒	A statement on whether measurements were taken from distinct samples or whether the same sample was measured repeatedly
☐	☒	The statistical test(s) used AND whether they are one- or two-sided<br>*Only common tests should be described solely by name; describe more complex techniques in the Methods section.*
☐	☒	A description of all covariates tested
☐	☒	A description of any assumptions or corrections, such as tests of normality and adjustment for multiple comparisons
☐	☒	A full description of the statistical parameters including central tendency (e.g. means) or other basic estimates (e.g. regression coefficient) AND variation (e.g. standard deviation) or associated estimates of uncertainty (e.g. confidence intervals)
☐	☒	For null hypothesis testing, the test statistic (e.g. *F*, *t*, *r*) with confidence intervals, effect sizes, degrees of freedom and *P* value noted<br>*Give P values as exact values whenever suitable.*
☒	☐	For Bayesian analysis, information on the choice of priors and Markov chain Monte Carlo settings
☐	☒	For hierarchical and complex designs, identification of the appropriate level for tests and full reporting of outcomes
☐	☒	Estimates of effect sizes (e.g. Cohen's *d*, Pearson's *r*), indicating how they were calculated

Our web collection on statistics for biologists contains articles on many of the points above.

Software and code

Policy information about availability of computer code

Data collection	This research has been conducted using the OneK1K single-cell RNA-seq and genotype data, which are available via Gene Expression Omnibus (GSE196830)
Data analysis	SAIGE-QTL is implemented as an open-source R method available at:<br>https://github.com/weizhou0/SAIGEQTL<br>Code to reproduce all analyses and figures included here can be found at: https://github.com/annacuomo/SAIGE_QTL_analyses<br>Both the SAIGE-QTL software and the analysis code have been deposited in Zenodo https://doi.org/10.5281/zenodo.21980414<br><br>The following tools were run in the manuscript:<br>Single-cell RNA seq eQTL mapping tools:<br>SAIGE-QTL: version 0.3.5<br>CellRegMap: using docker image annasecuomo/cellregmap:v0.0.3<br><br>Pseudobulk eQTL mapping tools:<br>jaxQTL: version number 0.1.dev366+ge29734071<br>TensorQTL: v1.0.10

For manuscripts utilizing custom algorithms or software that are central to the research but not yet described in published literature, software must be made available to editors and reviewers. We strongly encourage code deposition in a community repository (e.g. GitHub). See the Nature Portfolio guidelines for submitting code & software for further information.

Data

Policy information about availability of data

All manuscripts must include a data availability statement. This statement should provide the following information, where applicable:

- Accession codes, unique identifiers, or web links for publicly available datasets
- A description of any restrictions on data availability
- For clinical datasets or third party data, please ensure that the statement adheres to our policy

The OneK1K single-cell RNA-seq and genotype data were available via Gene Expression Omnibus (GSE196830). Summary statistics for the eQTLs in this study can be found on Zenodo: https://zenodo.org/records/10811106. Source data for figures are deposited on Zenodo: https://zenodo.org/records/21987029

GWAS summary stats for the MR analysis were downloaded from: i) CD and IBD GWAS were downloaded from IBD Genetics Consortium website (www.ibdgenetics.org) from Liu et al. Nature Genetics. 2015; ii) RA GWAS summary was downloaded from GWAS Catalog (study ID: GCST90132223) in Ishigaki et al. Nature Genetics, 2022 (https://ftp.ebi.ac.uk/pub/databases/gwas/summary_statistics/GCST90132001-GCST90133000/GCST90132223/GCST90132223_buildGRCh37.tsv.gz); iii) SLE GWAS summary was downloaded from GWAS Catalog (study ID: GCST90018917) in Sakaue et al. Nature Genetics, 2021 (https://ftp.ebi.ac.uk/pub/databases/gwas/summary_statistics/GCST90018001-GCST90019000/GCST90018917/GCST90018917_buildGRCh37.tsv.gz).

Research involving human participants, their data, or biological material

Policy information about studies with human participants or human data. See also policy information about sex, gender (identity/presentation), and sexual orientation and race, ethnicity and racism.

Reporting on sex and gender	In realdata analysis and simulation studies, individuals of all sexes were included. Sex was included as covariates.
Reporting on race, ethnicity, or other socially relevant groupings	We analyzed the OneK1K data set published in Yazar et al., 2022, which consists of 982 individuals of Northern European ancestry. We used the top six genetic principal components as covariates in the analyses.
Population characteristics	We included information on age, sex, six genetic principal components, two peer factors as covariates in the analyses. Genotype data was obtained from the OneK1K project.
Recruitment	We did not recruit participants for this study.
Ethics oversight	The OneK1K single-cell RNA-seq and genotype data were available via Gene Expression Omnibus (GSE196830).

Note that full information on the approval of the study protocol must also be provided in the manuscript.

Field-specific reporting

Please select the one below that is the best fit for your research. If you are not sure, read the appropriate sections before making your selection.

☒ Life sciences ☐ Behavioural & social sciences ☐ Ecological, evolutionary & environmental sciences

For a reference copy of the document with all sections, see nature.com/documents/nr-reporting-summary-flat.pdf

Life sciences study design

All studies must disclose on these points even when the disclosure is negative.

Sample size	For real data analysis, we used scRNA-seq data from 14 different immune cell types for 1,267,758 peripheral blood mononuclear cells (PBMCs) and 982 individuals of European ancestry from the Tasmanian Ophthalmic Biobank in Tasmania, Australia as reported in Yazar et al. Nature Genetics 2022.
Data exclusions	For eQTL analysis in each cell type, we excluded genes that are expressed in less than 10% of the donors.
Replication	To replicate the improved performance of SAIGE-QTL over the pseudobulk approach, we used the scRNA-seq data from 91 European ancestry individuals in the California Lupus Epidemiology Study as described in Perez et al., 2022 and 110 unique iPSC lines reprogrammed from fibroblasts of patients with primary open-angle glaucoma (POAG), categorized into six distinct cell types from 52 to 110 donors as described in Daniszewski et al., 2022.
Randomization	To evaluate the calibration of SAIGE-QTL across cell type abundances, gene expression levels (measured in terms of sparsity, defined as the proportion of zero read counts), we considered the three cell types described above and randomly selected up to 100 genes for each of the 10 sparsity levels (ranging in 10% increments from 0% to 100%) in each cell type. After fitting null model for each gene, we randomly permuted genotypes across donors.
Blinding	Blinding was not applicable to the analyses we performed within the OneK1K project which analyzed all samples jointly.

Reporting for specific materials, systems and methods

We require information from authors about some types of materials, experimental systems and methods used in many studies. Here, indicate whether each material, system or method listed is relevant to your study. If you are not sure if a list item applies to your research, read the appropriate section before selecting a response.

Materials & experimental systems

n/a	Involved in the study
☒	☐ Antibodies
☒	☐ Eukaryotic cell lines
☒	☐ Palaeontology and archaeology
☒	☐ Animals and other organisms
☒	☐ Clinical data
☒	☐ Dual use research of concern
☒	☐ Plants

Methods

n/a	Involved in the study
☒	☐ ChIP-seq
☒	☐ Flow cytometry
☒	☐ MRI-based neuroimaging

Plants

Seed stocks	Not applicable
Novel plant genotypes	Not applicable
Authentication	Not applicable
