# Supplementary material for: Efficient and accurate mixed model association tool for single-cell eQTL analysis
Source: Nat Genet. 2026 Nov 6;58(11):2984–92. doi: 10.1038/s41588-026-02798-2 (PMC13754595; doi:10.1038/s41588-026-02798-2)
Supplement: Supplementary file 3 [file 41588_2026_2798_MOESM3_ESM.pdf]

nature portfolio

Peer Review File

Efficient and accurate mixed model association tool for single-cell eQTL analysis

Corresponding Author: Dr Wei Zhou

Version 0:

Decision Letter:

2nd Jul 2024

Dear Dr Zhou,

Thank you for submitting your manuscript entitled "Efficient and accurate mixed model association tool for single-cell eQTL analysis", for consideration. I regret that we are unable to publish it in Nature Genetics.

As you may know, we decline a substantial proportion of manuscripts without sending them to referees, so that they may be sent elsewhere without delay. Even if referees were to find your manuscript to be technically correct, we are concerned that the work is too preliminary to warrant publication in Nature Genetics.

In brief, we acknowledge the potentially very high impact of an improved method for sc-eQTL identification. However, there are two major shortcomings in the current version of the work that we think absolutely must be addressed before a potential decision on peer review at NG.

Firstly, the benchmarking is very limited, against tensorQTL pseudobulk only; this ignores that there are multiple sc-eQTL methods out there, such as Poisson mixed effect models, CellRegMap, and GASPACHO (published at the journal). Relatedly, the simulations as currently performed are quite circumscribed (only a small # genes, low/medium/high abundance cell types, with varying sparsity), and should be expanded to be much more comprehensive.
Secondly, the application to a single (OneK1K) dataset is limiting, as it is of a single tissue type, while the inferred sc-eQTLs are not validated in a further cohort, meaning it is impossible to judge whether these are real or potentially artefactual.

We know from long experience that referees inevitably demand these if lacking, and thus we are reluctant to proceed to peer review without such an improvement.

Should future data, experiments and analysis allow you to appeal our decision, we would be happy to look at a new submission (unless, of course, something similar has by then been accepted at Nature Genetics or appeared elsewhere). This includes submission or publication of a portion of this work someplace else. In the case of eventual publication, the received date would be that of the revised manuscript.

If you are interested in submitting a suitably revised manuscript in the future or if you have any questions, please contact me. Otherwise, we wish you the best in finding a suitable home for your manuscript.

Thank you for your interest in Nature Genetics. I am sorry that on this occasion we cannot be more positive.

Sincerely,

Michael Fletcher, PhD
Senior Editor, Nature Genetics
ORCiD: 0000-0003-1589-7087

Version 1:

Decision Letter:

IMPORTANT: Please note the reference number: NG-TR65894R-Z Zhou. This number must be quoted whenever you communicate with us regarding this paper.

24th Jan 2025

Dear Wei,

Thank you for your message asking us to reconsider our decision on your manuscript "Efficient and accurate mixed model association tool for single-cell eQTL analysis".

I have now discussed the points of your letter with my colleagues, and we think that your changes have addressed the issues we flagged in our initial pre-review decision. We therefore invite you to resubmit your revised manuscript for peer review.

When preparing a revision, please ensure that it fully complies with our editorial requirements for format and style; details can be found in the Guide to Authors on our website (http://www.nature.com/ng/).

Please be sure that your manuscript is accompanied by a separate letter detailing the changes you have made and your response to the points raised. At this stage we will need you to upload:
1) a copy of the manuscript in MS Word .docx format.
2) The Editorial Policy Checklist:
https://www.nature.com/documents/nr-editorial-policy-checklist.pdf
3) The Reporting Summary:
https://www.nature.com/documents/nr-reporting-summary.pdf
(Here you can read about the role of the Reporting Summary in reproducible science:
https://www.nature.com/news/announcement-towards-greater-reproducibility-for-life-sciences-research-in-nature-1.22062)

Please use the link below to be taken directly to the site and view and revise your manuscript:

Link Redacted

With kind wishes,

Michael Fletcher, PhD
Senior Editor, Nature Genetics
ORCiD: 0000-0003-1589-7087

Version 2:

Decision Letter:

21st October 2025

Dear Wei,

Your Technical Report "Efficient and accurate mixed model association tool for single-cell eQTL analysis" has been seen by two referees. (Reviewer #2 accepted the original review request but has not returned comments). You will see from the comments below that, while Reviewers #1 and #3 find your work of potential interest, they have raised several relevant concerns. We are interested in the possibility of publishing your study in Nature Genetics, but we would like to consider your response to these concerns in the form of a revised manuscript before we make a final decision on publication.

To guide the scope of the revisions, the editors discuss the referee reports in detail within the team, including with the chief editor, with a view to identifying key priorities that should be addressed in revision, and sometimes overruling referee requests that are deemed beyond the scope of the current study. In this case, we particularly ask that you address all technical concerns related to the software package and its implementation, perform additional benchmarking studies, and extend the analyses where feasible as requested by the reviewers. We hope you will find this prioritized set of referee points to be useful when revising your study. Please do not hesitate to get in touch if you would like to discuss these issues further.

We therefore invite you to revise your manuscript taking into account all reviewer and editor comments. Please highlight all changes in the manuscript text file. At this stage, we will need you to upload a copy of the manuscript in MS Word .docx or similar editable format.

We are committed to providing a fair and constructive peer-review process. Do not hesitate to contact us if there are specific requests from the reviewers that you believe are technically impossible or unlikely to yield a meaningful outcome.

When revising your manuscript:

*1) Include a “Response to referees” document detailing, point-by-point, how you addressed each referee comment. If no action was taken to address a point, you must provide a compelling argument. This response will be sent back to the referees along with the revised manuscript.

*2) If you have not done so already, please begin to revise your manuscript so that it conforms to our Technical Report format instructions, available
here.
Refer also to any guidelines provided in this letter.

*3) Include a revised version of your Reporting Summary: https://www.nature.com/documents/nr-reporting-summary.pdf
It will be available to referees (and, potentially, statisticians) to aid in their evaluation if the manuscript goes back for peer review.
A revised checklist is essential for re-review of the paper.

Please be aware of our guidelines on digital image standards.

EXTENDED DATA FIGURES

When re-submitting your manuscript, please ensure that any supplementary figures and tables that are crucial to the manuscript’s conclusions are converted into Extended Data figures and tables to increase visibility of these data. Extended Data figures and tables are online-only (present in the online PDF and full-text HTML versions of the paper), peer-reviewed display items that provide essential background to the article but are not included in the main article due to space constraints. A maximum of ten Extended Data display items (figures and tables) is permitted.

Please use the link below to submit your revised manuscript and related files:

Link Redacted

Note: This URL links to your confidential home page and associated information about manuscripts you may have submitted, or that you are reviewing for us. If you wish to forward this email to co-authors, please delete the link to your homepage.

We hope to receive your revised manuscript within 8-12 weeks. If you cannot send it within this time, please let us know.

Please do not hesitate to contact me if you have any questions or would like to discuss these revisions further.

Nature Genetics is committed to improving transparency in authorship. As part of our efforts in this direction, we are now requesting that all authors identified as ‘corresponding author’ on published papers create and link their Open Researcher and Contributor Identifier (ORCID) with their account on the Manuscript Tracking System (MTS), prior to acceptance. ORCID helps the scientific community achieve unambiguous attribution of all scholarly contributions. You can create and link your ORCID from the home page of the MTS by clicking on ‘Modify my Springer Nature account’. For more information, please visit www.springernature.com/orcid.

We look forward to seeing the revised manuscript and thank you for the opportunity to review your work.

Sincerely,
Kyle

Kyle Vogan, PhD
Senior Editor
Nature Genetics
https://orcid.org/0000-0001-9565-9665

Referee expertise:

Referee #1: Genetics, molecular QTLs, single-cell analyses

Referee #3: Genetics, molecular QTLs, single-cell analyses

Reviewers' Comments:

Reviewer #1 (Remarks to the Author):

Zhou et al. presented a computational tool for mapping single-cell eQTL at scale and showed the improvement of statistical power over the 'pseudobulk' approach implemented in TensorQTL. However, compared to existing single-cell eQTL methods, there were no significant improvements in the statistical power but computational speed. In general, there is lack of novelty in methodology development (such as rare variant association). To speed up the tool, the authors did not consider the GPU-based acceleration, which represents the current trend of new genomic tools development. There is still lack of comprehensive comparisons between SAIGE-QTL and existing both 'pseudobulk' and single-cell eQTL mapping methods in the simulated datasets.

In abstract: It would be good to provide some details about the improvements of SAIGE-QTL compared to existing 'pseudobulk' and single-cell eQTL tools. In addition, please describe the novel biological findings delivered by SAIGE-QTL when applying it to real data.

In the computation and memory cost: Please compare SAIGE-QTL with other existing methods, including TensorQTL. Please report the computation cost of set-based test for rare variants.

Line 223: Not sure if 150 times permutation was enough. Please justify.

Line 216 and 228: Did the authors consider heritability of gene expression when selecting genes? It would be good to compare SAIGE-QTL with other existing methods across all the simulated scenarios. In which scenarios did SAIGE-QTL outperform the 'pseudobulk' approach?

Line 246: Why 250 times permutation? Please justify.

Line 348-357: It would be good to compare SAIGE-QTL with commonly used GWAS methods (e.g., GCTA) in trans-eQTL mapping.

Line 359-375: It would be good if the authors could biologically interpret the findings from rare-variant eQTL mapping. Will these results help us better understand the genetics of complex phenotypes?

Line 411: please justify why not compare SAIGE-QTL with other single-cell eQTL methods in these two datasets?

Reviewer #3 (Remarks to the Author):

Zhou et al introduce introduce a new method and software tool, SAIGE-QTL, for mapping expression quantitative trait loci from single-cell RNA-sequencing data. The paper has great potential to be an important contribution to the field, with important new methodologies that open up exciting new possibilities for single-cell molecular QTL mapping.

However, my attempts to complete a thorough review of the work were stymied by persistent failures in installing (the R package) or running (via the Docker image) the software (all replicated by a senior postdoc in my group). As such, I provide my initial comments and feedback here, but would need engage with a revision that provides easily installable and runnable software for SAIGE-QTL to evaluate the paper and method sufficiently to the degree required for publication in Nature Genetics.

I would be very happy to review this paper again when provided with a working version of the software that I can install, run, and test out for myself as despite my current frustrations with the software implementation I can see that this is work of potentially high impact and importance.

Key contributions of this work:

- feasible eQTL mapping using single-cell resolution data (not requiring pseudobulking)
- speed and scalability of the method relative to existing options
- set tests to map effects of rare variants
- extension of SAIGE (Scalable and Accurate Implementation of GEneralized mixed model), SAIGE-GENE and SAIGE-GENE+ to eQTL mapping
- enables trans-eQTL mapping

Method:

1. Fit null Poisson MM, which includes individual-level covariates, such as sex, age, and ancestry PCs for donors, and cell-specific covariates, such as expression-based PEER factors, as fixed effects and accounts for intra-individual cell-to-cell variability using a random effect
2. a) Testing for the association between each common genetic variant (e.g., MAF > 5%) and gene expression using score tests

b) conducting set-based tests for less frequent/rare variants with e.g., MAF < 5%, including Burden tests, SKAT, and ACAT-V, each having superior power in various scenarios of effect directions and sparsity levels followed by an ACAT-O test to combine p-values (see 10.1016/j.ajhg.2019.01.002)
3. calculating a gene-level p-value for each cis-eQTL region using the ACAT-V test, which combines variant-level p-values (from Step 2a) using a Cauchy combination that is robust to the correlation among individual p-values and the sparsity of effects

Step 1 iteratively estimates the model parameters using the penalised quasi-likelihood (PQL) method and the computationally efficient average information restricted maximum likelihood algorithm. Once the null model is fitted for a gene in a cell type, it can be used to test the association of any genetic markers or regions for that gene's expression in that cell type.

In Step 2, for common variants, the score test is performed based on model residuals and parameters estimated in Step 1 to test the association between each genetic variant and the expression level of the gene of interest.

Questions/Observations

- Supp Fig 7: Is there an explanation for why SAIGE-QTL has smaller (more significant) p-values where there is less signal (-log10(P) in [0, 10]) but TensorQTL has more significant p-values for strong associations?
- Supp Fig 9: concordance in effect sizes looks low (assume absolute value has been taken, but this isn't stated)
- "Given the random effects b, the phenotype Y, which are read counts from RNA sequencing, independently follow [a] Poisson distribution" - empirical evidence?
- Suggestion in SAIGE-QTL docs: "The SCTransform in the SCTransform function in the Seurat R package and/or including log(total read counts) and percentage of MT read counts for each cell as covariates in the Step 1 null model can be used". This makes sense, but given the Poisson model would it not be better to include the total read counts (perhaps with size-factor adjustment) as an offset to the model? (cf. approach in NB models like edgeR)

Major comments

Software

- Installation of this package is currently a major problem
- dependency on `cget`, a python package, is seriously problematic. I had to install cget with mamba and hack paths to allow R to see cget and use it for installation.
- `Sys.setenv(PATH = paste("/mnt/beegfs/users/dmccarthy/miniconda3/bin", Sys.getenv("PATH"), sep = .Platform$path.sep))`
- The entire experience made me very angry
- Running the package through the Docker image failed - trying to run the provided scripts as indicated in the documentation yielded errors that reported that the SAIGEQTL package was not (correctly) installed and loadable within the Docker image. Obviously that is a major concern in the Docker image that you as the authors present to users.
- I can see in the GitHub repository that there are directories and items that should not be in an R pkg (see some non .R files in the R/ directory, among others). Most concerning is the apparent dependency on running this package within a conda environment. In general, R and conda do not play nicely together, so this should *absolutely* be avoided. R does not/should not need any python dependencies or environments to compile C code and install it with the R package. If your approach to installation requires a conda environment then you need to change your approach.
- The R package should be submitted to and hosted on Bioconductor (best option) or CRAN (second-best option). It's not a viable long-term approach to have the Github repository as the default/only source for installing the package. The Bioconductor standards and daily compilation ensure better practices for the developer and a much better user experience. CRAN is similar (but less rigorous) and less focused on bioinformatics packages, so a less-preferred alternative.
- it would be more convenient to use the `remotes` package (https://github.com/r-lib/remotes) as the recommended way to install the R pkg from source in the current documentation:
- `remotes::install_github("https://github.com/weizhou0/qtl")`
- (minor) It's unnerving that the Github repo is named `qtl` but the package name is `SAIGEQTL` - please align the Github repo name to the package name

Display items

- Fig 2: Venn diagrams are hard/impossible to read (text too small) - this information would be much more efficiently displayed as a table (said as someone who generally dislikes tables)

Minor comments

Introduction

- L62: "locus" should be "loci"
- abbreviations "eQTL" and "GWASs" feel internally inconsistent
- L76: "lose" should be "loses" I think

Results:

- L156: define “MAC” for complete clarity

Supplementary figures

- Supp Fig 3: B in caption should say “95% confidence intervals”
- Supp Fig 6: text in the figure is too small to read
- Supp Fig 8: looks like caption has been cut off mid-sentence
- Supp Fig 13: this information would be much clearer as a table; text is too small in fig
- Supp Fig 18: another figure that should be table

Supplementary Excel file

- “Stable4” and “Stable5” tabs: “Empirtical” should be “Empirical”
-

Supplementary note

- P1, 2nd para: “mean an variance”
- throughout - lines starting with a comma, where the comma should be at the end of the equation on the previous line (in general, punctuation of and around equations should be tidied up in many instances)
- Equations on the bottom half of p2 need more punctuation or structure to make sense to the reader
- Repeated sentences “Computation of the variance…” in 1st para of Section 2 at the bottom of P4. Looks like some text might have been lost in a copy-paste error?
- Last sentence of P5: upside down exclamation mark should presumably be “<”
- P6, first para: “Given the random effects b, the phenotype Y, which are read counts from RNA sequencing, independently follow [a] Poisson distribution” (missing the “a”)
- P6, first para: should be “…weighted sum of independent Poisson random variables.”
- P6, Section 3: punctuation needs tidying up in many spots (e.g., missing/added spaces)
- P6, Section 3: \xi does not appear to be defined - please do so for clarity for the reader
- P7, second sentence: Missing reference
- P7, third sentence: should be “5% of genetic variants”
- P7, fifth sentence: I think you want to add “simultaneously” at the end of the sentence
- P7, sixth sentence: “particukary”
- References: some formatting errors can be tidied up, e.g., “reml” in article title, lower-case middle initials, incorrect quotation marks, “g x e analysis”, “uk”, inconsistent capitalisation and similar

Reviewer #3 (Remarks on code availability):

I was able to review the code on Github and could reach some conclusions about (issues with) the implementation by looking at that code.

However, I was not able to install or run the software, which is a dealbreaker for me for further, deeper evaluation of the method, its results, and realistic potential to make an impact in the field. If users cannot easily and reliably install/run the software then, obviously, they will not use it and the breakthroughs made in this work would be for nought.

Version 3:

Decision Letter:

Our ref: NG-A65894R2

22nd July 2026

Dear Wei,

Your revised manuscript "Efficient and accurate mixed model association tool for single-cell eQTL analysis" (NG-TR65894R2) has been seen by Reviewer #1. (Reviewer #3 was also asked to comment on the revision but has not submitted a review to date.) As you will see from the comments below, Reviewer #1 is satisfied with the revision and has no further requests, and therefore we will be happy in principle to publish your work in Nature Genetics as an Article pending final revisions to comply with our editorial and formatting guidelines.

We are now performing detailed checks on your paper, and we will send you a checklist detailing our editorial and formatting requirements soon. Please do not upload the final materials or make any revisions until you receive this additional information from us.

Thank you again for your interest in Nature Genetics. Please do not hesitate to contact me if you have any questions.

Sincerely,
Kyle

Kyle Vogan, PhD
Senior Editor
Nature Genetics
https://orcid.org/0000-0001-9565-9665

Reviewer #1 (Remarks to the Author):

Thank you very much for all responses, which addressed my comments. I do not have further ones.

INTERNAL USE ONLY AIP modules required: ["Clinical","Life Science"]

Open Access This Peer Review File is licensed under a Creative Commons Attribution 4.0 International License, which permits use, sharing, adaptation, distribution and reproduction in any medium or format, as long as you give appropriate credit to the original author(s) and the source, provide a link to the Creative Commons license, and indicate if changes were made.
In cases where reviewers are anonymous, credit should be given to 'Anonymous Referee' and the source.
The images or other third party material in this Peer Review File are included in the article's Creative Commons license, unless indicated otherwise in a credit line to the material. If material is not included in the article's Creative Commons license and your intended use is not permitted by statutory regulation or exceeds the permitted use, you will need to obtain permission directly from the copyright holder.
To view a copy of this license, visit https://creativecommons.org/licenses/by/4.0/

Response to Reviewers Comments

Title: Efficient and accurate mixed model association tool for single-cell eQTL analysis

We thank the reviewers and editors for their thoughtful and constructive comments that helped improve the manuscript. Below are our detailed responses (in bold). Quoted text from the manuscript is highlighted in blue. To distinguish comments and responses, comments are italicized. In the main manuscript, added or revised text is highlighted in blue.

We have addressed all of the prioritized set of points requested by the editorial team. Please see below. More detailed explanations can be found in our response to each reviewer's comment. The manuscript has been substantially revised, with major improvements to software usability, installation robustness, benchmarking, and methodological clarification.

Point 1. address all technical concerns related to the software package and its implementation

We fully acknowledge the seriousness of the concerns raised regarding software installation, usability, and long-term sustainability. We have substantially revised the SAIGE-QTL software and its distribution to address all installation, usability, and implementation concerns raised by the reviewers.

Specifically, we removed problematic dependencies (including cget) and eliminated any requirement for conda or Python environments for standard R installation. The package structure was reorganized to conform to CRAN/Bioconductor conventions, and nonstandard files were removed. The Docker image was rebuilt and validated to ensure that the package is correctly installed, loadable, and executable.

We now provide multiple supported installation options, including a functional Docker image, pre-built Linux and macOS binaries, and standard R source installation (with or without remotes). The GitHub repository has been renamed to match the package name (SAIGEQTL).

Finally, we are actively preparing a Bioconductor submission, with the package now structured and documented to meet Bioconductor requirements.

Together, these changes ensure that SAIGE-QTL is now robust, easily installable, and suitable for long-term community use. We will continue to maintain and update the documentation website and GitHub repository to support long-term usability.

Point 2. perform additional benchmarking studies

We added extensive new benchmarking analyses to evaluate the statistical and computational performance of SAIGE-QTL relative to existing methods. These include expanded simulation studies comparing SAIGE-QTL with pseudobulk approaches (TensorQTL and the more recent jaxQTL) as well as single-cell methods that directly model counts (glmer and CellRegMap-association), across a broad range of expression sparsity levels, cell-type abundances, sampling designs, and effect sizes. We assessed both type I error calibration and empirical power under stringent significance thresholds.

Importantly, we performed additional simulations that specifically reflect study designs commonly observed in real single-cell data, including variability in the number of cells per donor and the presence

of cell-level covariates. Under these settings, SAIGE-QTL consistently showed improved power relative to pseudobulk approaches, highlighting the advantage of modeling cell-level information directly rather than aggregating counts at the donor level.

We further added direct comparisons of runtime and memory usage with existing pseudobulk and single-cell methods and reported the computational cost of rare-variant set-based tests. These results are presented in new Results subsections as well as in Supplementary Figure 19-21 and Supplementary Table 9 and 10.

Point 3. extend the analyses where feasible

In our original submission, SAIGE-QTL was applied to two additional single-cell RNA-seq datasets, one from non-blood tissue and another from blood, to demonstrate its broad applicability and robustness. Comparisons in these datasets remain focused on SAIGE-QTL and TensorQTL, which is commonly used in single-cell eQTL studies, while other approaches (e.g., Poisson mixed models in glmer or Gaussian linear models in CellRegMap-assoc) are less computationally practical at this scale, and pseudobulk methods such as jaxQTL showed inflated type I error in our simulations.

Separately, in the TenK10K study, we applied SAIGE-QTL's rare-variant burden and SKAT tests, highlighting the scalability of SAIGE-QTL for single-cell eQTL analyses. These analyses complement the methodological contributions and provide novel biological insights, including disease-relevant loci identified via GWAS integration and colocalization(Cuomo et al. 2025).

A summary paragraph has been added to the Discussion to clarify how such extended analyses using SAIGE-QTL complement the methodological contributions.

"Beyond methodological advances, applying SAIGE-QTL to real data enabled several novel biological insights. Integration with GWAS via Mendelian randomization identified additional disease-relevant loci acting through cell-type-specific expression, and the scalable framework enabled genome-wide *trans*-eQTL discovery. In addition, SAIGE-QTL's rare-variant burden and SKAT tests have been applied to whole-genome sequencing data in the TenK10K Phase 1 study, enabling in-depth characterization of rare-variant effects on single-cell expression(Cuomo et al. 2025)."

Referee expertise:

Referee #1: Genetics, molecular QTLs, single-cell analyses

Referee #3: Genetics, molecular QTLs, single-cell analyses

Reviewers' Comments:

Reviewer #1 (Remarks to the Author):

Zhou et al. presented a computational tool for mapping single-cell eQTL at scale and showed the improvement of statistical power over the 'pseudobulk' approach implemented in TensorQTL. However, compared to existing single-cell eQTL methods, there were no significant improvements in the statistical power but computational speed. In general, there is lack of novelty in methodology development (such as rare variant association).

We thank the reviewer for their careful evaluation of our work and for raising the important concern regarding methodological novelty. We appreciate the opportunity to clarify the contributions of SAIGE-QTL and to better articulate its significance for single-cell eQTL mapping.

We agree that several methods have been proposed to directly model single-cell gene expression for eQTL analysis, including representative approaches such as CellRegMap. However, these methods rely on linear regression frameworks, which are not ideal for single-cell RNA-seq read count data that are discrete, sparse, and highly skewed. In contrast, SAIGE-QTL adopts a Poisson mixed-effects model that more appropriately reflects the data-generating process of single-cell expression profiles while accounting for repeated measurements from the same donor and genetic relatedness across donors.

Although Poisson mixed models for count data can, in principle, be fitted using existing tools such as the glmer function in the R package lme4, such implementations are not computationally feasible for population-scale single-cell eQTL mapping. In our benchmarks, glmer was over 244 times slower than SAIGE-QTL for *cis*-eQTL mapping (see the subsection "Performance of SAIGE-QTL compared to existing single-cell QTL methods" in the Results section and Supplementary Table 9) and did not scale to datasets of contemporary size, making genome-wide analyses impractical. With further optimizations in SAIGE-QTL for *trans*-eQTL mapping that substantially reduces the computational overhead of reading genotypes, it took SAIGE-QTL 719 CPU hours to conduct genome-wide scan on 8 million genetic variants for 20,000 genes in the naive B cells in OneK1K (Supplementary Table 2), while it would take 10,108 CPU years to finish the analysis using glmer (123,153 times slower). Thus, while appropriate statistical models exist in theory, they cannot be effectively applied to current large-scale single-cell RNA-seq data without a scalable implementation. Accordingly, the primary novelty of SAIGE-QTL lies not in proposing a new statistical model per se, but in providing a robust and highly scalable framework that enables genome-wide single-cell eQTL mapping without pseudobulk aggregation. SAIGE-QTL is designed to efficiently analyze millions of cells, thousands of individuals, and millions of variants, enabling both *cis*- and *trans*-eQTL mapping. These analyses are largely infeasible with existing single-cell methods due to computational constraints.

In addition, SAIGE-QTL extends efficient set-based rare variant association tests to single-cell gene expression data. While rare variant methods are well established for complex human traits and diseases, their application to gene expression, particularly at single-cell resolution, remains largely unexplored. This extension is non-trivial given the combination of non-Gaussian count data, repeated measures (multiple cells per individual), and massive testing burden (thousands of genes tested rather than one or few complex traits) inherent to single-cell eQTL studies.

Finally, to the best of our knowledge, this work provides one of the first large-scale evaluations of eQTL mapping directly at single-cell resolution and comprehensively demonstrates improved statistical power relative to the widely used pseudobulk approach. We have revised the manuscript to better emphasize these points and clarify how SAIGE-QTL addresses key limitations of existing approaches. We thank the reviewer again for their constructive feedback, which has helped strengthen the presentation of our work.

To speed up the tool, the authors did not consider the GPU-based acceleration, which represents the current trend of new genomic tools development.

We thank the reviewer for this valuable suggestion. During the revision of this manuscript, we have continued to optimize the computational efficiency of SAIGE-QTL. In particular, we now leverage the Sherman-Morrison-Woodbury (SMW) formula to efficiently compute the product of the inverse of an N x N matrix and an N x 1 vector at each iteration of model fitting, where N is the number of cells and can reach tens of millions (See subsection "Estimating the variance component and other model parameters (Step 1)" of the Methods section). For studies with unrelated donors, *e.g.*, OneK1K, the SMW formulation enables closed-form, block-wise computations with optimal O(N) complexity, in contrast to the O(N^3) complexity required for direct matrix inversion.

In addition, SAIGE-QTL implements a preconditioned conjugate gradient (PCG) approach to iteratively solve the corresponding linear systems without explicitly inverting the N×N matrices. By default, SAIGE-QTL automatically selects the optimal solver based on data structure: SMW for single-cell studies with repeated cells per donor, and PCG for bulk studies or when no repeated cell structure is detected. For studies with related donors and when the number of donors is large, accounting for sample relatedness via a genetic relationship matrix (GRM) could induce higher memory usage under SMW. In such cases, SMW automatically falls back to PCG when memory constraints are encountered, or users can explicitly specify PCG (--solverMethod=pcg) for more scalable computation, which provides consistent numeric results to SMW (Supplementary Figure 3).

We agree that GPU-based acceleration represents an important direction for future genomic tool development and recently, GPU implementation has gained success in speeding up fitting the mixed models in SAIGE(Rodriguez et al. 2026). We will continue to explore and incorporate GPU-based optimizations in future versions of SAIGE-QTL.

There is still lack of comprehensive comparisons between SAIGE-QTL and existing both 'pseudobulk' and single-cell eQTL mapping methods in the simulated datasets.

We thank the reviewer for raising this important point. In the revised manuscript, we have added comprehensive simulation-based comparisons between SAIGE-QTL and existing eQTL mapping methods, including both pseudobulk-based and single-cell-based approaches.

For pseudobulk methods, we now systematically compare SAIGE-QTL with two existing pseudobulk approaches using simulation datasets under multiple scenarios: TensorQTL(Taylor-Weiner et al. 2019)**, which is based on a linear regression model, and jaxQTL, a recently proposed pseudobulk eQTL mapping method implemented using a negative binomial regression model that was uploaded on medRxiv during the revision of our manuscript**(Zhang et al. 2025)**. These simulations are based on real single-cell RNA-seq read counts from the OneK1K dataset with permuted genotypes. We evaluated type I error (false discovery) rates across a wide range of significance thresholds ($\alpha = 5 \times 10^{-2}$ to 5×10^{-5}), expression sparsity levels, and cell types with different abundances. In these initial simulations, no cell-level covariates were included in the association models, such that pseudobulk and single-cell methods were compared under comparable baseline conditions. Under this setting, SAIGE-QTL and TensorQTL showed well-controlled type I error across all scenarios, while jaxQTL exhibited modest inflation at more stringent thresholds (Supplementary Figure 19 and Supplementary Table 10). Power evaluations under the same framework showed that SAIGE-QTL consistently achieved the highest empirical power across all scenarios, with more pronounced gains in less abundant cell types (Supplementary Figure 20).**

To explicitly evaluate the primary advantage of single-cell eQTL mapping, which is the ability to model cell-level covariates and heterogeneous numbers of cells per donor, we conducted additional simulations. These simulations systematically varied (i) per-donor cell counts (even versus uneven) and (ii) the effect size of cell-level covariates on gene expression. As shown in Supplementary Figure 21, as donor-level cell count imbalance increases and as the influence of cell-level covariates becomes stronger, the power advantage of SAIGE-QTL over pseudobulk methods becomes substantially more pronounced.

For single-cell eQTL methods, we further compared SAIGE-QTL with alternative approaches that also model single-cell counts directly, including Poisson mixed models implemented via the glmer R function and the CellRegMap-association test. Using *cis*-eQTL mapping results for randomly selected genes in B_{IN} cells, SAIGE-QTL produced highly concordant association statistics and effect size estimates with these methods (Pearson $R^2 \geq 0.98$ for $-\log_{10}$ p-values and effect sizes; Supplementary Fig. 18), demonstrating consistent statistical inference. We note that other methods such as GASPACHO and CellRegMap interaction tests are not directly comparable, as they are designed to identify dynamic or cell state-modulated eQTLs rather than population-level eQTLs, which is the focus of SAIGE-QTL.

We have described the results in the subsections "Performance of SAIGE-QTL compared to existing single-cell QTL methods" and "Performance of SAIGE-QTL compared to existing pseudobulk methods" in the Results section. The details of the simulation studies are described in the subsection "Data simulation for comparing SAIGE-QTL to pseudobulk eQTL methods" in the Methods section.

In abstract: It would be good to provide some details about the improvements of SAIGE-QTL compared to existing 'pseudobulk' and single-cell eQTL tools.

We thank the reviewer for this helpful suggestion. In the revised abstract, we now more explicitly describe the improvements of SAIGE-QTL relative to both pseudobulk and existing single-cell eQTL mapping approaches. Compared to pseudobulk methods, SAIGE-QTL directly models single-cell read counts, enabling joint modeling of donor-level and cell-level covariates, accounting for heterogeneous numbers of cells per donor, and improving statistical efficiency and power while maintaining well-controlled false discovery rates.

We further emphasize that SAIGE-QTL uses a Poisson mixed-model framework that is statistically appropriate for count-based single-cell RNA-seq data. Relative to existing single-cell eQTL methods, which often rely on linear mixed models or computationally intensive R-based implementations of generalized linear mixed models, SAIGE-QTL provides statistically consistent inference with substantially improved scalability, enabling analyses of large single-cell datasets. In addition, SAIGE-QTL uniquely supports rare-variant set-based association testing in the single-cell eQTL setting, extending eQTL analyses beyond common variants and providing a unified framework for both common and rare variant discovery.

In addition, please describe the novel biological findings delivered by SAIGE-QTL when applying it to real data.

We thank the reviewer for this suggestion. In the manuscript, we more clearly describe the biological insights enabled by applying SAIGE-QTL to real single-cell RNA-seq data.

First, integrating SAIGE-QTL eQTL results across 14 immune cell types with GWAS summary statistics for four autoimmune diseases, SAIGE-QTL identified more disease-associated genes in cell types and additional loci compared to pseudobulk eQTL mapping, including known autoimmune genes such as *CARD9* (monocytes) and *CD40* (plasma cells), as presented in the subsection "SAIGE-QTL identified additional immune disease-associated loci mediated by gene expression" in the Results section.

Second, due to its scalability, SAIGE-QTL enabled genome-wide *trans*-eQTL scans, identifying hundreds of *trans* associations across multiple immune cell types and revealing long-range regulatory effects, as described in the subsection "SAIGE-QTL enables genome-wide scan for identifying *Trans*-eQTLs" in the Results section.

Third, we used SAIGE-QTL's rare-variant burden and SKAT tests to identify rare-variant eQTLs in the OneK1K dataset. Across 14 cell types, we tested 6,779,365 rare variants (MAF ≤ 5%) within ±1 Mb of each gene and identified 5,541 rare-variant eGenes (2,317 unique genes), of which 483 (21%) remained significant after conditioning on common eQTLs (Supplementary Figure 16 and 17). We also demonstrated that distance-to-TSS-based weighting increases discovery substantially compared with equal or Beta(1,25) weighting (Figure 4B). These results are presented in the subsection "Rare-variant eQTL signals identified by SAIGE-QTL" in the Results section.

Notably, SAIGE-QTL has since been applied to a larger whole-genome sequencing dataset (TenK10K Phase 1), enabling systematic characterization of rare-variant effects on single-cell expression(Cuomo et al. 2025)**.**

To highlight these findings more clearly, we added a short summary paragraph to the Discussion emphasizing the biological discoveries enabled by SAIGE-QTL.

"Beyond methodological advances, applying SAIGE-QTL to real data enabled several novel biological insights. Integration with GWAS via Mendelian randomization identified additional disease-relevant loci acting through cell-type-specific expression, and the scalable framework enabled genome-wide *trans*-eQTL discovery. In addition, SAIGE-QTL's rare-variant burden and SKAT tests have been applied to whole-genome sequencing data in the TenK10K Phase 1 study, enabling in-depth characterization of rare-variant effects on single-cell expression(Cuomo et al. 2025)."

In the computation and memory cost: Please compare SAIGE-QTL with other existing methods, including TensorQTL.

We thank the reviewer for the suggestion to provide a more direct comparison of computation and memory costs between SAIGE-QTL and existing single-cell and pseudobulk eQTL methods.

In the revised manuscript, we added two dedicated benchmarking sections that compare SAIGE-QTL with existing methods that directly model single-cell counts (glmer in lme4 and CellRegMap-association) in the subsection "Performance of SAIGE-QTL compared to existing single-cell QTL methods", as well as pseudobulk approaches (TensorQTL and jaxQTL) in the subsection "Performance of SAIGE-QTL compared to existing pseudobulk methods" in the Results section. For the benchmark, we performed *cis*-eQTL mapping for 100 randomly selected genes in innate and naïve B cells ("B_{IN}"), using 397,291 markers with MAF > 5% (Supplementary Table 9).

Compared to other single-cell count-based methods, SAIGE-QTL achieves substantially improved computational efficiency. Specifically, SAIGE-QTL reduced runtime by ~244× compared to glmer (38 min

+ 16 min for SAIGE-QTL vs. 220 hrs for glmer using one CPU), while producing highly concordant results (Pearson R^2 = 0.991 for –log10 p-values and 0.998 for effect sizes). CellRegMap-association is implemented within CellRegMap, which was primarily designed for dynamic eQTL mapping rather than optimized for standard association testing (according to the developers). Consequently, its computational performance is not directly comparable to SAIGE-QTL, which is developed for scalable single-cell association analyses. *Cis*-eQTL mapping for a randomly selected gene can therefore costs hundreds of CPU hours with CellRegMap-association in our benchmark. Despite using a more complex model (generalized linear mixed Poisson model vs. linear mixed model), SAIGE-QTL achieves improved scalability, analyzing 100 genes in approximately one hour.

Compared to pseudobulk methods, SAIGE-QTL is slower but far more memory-efficient. TensorQTL and jaxQTL are approximately 4.4× and 4.0× faster than SAIGE-QTL, respectively, which is expected because they operate on collapsed donor-level data rather than cell-level counts. Importantly, SAIGE-QTL requires <1 GB memory, compared with 6.3 GB for TensorQTL and 36.5 GB for jaxQTL, demonstrating that direct cell-level modeling can be highly memory-efficient.

Please report the computation cost of set-based tests for rare variants.

We thank the reviewer for the suggestion. In the revised manuscript, we added a paragraph in the subsection "Performance of SAIGE-QTL compared to existing single-cell QTL methods" to report the computation cost of rare-variant set-based tests.

Specifically, we benchmarked SAIGE-QTL's set-based tests using the same 100 randomly selected genes in B_{IN} cells, where the number of rare variants (MAF < 5%) per gene ranges from 480 to 3,812 (mean ≈ 1,489). We found that the set-based tests required ~23 minutes of runtime and 1.7 GB of memory using a single CPU to analyze 100 genes (Supplementary Table 9). Extrapolating from this benchmark, rare-variant eQTL mapping for ~22,000 genes in B_{IN} cells is estimated to require ~85 CPU hours.These results demonstrate that SAIGE-QTL enables scalable rare-variant set-based testing in single-cell data while maintaining manageable computational and memory requirements.

Line 223: Not sure if 150 times permutation was enough. Please justify.

We thank the reviewer for requesting clarification regarding the number of permutations used in our simulation. We performed 150 independent permutations to ensure a sufficiently large number of independent tests under the null hypothesis for evaluating type I error rates (false discovery calibration), including at quite stringent significance thresholds low to 1×10^{-7}. When aggregating results across all cell types, genes, and sparsity bins, this design yields well over $1\text{x}10^{9}$ total tests. At this scale, even under the most stringent significance threshold examined (1×10^{-7}), we would expect on the order of 100 false-positive associations by chance alone, providing adequate resolution to assess type I error control. The exact number of tests performed for each cell type-sparsity bin combination is reported in Supplementary Table 5.

Line 216 and 228: Did the authors consider heritability of gene expression when selecting genes?

No, we did not consider heritability when selecting genes for the type I error simulation as genotypes were permuted to simulate under the null hypothesis. We then considered different effect sizes in power simulations, where higher effect size and MAF are corresponding to higher heritability of gene

expression. We simulated gene expressions through Poisson mixed model, where on the linear predictor (latent) scale, the genetic variants equals $\beta_{□}{}^{2}□□□(□)$ with $□□□(□) = 2□(1 - □)$ for MAF p, and donor random-effect variance $□_{□□} = 0.2$ are employed in all simulations, the corresponding latent SNP-heritability can be approximated by $□^{2}{}_{□□□□□□} = □_{□}{}^{2}□□□(□)/(□_{□}{}^{2}□□□(□) + □_{□□})$. Because the observed counts follow a Poisson distribution, observed-scale heritability can be affected by mean-dependent Poisson variance and will be smaller than these latent values. But the mapping above makes clear that our simulation grid covers low to high genetic contributions to expression. We have therefore reported power and calibration across this wide and explicitly defined heritability range rather than preselecting genes with particular measured heritability. Type I error calibration and power simulation findings presented in Supplementary Table 10, and Supplementary Figures 19 and 20.

It would be good to compare SAIGE-QTL with other existing methods across all the simulated scenarios. In which scenarios did SAIGE-QTL outperform the 'pseudobulk' approach?

We thank the reviewer for suggesting a more comprehensive comparison with existing methods and for prompting us to clarify the scenarios in which SAIGE-QTL outperforms pseudobulk approaches. We agree and have added systematic comparisons across all simulated scenarios in the Methods and Supplementary Figures 19–21.

We compared SAIGE-QTL with two representative pseudobulk methods: TensorQTL (using rank-based inverse normal–transformed phenotypes)(Taylor-Weiner et al. 2019) **and jaxQTL (using the Negative Binomial model recommended by the authors in their preprint)**(Zhang et al. 2025)**. We evaluated both type I error calibration and empirical power across varying cell-type abundance, expression sparsity, sampling designs, and effects of cell-level covariates.**

Across all scenarios, SAIGE-QTL and TensorQTL exhibited well-controlled type I error rates, whereas jaxQTL showed modest inflation, especially at more stringent significance thresholds (e.g., $\alpha \leq 5\times10^{-4}$). In contrast, SAIGE-QTL consistently maintained strict adherence to nominal type I error rates, even at very stringent alpha levels, while achieving high empirical power.

Importantly, we identified two scenarios in which SAIGE-QTL demonstrates clear advantages over pseudobulk methods. First, when per-donor cell counts are highly uneven, as observed in the OneK1K cohort, SAIGE-QTL gains power by directly modeling cell-level read counts and their associated uncertainty, whereas pseudobulk approaches collapse this information to the donor level, obscuring the uneven information from different donors. Second, when cell-level covariates influence gene expression, SAIGE-QTL substantially outperforms pseudobulk methods, which aggregate such covariates across cells and therefore cannot fully capture within-donor heterogeneity.

Together, these results demonstrate that SAIGE-QTL leverages cell-level resolution to achieve robust calibration and improved power in biologically realistic settings, while remaining computationally scalable for large datasets.

Line 246: Why 250 times permutation? Please justify.

We thank the reviewer for requesting clarification on the number of permutations used in our simulation study for set-based tests. We performed 250 independent permutations to generate a sufficiently large number of null tests for evaluating type I error control, including at stringent

significance thresholds down to 5×10^{-4}. Together, this design yields ~8×10^{4} total gene-based tests for each sparsity level at each representative cell type , providing adequate resolution to assess type I error calibration. The permuted results for set-based tests are shown in Supplementary Figure 6 (previously Supplementary Figure 5).

We have also clarified this point in the Results section by revising the sentence to:

"The tests were repeated for 250 times with permuted individual IDs to generate a large number of null gene-based tests (~ 8x10^{4}) for assessing type I error control at relatively stringent significance thresholds down to 5×10^{-4} for each gene expression sparsity level and representative cell type, and we observed well calibrated set-based tests in SAIGE-QTL for genes from different sparsity groups across three cell types as presented in **Supplementary Figure 6.** "

Line 348-357: It would be good to compare SAIGE-QTL with commonly used GWAS methods (e.g., GCTA) in trans-eQTL mapping.

We thank the reviewer for this suggestion. GWAS methods, such as GCTA, are designed for quantitative phenotypes under a linear mixed model framework or binary phenotypes under a logistic mixed model framework. While they can in principle accommodate repeated measurements through additional random effects, they are not well suited for discrete count data or the large number of repeated cell-level observations typical of single-cell RNA-seq experiments and do not explicitly model cell-level covariates or the within-donor cell–cell correlation structure inherent to single-cell data.

In contrast, SAIGE-QTL is specifically designed for single-cell eQTL mapping and directly models read counts using Poisson mixed models, accounting for both within-donor cell–cell correlation and donor relatedness, if any. In addition, SAIGE-QTL incorporates computational optimizations that enable efficient genome-wide *trans*-eQTL mapping by jointly analyzing hundreds or thousands of genes and reducing genotype-processing overhead. For these reasons, a direct comparison between SAIGE-QTL and GWAS methods such as GCTA for single-cell *trans*-eQTL mapping is not straightforward or practical.

Line 359-375: It would be good if the authors could biologically interpret the findings from rare-variant eQTL mapping. Will these results help us better understand the genetics of complex phenotypes?

Thank you for the comment. In the OneK1K study that we analyzed using SAIGE-QTL in our manuscript, the rare-variant association test was somewhat limited to the relatively small number of rare variants that could be called from genotype array data, and thus mostly serves as a proof-of-principle application of the method. However, SAIGE-QTL's burden and SKAT tests were recently applied to rare variants called from whole-genome sequencing data of nearly 2,000 individuals as part of the TenK10K phase 1 paper (Cuomo et al. 2025)**. There, the authors were able to perform a more in-depth analysis and interpretation of the effects of rare variation on single-cell resolved gene expression. First, SKAT tests identified the most signals, consistent with the fact that these are better suited for capturing cases where variants may have both positive and negative effects on gene expression, which we expect in gene regulation. Second, subsetting to variants sitting in genomic regions annotated to be regulatory or in open chromatin regions boosted the power of discovery. However, even larger studies will be**

necessary to further divide variants into specific sets of annotations (promoters vs enhancers, etc) and to confidently link these observations to complex traits and diseases.
Line 411: please justify why not compare SAIGE-QTL with other single-cell eQTL methods in these two datasets?

Thank you for this comment. Our benchmark comparing SAIGE-QTL to other single-cell eQTL mapping tools demonstrates that these methods are not computationally feasible for the full real datasets (results in Supplementary Table 12). The glmer function in the lme4 R package, which uses the same Poisson mixed model and was previously used by Nathan et al.(Nathan et al. 2022)**, is substantially slower than SAIGE-QTL (≈244× slower). CellRegMap-association**(Cuomo et al. 2022)**, which uses a linear mixed model for counts, can be applied only to a small set of markers rather than the entire *cis*-region; for example, it requires approximately 70 hours and 28 GB to analyze a single gene with 6000 markers in B_{IN} cells in OneK1K. Other single-cell eQTL methods, such as GASPACHO**(Kumasaka et al. 2023) **and CellRegMap proper (interaction test)**(Cuomo et al. 2022)**, focus on identifying "dynamic" eQTLs along continuous cell states rather than standard *cis*-eQTLs within a population, which is the focus of SAIGE-QTL. These methods are also not computationally feasible for full *cis*-region analyses in our real datasets and are therefore limited to small marker subsets using a two-step strategy.**

Reviewer #3 (Remarks to the Author):

Zhou et al introduce introduce a new method and software tool, SAIGE-QTL, for mapping expression quantitative trait loci from single-cell RNA-sequencing data. The paper has great potential to be an important contribution to the field, with important new methodologies that open up exciting new possibilities for single-cell molecular QTL mapping.

However, my attempts to complete a thorough review of the work were stymied by persistent failures in installing (the R package) or running (via the Docker image) the software (all replicated by a senior postdoc in my group). As such, I provide my initial comments and feedback here, but would need to engage with a revision that provides easily installable and runnable software for SAIGE-QTL to evaluate the paper and method sufficiently to the degree required for publication in Nature Genetics.

I would be very happy to review this paper again when provided with a working version of the software that I can install, run, and test out for myself as despite my current frustrations with the software implementation I can see that this is work of potentially high impact and importance.

Key contributions of this work:

- feasible eQTL mapping using single-cell resolution data (not requiring pseudobulking)
- speed and scalability of the method relative to existing options
- set tests to map effects of rare variants
- extension of SAIGE (Scalable and Accurate Implementation of GEneralized mixed model), SAIGE-GENE and SAIGE-GENE+ to eQTL mapping
- enables trans-eQTL mapping

Method:

1. Fit null Poisson MM, which includes individual-level covariates, such as sex, age, and ancestry PCs for donors, and cell-specific covariates, such as expression-based PEER factors, as fixed effects and accounts for intra-individual cell-to-cell variability using a random effect
2. a) Testing for the association between each common genetic variant (e.g., MAF > 5%) and gene expression using score tests
b) conducting set-based tests for less frequent/rare variants with e.g., MAF < 5%, including Burden tests, SKAT, and ACAT-V, each having superior power in various scenarios of effect directions and sparsity levels followed by an ACAT-O test to combine p-values (see 10.1016/j.ajhg.2019.01.002)
3. calculating a gene-level p-value for each cis-eQTL region using the ACAT-V test, which combines variant-level p-values (from Step 2a) using a Cauchy combination that is robust to the correlation among individual p-values and the sparsity of effects

Step 1 iteratively estimates the model parameters using the penalised quasi-likelihood (PQL) method and the computationally efficient average information restricted maximum likelihood algorithm. Once the null model is fitted for a gene in a cell type, it can be used to test the association of any genetic markers or regions for that gene's expression in that cell type.

In Step 2, for common variants, the score test is performed based on model residuals and parameters estimated in Step 1 to test the association between each genetic variant and the expression level of the gene of interest.

# Questions/Observations

- Supp Fig 7: Is there an explanation for why SAIGE-QTL has smaller (more significant) p-values where there is less signal (-log10(P) in [0, 10]) but TensorQTL has more significant p-values for strong associations?

Thank you for this insightful question. We believe the pattern in Supplementary Figure 8 (original Supplementary Figure 7) reflects differences in modeling assumptions and the information used by the two approaches. TensorQTL uses pseudobulk mean expression and a Gaussian linear model, which can yield extremely significant p-values for strong eQTL signals because aggregation reduces noise and increases effective sample size. In contrast, SAIGE-QTL models cell-level counts directly using a Poisson mixed model, which captures cell-to-cell variability; for very strong associations, this additional variability can modestly reduce the significance of top signals (*e.g.*, 10^{-200} vs. 10^{-300}), even though the association remains highly significant.

Conversely, the stronger results for weaker signals (−log10(p) in [0, 10]) are consistent with our simulations showing that SAIGE-QTL can have higher power than pseudobulk methods. By modeling counts at the single-cell level rather than collapsing to donor-level summaries, SAIGE-QTL can better leverage subtle genetic effects that may be diluted by aggregation, especially for sparse genes. This advantage is expected to increase when cell-level covariates (*e.g.*, cell states) influence expression, as shown in Supplementary Figure 21; however, in the OneK1K analysis we used only donor-level covariates to match the original analyses that used a pseudobulk approach(Yazar et al. 2022)**.**

Overall, SAIGE-QTL is designed to capture cell level information and improve discovery of eQTLs that may be missed by pseudobulk approaches, particularly for modest effects and sparse expression patterns.

- Supp Fig 9: concordance in effect sizes looks low (assume absolute value has been taken, but this isn't stated)

We thank the reviewer for raising this point. In the scatter plots, we did not take absolute values of effect sizes. Instead, for each variant we align effect estimates to the same allele, defined as the allele with a positive effect in TensorQTL (i.e., the allele that increases expression in TensorQTL). Under this allele alignment, the correlation coefficient primarily reflects agreement in effect magnitude rather than direction. Without this alignment, variants with opposite directions would fall in opposite quadrants and the correlation would be driven by directionality rather than magnitude. We have clarified this point in the figure legend for Supplementary Figure 10 (original Supplementary Figure 9).

Importantly, the plots show that the two methods agree on effect direction: each dot represents the top variant in each eGene in TensorQTL, and all points fall in the positive quadrant. The relatively low correlation coefficients likely reflect that the effect size estimates from the two methods are expressed in different units. TensorQTL reports effect sizes in units of standard deviations of mean gene expression across donors, while SAIGE-QTL reports effect sizes in units of raw single-cell read counts. We have added a sentence at the end of the subsection "SAIGE-QTL identified more eGenes than the pseudobulk approach" in the Results section to clarify this interpretation.

"The low correlation coefficients are likely due to differences in effect size units between methods: TensorQTL reports effect sizes in standard deviations of mean expression across donors, while SAIGE-QTL reports effect sizes in raw single-cell read counts."

- "Given the random effects b, the phenotype Y, which are read counts from RNA sequencing, independently follow [a] Poisson distribution" - empirical evidence?

We thank the reviewer for the comment. In the Poisson mixed model, the conditional Poisson assumption is an intrinsic modeling assumption that defines the null likelihood and the score test. It is this conditional distribution assumption that enables us to derive the score statistic. While RNA-seq counts may exhibit overdispersion, this is handled through the mixed model structure: random effects induce marginal overdispersion and correlation. Therefore, the conditional Poisson assumption remains appropriate for defining the model and deriving the score statistic. Importantly, we have extensively validated the calibration of the score tests through simulations and permutation, demonstrating accurate type I error control under realistic scenarios and included the results in the subsection "Type I error rates/False positive calibration" in the Results section.

- Suggestion in SAIGE-QTL docs: "The SCTransform in the SCTransform function in the Seurat R package and/or including log(total read counts) and percentage of MT read counts for each cell as covariates in the Step 1 null model can be used". This makes sense, but given the Poisson model would it not be better to include the total read counts (perhaps with size-factor adjustment) as an offset to the model? (cf. approach in NB models like edgeR)

We thank the reviewer for this insightful suggestion. SAIGE-QTL already provides an option to include an offset in the Step 1 null model to account for sequencing depth and other technical artifacts across cells. This offset is particularly important when modeling raw single-cell read counts directly, as it ensures that variation in library size is properly controlled and can improve eQTL power. While normalization approaches such as SCTransform can reduce these artifacts, in practice they may not completely remove all sequencing-depth effects. In such cases, including the total read count (or a

size-factor-adjusted version) as an offset provides an additional, robust correction and is recommended for optimal performance.

Major comments

## Software

- Installation of this package is currently a major problem
- dependency on `cget`, a python package, is seriously problematic. I had to install cget with mamba and hack paths to allow R to see cget and use it for installation.
- `Sys.setenv(PATH = paste("/mnt/beegfs/users/dmccarthy/miniconda3/bin", Sys.getenv("PATH"), sep = .Platform$path.sep))`
- The entire experience made me very angry
- Running the package through the Docker image failed - trying to run the provided scripts as indicated in the documentation yielded errors that reported that the SAIGEQTL package was not (correctly) installed and loadable within the Docker image. Obviously that is a major concern in the Docker image that you as the authors present to users.
*- I can see in the GitHub repository that there are directories and items that should not be in an R pkg (see some non .R files in the R/ directory, among others). Most concerning is the apparent dependency on running this package within a conda environment. In general, R and conda do not play nicely together, so this should *absolutely* be avoided. R does not/should not need any python dependencies or environments to compile C code and install it with the R package. If your approach to installation requires a conda environment then you need to change your approach.*
- The R package should be submitted to and hosted on Bioconductor (best option) or CRAN (second-best option). It's not a viable long-term approach to have the Github repository as the default/only source for installing the package. The Bioconductor standards and daily compilation ensure better practices for the developer and a much better user experience. CRAN is similar (but less rigorous) and less focused on bioinformatics packages, so a less-preferred alternative.
- it would be more convenient to use the `remotes` package (https://github.com/r-lib/remotes) as the recommended way to install the R pkg from source in the current documentation:
- `remotes::install_github("[https://github.com/weizhou0/qtl](https://github.com/weizhou0/qtl)")`
- (minor) It's unnerving that the Github repo is named `qtl` but the package name is `SAIGEQTL` - please align the Github repo name to the package name

Thanks for pointing out the misalignment between the repository name (qtl) and package name (SAIGEQTL). We have renamed the repository to SAIGEQTL to eliminate this confusion.

We thank the reviewer for the detailed feedback regarding installation and usability. We fully agree that a robust and frustration-free installation experience is essential, and we have made substantial changes to address these concerns. All installation workflows were validated on clean Linux and macOS systems to ensure reproducible and reliable deployment across platforms. We acknowledge that the previous installation process, including dependencies on cget and a conda environment, caused significant difficulties.

In the revised version, we have removed the cget dependency and eliminated any requirement for a conda environment for standard R installation. The package can now be built using standard R toolchains.

At the same time, we recognize that installing R packages with compiled C++ code require additional system configuration on some platforms. To address this, we now provide multiple installation options. For most users, we recommend the Docker container or pixi-based workflow, which provide automated dependency resolution and the most reliable setup experience. The remaining installation options are primarily intended for developers and advanced users who prefer manual configuration.

1. **Docker/Singularity Installation (Recommended): We have fixed the Docker image issues you encountered. The current image correctly includes all dependencies and the SAIGEQTL package is properly installed and loadable. It can be downloaded and installed following instructions on this page:** **https://weizhou0.github.io/SAIGE-QTL-doc/docs/Installation_docker.html**

2. **Pre-built Linux and macOS Binaries (Recommended): For Linux and macOS users, we provide pre-compiled binaries that eliminate compilation issues entirely and can be installed with standard R commands (https://weizhou0.github.io/SAIGE-QTL-doc/docs/Installation_pixi.html)**

3. **Installation from source code (3a with a pixi-based workflow is recommended):** **For users and developers who prefer installing from the source code, we provide several alternatives (https://weizhou0.github.io/SAIGE-QTL-doc/docs/Installation_sourcecode.html)**

    3a. To support reproducible environments, we provide (and recommend) a pixi-based workflow that automatically resolves dependencies in an isolated environment, without requiring users to manually manage environment configuration. Pixi enables consistent setup across platforms while minimizing user effort.

    3b. Standard R installation after manually downloading the source code and potentially configuring the environment as listed out below if needed based on the user's specific system.

**System Requirements**	
Operating System	Linux (recommended), macOS
Git	For downloading package
C/C++ compiler	Clang with C++11 support, GCC $\geq$ 10.3
OpenMP	(optional) required for parallel processing
GNU make/cmake	Required
**R Requirements**	

R	R $\geq$ 3.5.0
**System Libraries** (Must be installed before R package)	
BLAS/LAPACK	Linear algebra (OpenBLAS recommended)
zlib & zstd	Compression & Zstandard compression
SuperLU	Sparse linear solver
Boost C++ libraries	String algorithms
**R Package Dependencies**	
Rcpp	$\geq$ 1.0.7
RcppArmadillo	$\geq$ 0.10.7.5
RcppParallel	any
RcppEigen	any
RcppNumerical	any
Matrix	any
data.table	any
furrr	any
dbplyr	any
BH	any
**Additional LinkingTo Dependencies**	
SPAtest	3.1.2
SKAT	any

RhpcBLASctl	any
RSQLite	any
dplyr	any
nlme	any
MASS	any
optparse	any
qlcMatrix	any
MetaSKAT	any
fastSave	any

3c. Standard R Installation using remotes:

```
if (!requireNamespace("remotes", quietly = TRUE)) {
  install.packages("remotes")
}
remotes::install_github("weizhou0/SAIGEQTL", dependencies = TRUE)
```

Note that 3b and 3c require only standard R dependencies (Rcpp, RcppArmadillo, etc.) with some system requirements, and do not rely on conda environments or python dependencies. However, they require a system compiler with OpenMP support, which is available by default on Linux systems and may require additional setup on macOS. Other requirements such as a build system (like cmake), and system libraries that needed to be installed prior to R package installation can be looked up from the table above.

Regarding non-R files in R/ directory: We have cleaned up the package structure to conform to standard R package conventions. We agree that Bioconductor is an ideal platform for distributing genomics software and ensuring long-term accessibility. SAIGE-QTL is fully open source and currently available on GitHub with comprehensive documentation, example workflows, and a Docker container supporting reproducible installation. The current repository follows CRAN/Bioconductor guidelines for directory structure and file organization. We will submit the package to Bioconductor to further support long-term distribution and integration with community standards.

We have updated all documentation to: clearly distinguish between required and optional installation dependencies; provide troubleshooting guides for common installation scenarios; and remove references to deprecated conda environment-dependent installation paths.

## Display items

- Fig 2: Venn diagrams are hard/impossible to read (text too small) - this information would be much more efficiently displayed as a table (said as someone who generally dislikes tables)

Thank you for pointing this out. The Venn diagram was originally intended to highlight that SAIGE-QTL replicates the eGenes captured by TensorQTL while also identifying novel ones. To address the readability concern, we have increased the font size of the numbers in the Venn diagram and now additionally present the same information in Supplementary Table 7A.

# Minor comments

## Introduction

- L62: "locus" should be "loci"
- abbreviations "eQTL" and "GWASs" feel internally inconsistent
- L76: "lose" should be "loses" I think

We have fixed these in the revised manuscript.

## Results:

- L156: define "MAC" for complete clarity

We have defined MAC in the revised manuscript.

### Supplementary figures

- Supp Fig 3: B in caption should say "95% confidence intervals"

Supp Fig 4 (original Supp Fig 3): fixed caption now.

- Supp Fig 6: text in the figure is too small to read
Supp Fig 7 (original Supp Fig 6): increased font size now.

- Supp Fig 8: looks like caption has been cut off mid-sentence
Supp Fig 9 (original Supp Fig 8): fixed caption now.

- Supp Fig 13: this information would be much clearer as a table; text is too small in fig
These results are presented in Supplementary Table 8.

- Supp Fig 18: another figure that should be table
Supp Fig 22(original SFig 18): These Venn diagrams correspond to the Supplementary Table 13.

### Supplementary Excel file

- "Stable4" and "Stable5" tabs: "Empirtical" should be "Empirical"

Modified in Stables.

### Supplementary note

- *P1, 2nd para: “mean an variance”*
- *throughout - lines starting with a comma, where the comma should be at the end of the equation on the previous line (in general, punctuation of and around equations should be tidied up in many instances)*
- *Equations on the bottom half of p2 need more punctuation or structure to make sense to the reader*
- *Repeated sentences “Computation of the variance…” in 1st para of Section 2 at the bottom of P4. Looks like some text might have been lost in a copy-paste error?*
- *Last sentence of P5: upside down exclamation mark should presumably be “<”*
- *P6, first para: “Given the random effects b, the phenotype Y, which are read counts from RNA sequencing, independently follow [a] Poisson distribution” (missing the “a”)*
- *P6, first para: should be “…weighted sum of independent Poisson random variables.”*
- *P6, Section 3: punctuation needs tidying up in many spots (e.g., missing/added spaces)*
- *P6, Section 3: \xi does not appear to be defined - please do so for clarity for the reader*
- *P7, second sentence: Missing reference*
- *P7, third sentence: should be “5% of genetic variants”*
- *P7, fifth sentence: I think you want to add “simultaneously” at the end of the sentence*
- *P7, sixth sentence: “particukary”*
- *References: some formatting errors can be tidied up, e.g., “reml” in article title, lower-case middle initials, incorrect quotation marks, “g x e analysis”, “uk”, inconsistent capitalisation and similar*

Thanks to the reviewer for pointing out these issues. We have fixed them in the Supplementary Note.

Reviewer #3 (Remarks on code availability):

I was able to review the code on Github and could reach some conclusions about (issues with) the implementation by looking at that code.

However, I was not able to install or run the software, which is a dealbreaker for me for further, deeper evaluation of the method, its results, and realistic potential to make an impact in the field. If users cannot easily and reliably install/run the software then, obviously, they will not use it and the breakthroughs made in this work would be for nought.

We thank the reviewer for emphasizing the importance of reliable software installation and usability. We fully agree that accessibility is critical for ensuring that the method can be adopted by the community. The installation issues identified in the previous version have now been fully resolved. We redesigned the installation workflow to minimize dependencies and improve robustness, and all installation workflows were validated on clean Linux and macOS systems using fresh environments to ensure reproducible and reliable deployment across platforms.

We have also added step-by-step installation instructions, automated environment configuration, and a Docker container to further simplify setup and ensure consistency across computing environments. The updated repository has been tested to confirm that users can successfully install and run the software without manual intervention. We welcome the reviewer to evaluate the updated installation workflow and software functionality.

References:

Cuomo, Anna S. E., Tobias Heinen, Danai Vagiaki, Danilo Horta, John C. Marioni, and Oliver Stegle. 2022. “CellRegMap: A Statistical Framework for Mapping Context-Specific Regulatory Variants Using scRNA-Seq.” *Molecular Systems Biology* 18 (8): e10663.

Cuomo, Anna S. E., Eleanor Spenceley, Hope A. Tanudisastro, et al. 2025. “Impact of Rare and Common Genetic Variation on Cell Type-Specific Gene Expression in Human Blood.” In *medRxiv*. November 11. https://doi.org/10.1101/2025.03.20.25324352.

Kumasaka, Natsuhiko, Raghd Rostom, Ni Huang, et al. 2023. “Mapping Interindividual Dynamics of Innate Immune Response at Single-Cell Resolution.” *Nature Genetics* 55 (6): 1066–1075.

Nathan, Aparna, Samira Asgari, Kazuyoshi Ishigaki, et al. 2022. “Single-Cell eQTL Models Reveal Dynamic T Cell State Dependence of Disease Loci.” *Nature* 606 (7912): 120–128.

Radhakrishna Rao, C. 1948. “Large Sample Tests of Statistical Hypotheses Concerning Several Parameters with Applications to Problems of Estimation.” *Mathematical Proceedings of the Cambridge Philosophical Society* 44 (1): 50–57.

Rodriguez, Alex, Youngdae Kim, Tarak Nath Nandi, et al. 2026. SAIGE-GPU — Accelerating Genome- and Phenome-Wide Association Studies using GPUs, Bioinformatics, 2026;, btag032, https://doi.org/10.1093/bioinformatics/btag032.

Taylor-Weiner, Amaro, François Aguet, Nicholas J. Haradhvala, et al. 2019. “Scaling Computational Genomics to Millions of Individuals with GPUs.” *Genome Biology* 20 (1): 228.

Wald, Abraham. 1943. “Tests of Statistical Hypotheses Concerning Several Parameters When the Number of Observations Is Large.” *Transactions of the American Mathematical Society* 54 (3): 426–482.

Yazar, Seyhan, Jose Alquicira-Hernandez, Kristof Wing, et al. 2022. “Single-Cell eQTL Mapping Identifies Cell Type-Specific Genetic Control of Autoimmune Disease.” *Science (New York, N.Y.)* 376 (6589): eabf3041.

Zhang, Zixuan Eleanor, Artem Kim, Noah Suboc, Nicholas Mancuso, and Steven Gazal. 2025. “Efficient Count-Based Models Improve Power and Robustness for Large-Scale Single-Cell eQTL Mapping.” In *medRxiv*. March 5. https://doi.org/10.1101/2025.01.18.25320755.
